# Three-dimensional folding dynamics of the *Xenopus tropicalis* genome

**DOI:** 10.1038/s41588-021-00878-z

**Published:** 2021-06-07

**Authors:** Longjian Niu, Wei Shen, Zhaoying Shi, Yongjun Tan, Na He, Jing Wan, Jialei Sun, Yuedong Zhang, Yingzhang Huang, Wenjing Wang, Chao Fang, Jiashuo Li, Piaopiao Zheng, Edwin Cheung, Yonglong Chen, Li Li, Chunhui Hou

**Affiliations:** 1 Department of Biology, School of Life Sciences, Southern University of Science and Technology, Shenzhen, China; 2 Department of Bioinformatics, Huazhong Agricultural University, Wuhan, China; 3 Hubei Key Laboratory of Agricultural Bioinformatics, Huazhong Agricultural University, Wuhan, China; 4 Cancer Centre, Faculty of Health Sciences, University of Macau, Taipa, China; 5 Centre of Precision Medicine Research and Training, Faculty of Health Sciences, University of Macau, Taipa, China

**Keywords:** Developmental biology, Genetics, Cell biology, Computational biology and bioinformatics, Molecular biology

## Abstract

Animal interphase chromosomes are organized into topologically associating domains (TADs). How TADs are formed is not fully understood. Here, we combined high-throughput chromosome conformation capture and gene silencing to obtain insights into TAD dynamics in *Xenopus tropicalis* embryos. First, TAD establishment in *X. tropicalis* is similar to that in mice and flies and does not depend on zygotic genome transcriptional activation. This process is followed by further refinements in active and repressive chromatin compartments and the appearance of loops and stripes. Second, within TADs, higher self-interaction frequencies at one end of the boundary are associated with higher DNA occupancy of the architectural proteins CTCF and Rad21. Third, the chromatin remodeling factor ISWI is required for de novo TAD formation. Finally, TAD structures are variable in different tissues. Our work shows that *X. tropicalis* is a powerful model for chromosome architecture analysis and suggests that chromatin remodeling plays an essential role in de novo TAD establishment.

## Abstract (web summary)

Hi-C and single-molecule sequencing analysis provide an improved assembly of the *Xenopus tropicalis* genome and insights into three-dimensional genome dynamics throughout embryogenesis.

## Main

Interphase chromosomes are partitioned into TADs^1–4^, segregating into the compartments of active or repressive chromatin^5–7^. The structure of TADs is relatively stable and resilient to environmental perturbations^8,9^ and their architecture is evolutionarily conserved in eukaryotes^4,10,11^. Disruption of TAD borders can lead to developmental disorders and even tumorigenesis; this underlines the importance of three-dimensional (3D) genome organization in gene regulation^12–15^.

The establishment of chromatin architecture during embryogenesis provides an initial spatial frame that may guide proper genome organization, chromatin interaction and gene regulation^16^. In fruit flies, mice and humans, TADs form at the zygotic genome activation (ZGA) stage and continually consolidate through early embryo development^16–20^. However, in zebrafish, TADs are already preformed before ZGA, subsequently lost and then reestablished in later developmental stages^21^. The difference in TAD formation between species thus raises the question of whether this process is evolutionarily conserved.

DNA loop extrusion mediated by the cohesin complex was recently reported in several in vitro studies^22,23^ and proposed as a functional mechanism underlying TAD establishment^24–26^. In cultured cells, deletion of the cohesin complex component double-strand-break repair protein rad21 homolog (Rad21) alone was enough to abolish the establishment of TADs^27^. Other proteins, including CCCTC-binding factor (CTCF), the cohesin antagonist Wings apart-like protein homolog (WAPL) and its partner PDS5, also participate in TAD regulation and loop structure formation^28,29^. CTCF loss disrupts TAD insulation but not higher-order genomic compartmentalization^30^. Likewise, TAD formation during mouse^31^ and human embryogenesis^32^ requires Rad21 and CTCF, respectively. These findings suggest that TAD formation in cultured and embryonic nuclei is conserved and may require both factors through cohesin-mediated extrusion that stops at convergent CTCF binding sites^11,33,34^.

Interestingly, transcription appears to be dispensable for TAD formation at ZGA in fruit flies and mice^18–20^ but not in humans^32^. Heinz et al.^35^ showed that transcription disrupts TAD borders by displacing cohesin and CTCF during influenza A virus infection, while others found that transcription drives the formation of domain borders in *Caulobacter* cells^36^. These opposing findings suggest that the role of transcription in TAD formation is likely context-dependent or regulated by undefined factors.

How TADs are formed during embryogenesis is still not fully clear. During *X. tropicalis* embryogenesis, major ZGA occurs after 12 synchronous cell cycles^37^ at the mid-blastula transition (MBT) (stage 8+) stage when S and gap phases appear and interphase lengthens^38,39^. More than 1,000 genes are activated before MBT^40,41^, while most of the zygotic genome is transcriptionally silent. To examine and assess the role of specific factors in the de novo establishment of chromatin architecture in the *Xenopus* zygote, morpholinos can be used to block the new translation of target proteins. In this study, we examined chromosome conformation change across multiple developmental stages in wild-type (WT) *X. tropicalis* embryos and embryos where RNA polymerase II (Pol II), CTCF, Rad21 or the chromatin remodeling factor ISWI translation was inhibited. Our work revealed that in *Xenopus*, TADs appear at ZGA and are followed by the sequential establishment of loop and stripe structures in later developmental stages. We found that TAD formation requires CTCF and Rad21. We also demonstrated that ISWI is required for both the establishment of TADs and embryo development. Interestingly, we showed that chromatin interaction directionality is almost always stronger on one side of the TAD border and is accompanied by a higher enrichment of CTCF and Rad21 binding. Finally, we showed that the genome architecture of *X. tropicalis* is variable in different tissues.

## Results

### De novo assembly of the *X. tropicalis* genome

**Fig. 1:**
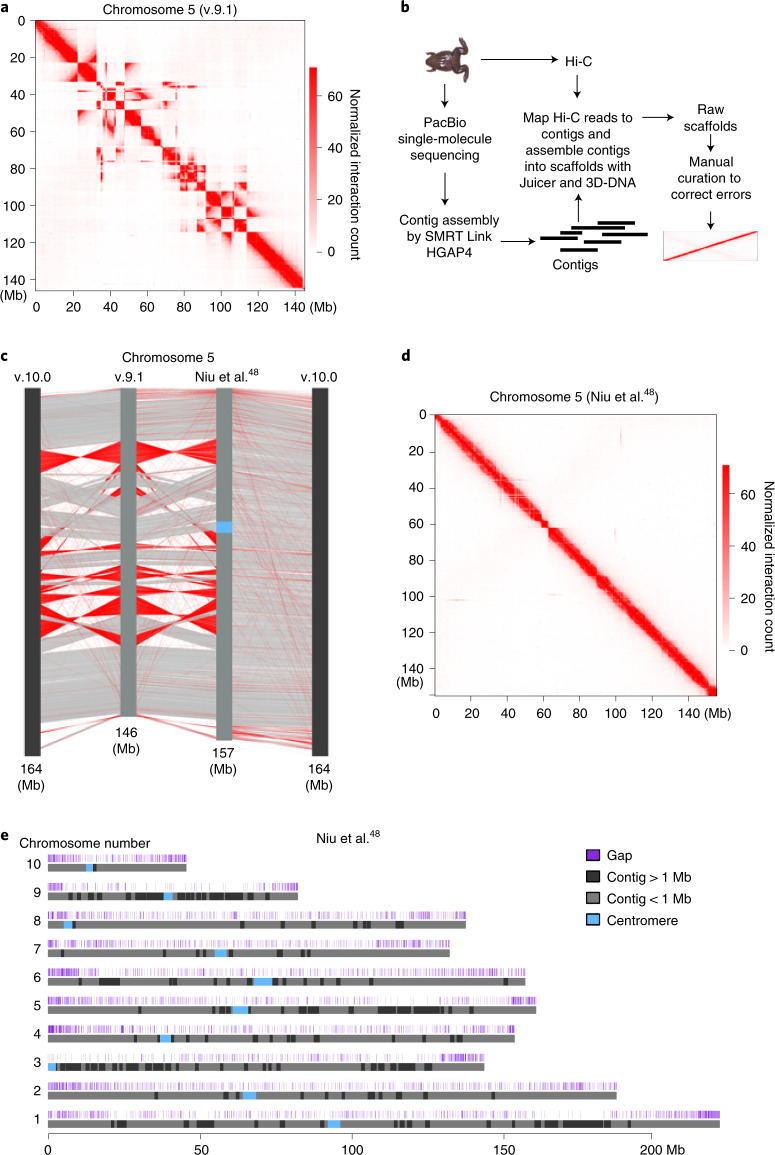
De novo assembly of the reference genome of *X. tropicalis* by using Hi-C and single-molecule sequencing. **a**, Heatmap of chromosome 5 as an example to show assembly errors using the v.9.1 reference genome of *X. tropicalis*. **b**, Procedure of de novo assembly of the reference genome of *X. tropicalis*. **c**, Comparison between the v.9.1, de novo assembled and v.10.0 chromosome 5. The red lines show sequences with the orientation reversed. **d**, Heatmap of chromosome 5 to show that assembly errors are mostly corrected in the new version of the reference genome. **e**, Ideograms of *X. tropicalis* new reference pseudomolecules. The top track shows the positions of gaps (dark blue). Contigs longer than 1 Mb are shown in black and contigs shorter than 1 Mb are shown in light gray. The Hi-C datasets for genome assembly were generated from s9 embryos.

**Extended Data Fig. 1:**
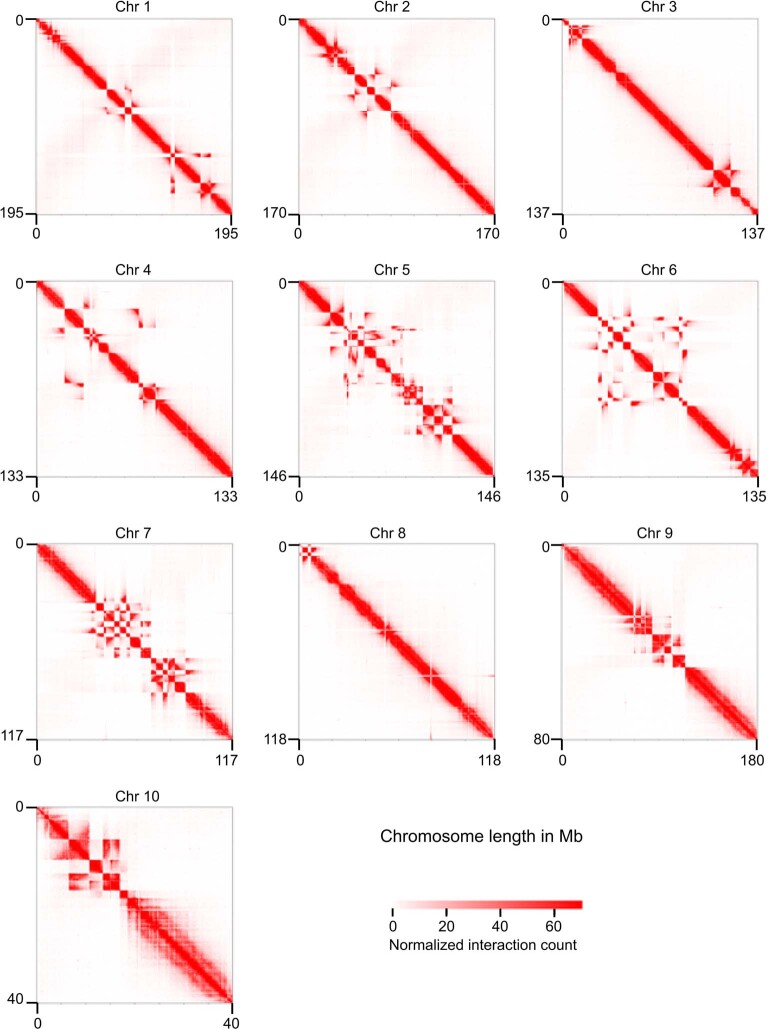
Heatmap of contact frequency for each chromosome. Assembly errors are shown using the v9.1 reference genome of *X. tropicalis for heatmap plotting*. Embryos from stage 9 were used for the Hi-C library preparation for Extended Data Figs. 1 and 2.

**Extended Data Fig. 2:**
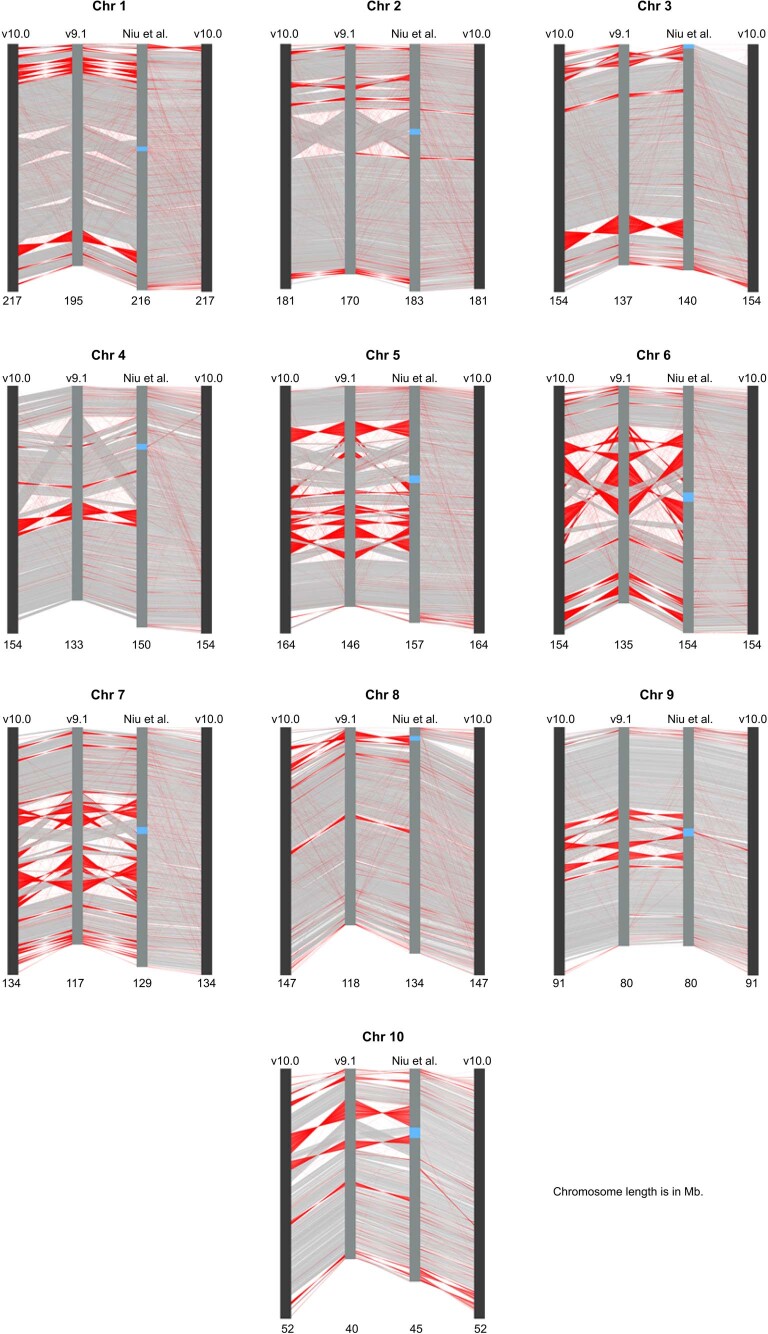
Comparison of v9.1, Niu et al. assembled, and v10.0 genome for each chromosome. Red lines show sequences with orientations reversed.

While carrying out high-throughput chromosome conformation capture (Hi-C) analysis on stage 8 (s8) *X. tropicalis* embryos, we noticed that chromatin interactions plotted at 100-kilobase (kb) resolution using the reference genome v.9.1 showed inversions, misplacements and gaps in nearly every chromosome (Fig. 1a and Extended Data Fig. 1). Thus, to accurately characterize the genome folding patterns in *X. tropicalis*, we conducted a de novo genome assembly of *X. tropicalis* using Hi-C and single-molecule sequencing^42–44^ (Fig. 1b). The newly assembled genome fixed most misplacements, inversions and gaps (Fig. 1c,d, Extended Data Fig. 2 and Supplementary Fig. 1). This new version of the genome was also longer (Supplementary Table 1 and Fig. 1e) and centromere interactions can now be detected (Supplementary Fig. 2). During the preparation of this work, v.10.0 of the *X. tropicalis* genome was released. While both v.10.0 and our assembly fixed major errors, both versions are still flawed with visually identifiable errors (Supplementary Fig. 1; blue and green arrows). A comparison of the three versions is shown in Supplementary Table 1. Conclusions from the following analyses are the same whether we used v.10.0 or our assembled genome.

### TAD structure appears at the onset of MBT

**Fig. 2:**
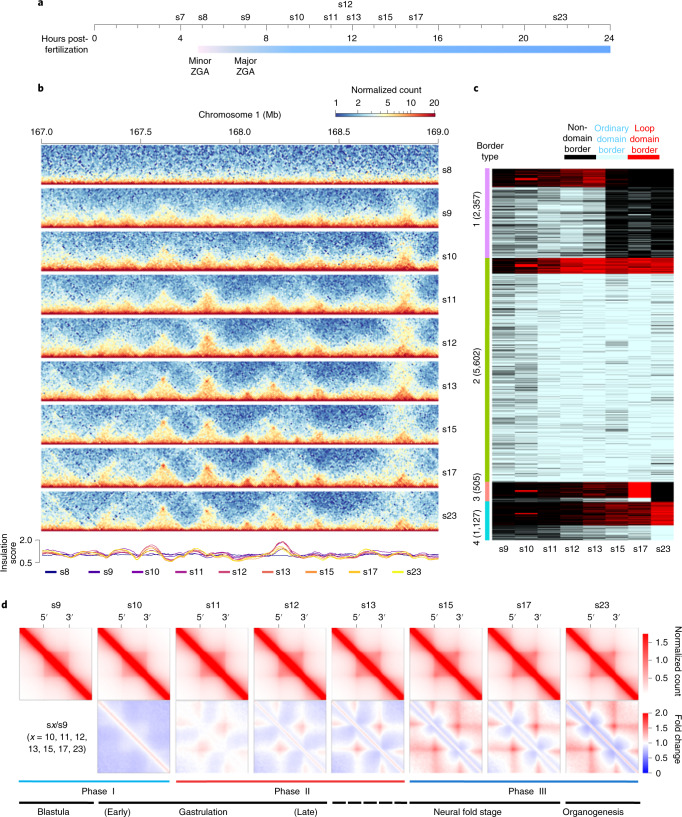
De novo TAD establishment during embryogenesis of *X. tropicalis*. **a**, Schematic representation of the ten developmental stages examined by in situ Hi-C. **b**, Chromatin interaction frequency mapped at a 5-kb resolution. **c**, Clusters of TAD borders appear for ordinary domains (light blue), loop domains (red) and non-domains (black) at each specific developmental stage. ‘Non-domain border’ refers to a genomic region not identified as a TAD border at a specific embryonic developmental stage, which switches to a TAD border at other development stages. **d**, Heatmaps of aggregated TADs for the eight developmental stages. The interaction frequency of aggregated TADs from s10 to s23 was normalized against s9. Three phases of change in the TAD structure are shown below, with the developmental stages also shown (TAD number at s9, s10, s11, s12, s13, s15, s17 and s23: 2,471, 2,805, 3,599, 4,036, 4,160, 3,164, 3,609 and 3,199).

To examine when the 3D chromatin architecture is established in *X. tropicalis*, we generated in situ Hi-C maps on hand-picked s8 embryos (Fig. 2a). A high-resolution (5-kb) inspection of chromatin contact heatmaps failed to reveal any distinct patterns (Fig. 2b), indicating the lack of structural organization before MBT. Next, we determined whether chromatin structures will emerge when rapid synchronized cell division ends by carrying out in situ Hi-C on s9 embryos. Although weak, TAD-like structures appeared across chromatin contact heatmaps (Fig. 2b), suggesting that TAD structures start forming as MBT begins in *X. tropicalis*.

### TAD structure changes continuously during embryo development

**Extended Data Fig. 3:**
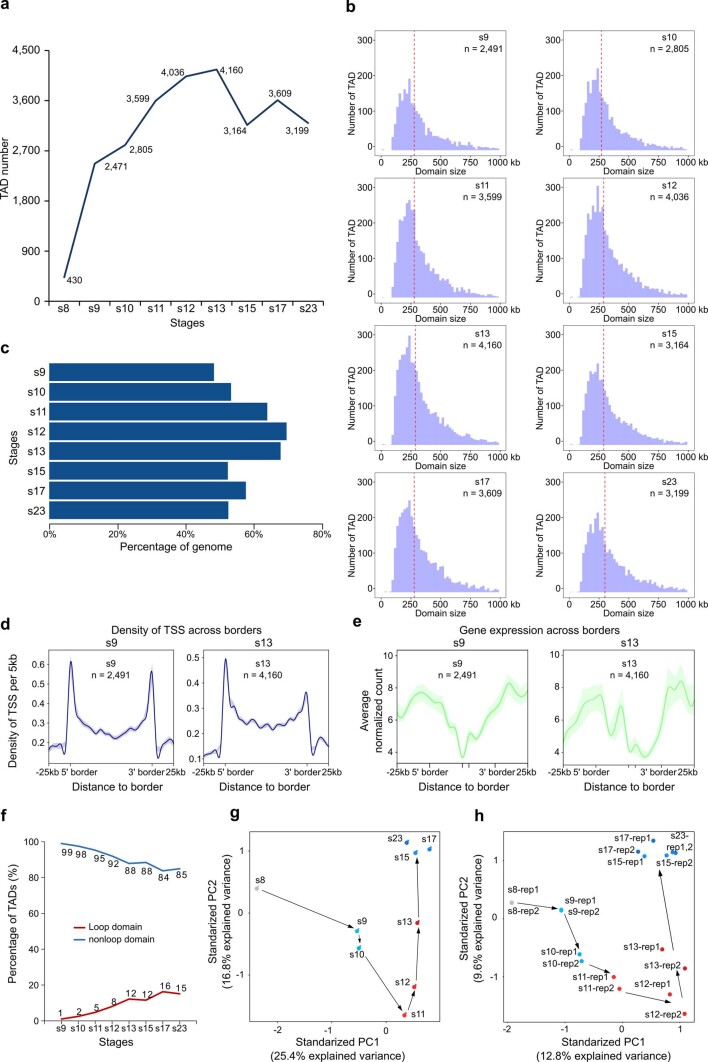
Analysis for TADs identified at different developmental stages. **a**, Number of TADs identified at different developmental stages. **b**, TAD size distribution at different developmental stages. **c**, Percentage of genome folded into TADs at different developmental stages. **d**, Density of TSS across TAD borders at s9 and s13. **e**, Gene expression level across TAD borders at s9 and s13. Data in **d** and **e** are represented as mean±SEM. **f**, Loop and ordinary domains identified at different developmental stages. **g & h**, PCA analysis of insulation scores for Hi-Cs on embryos at different developmental stages.

We continued to examine the changes in chromatin conformation at later developmental stages (stages 10, 11, 12, 13, 15, 17, and 23) after major ZGA (Fig. 2b). TAD boundaries increased progressively from 2,471 at s9 to >3,000 at s11 (Extended Data Fig. 3a,b). This level was maintained throughout the later developmental stages and with relatively stable median TAD sizes (Extended Data Fig. 3a,b). Consistent with this pattern, the percentage of the genome folded into TADs positively correlated with the number of TADs established at each stage (Extended Data Fig. 3c). Overall, TAD borders were stable during development (Fig. 2c) and contained a high level of gene expression (Extended Data Fig. 3d,e).

**Extended Data Fig. 4:**
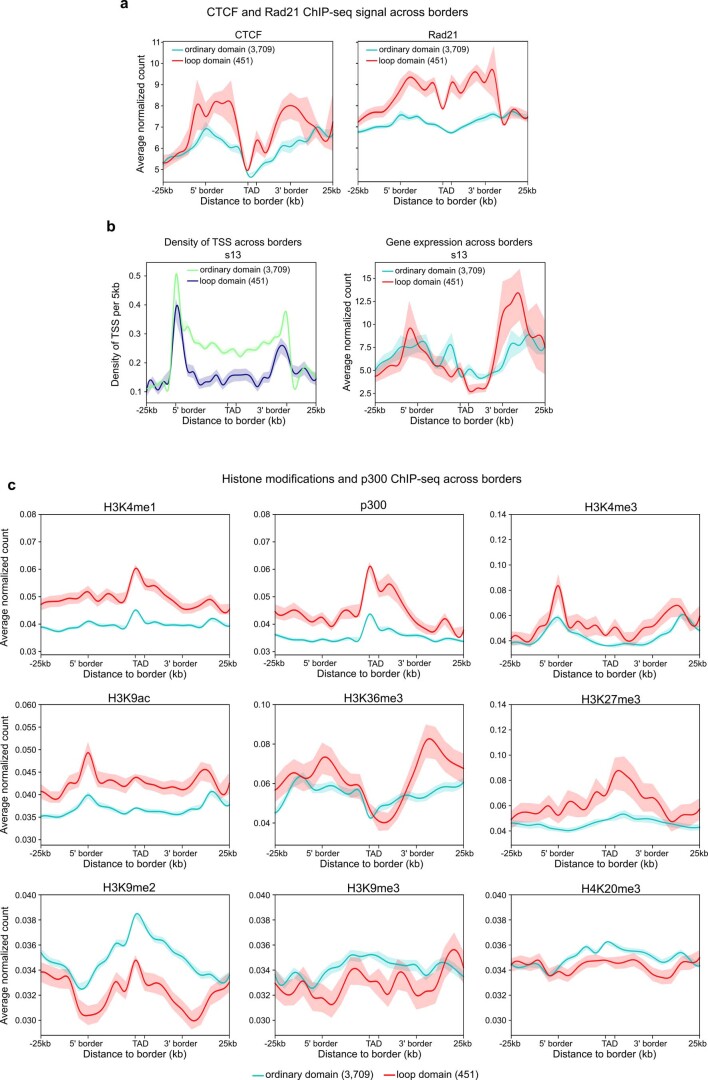
TAD analysis at different developmental stages. **a**, CTCF and Rad21 binding across loop domain and ordinary domain borders. **b**, TSS density and gene expression level across loop domain and ordinary domain borders. **c**, Histone modifications and p300 ChIP-seq signals across loops and ordinary domain borders. All data in this figure are represented as mean±SEM.

To compare the changes in chromatin interaction patterns during embryogenesis, we aligned all domains and calculated the average interaction frequency within and between TADs. For domains at s9 and s10, the interaction loops formed between borders were not apparent, suggesting that the domains at these two stages are mostly ordinary domains whose borders do not form loops (Fig. 2d). Chromatin interaction frequency between borders started appearing at s11 and became increasingly stronger during later developmental stages (Fig. 2d), indicating that loop domains are established later. The percentage of loop domains also increased as embryo development progressed (Extended Data Fig. 3f). Loop domains with borders interacting at high frequency formed mainly between new borders instead of between the preexisting borders of ordinary domains (Fig. 2c). Principal component analysis (PCA) on the directionality index (DI) also reflected changes occurring at several distinct transition points between s8 and s9, s10 and s11 and s13 and s15 in the chromatin interaction pattern of the *X. tropicalis* genome (Extended Data Fig. 3g,h). Compared to ordinary domains, the borders of loop domains have stronger CTCF and Rad21 binding and contain a higher level of gene expression (Extended Data Fig. 4a,b). Loop domain borders are also characterized by higher active histone modifications, such as H3K4me3 and H3K9ac (Extended Data Fig. 4c), while the inside of loop domains is enriched with the repressive histone mark, H3K27me3 (Extended Data Fig. 4c).

We further characterized changes in chromatin interactions by normalizing the chromatin interaction frequency of aggregated TADs against s9. TADs formed at s9 and s10 were similar to each other in chromatin interaction frequency (Fig. 2d). We also observed this for TADs from s11, s12 and s13, as well as TADs formed at s15, s17 and s23 (Fig. 2d). Our results also indicated that interaction loops between TAD borders are continuously consolidated from s11 to s23.

### TADs consolidate as CTCF and Rad21 expression increases

In vertebrate genomes, CTCF motifs at TAD borders are, in general, paired convergently^11,33,34^. In line with previous studies, our analysis also revealed similar convergent CTCF motif orientation for TADs identified at the different stages of development in *X. tropicalis* (Supplementary Fig. 3a). This result suggests that TAD formation in *X. tropicalis* may also require CTCF. To explore this further, we examined changes in protein expression for CTCF and Rad21 by western blot. Low levels of CTCF were detected at s8 and s9 but increased dramatically at s11 (Supplementary Fig. 3b), the stage when loops first appear (Fig. 2d). The Rad21 protein expression pattern was also similar to CTCF (Supplementary Fig. 3b). To reveal if CTCF and Rad21 binding to DNA is correlated with their protein levels, we carried out chromatin immunoprecipitation followed by sequencing (ChIP–seq) analyses of CTCF and Rad21. We found that CTCF and Rad21 bound weakly to DNA at s9 and then increased as development progressed (Supplementary Figs. 4 and 5; normalized to CTCF and Rad21 ChIP spike-in K562 cells). Together, these results indicate that the sequential formation of TADs and loops is correlated with the increase in CTCF and Rad21 protein expression and binding to endogenous genomic loci.

### DI is higher at one side of TAD borders

**Fig. 3:**
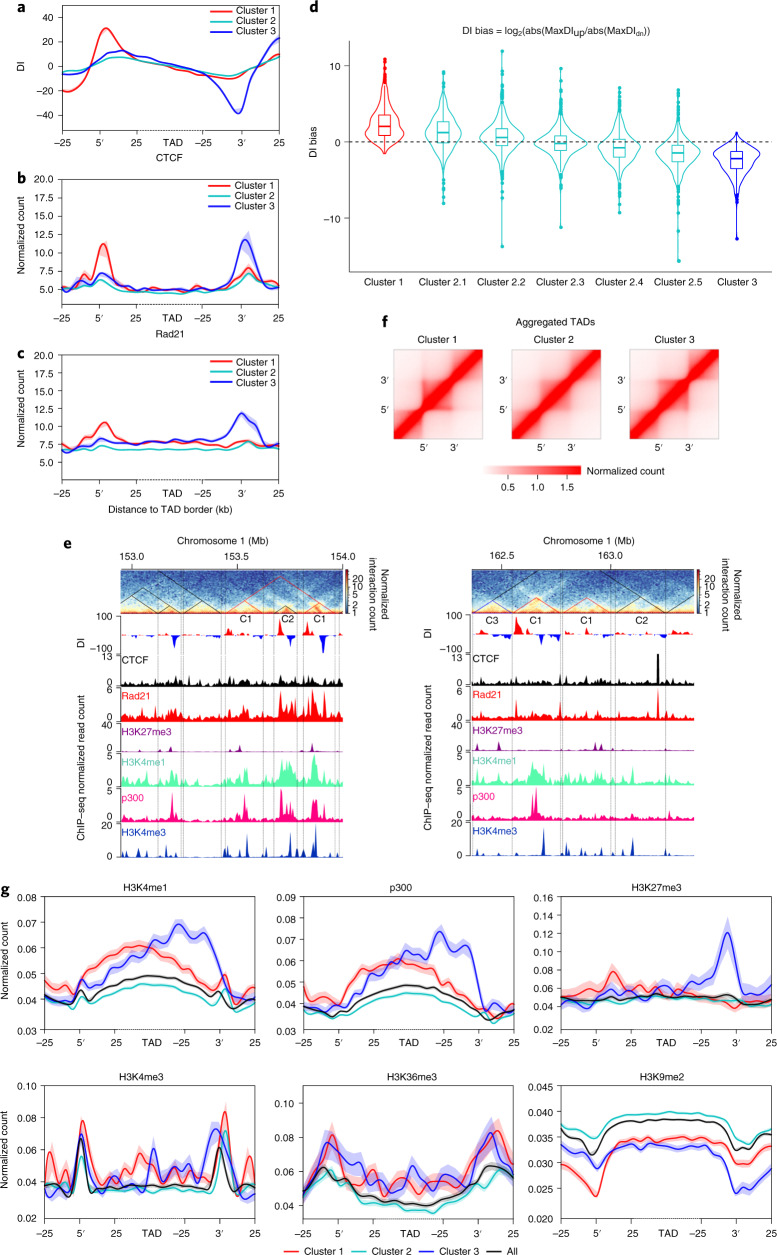
Orientation-biased CTCF and Rad21 enrichment at TAD borders of higher DI values. **a**, DI for three clusters of TADs identified in embryos at s13: 555, 3,191 and 414 TADs in clusters 1, 2 and 3, respectively. **b**, CTCF enrichment is biased to borders with higher DI values. **c**, Rad21 enrichment is biased to borders with higher DI values and drops toward the border on the other side of the TAD. **a**–**c**, Data are presented as the mean ± s.e.m. **d**, DI for TADs of cluster 1, five subclusters of clusters 2 and cluster 3. According to the ranking of DI strength, TADs in cluster 2 were divided into five subclusters. Data are presented as box plots within violin plots. The minima, maxima, center and bounds of each box plot refers to quartile 1–1.5 interquartile range, quartile 3 + 1.5 interquartile range, median, first and third quartiles of the data. **e**, Examples of TADs for the three different clusters. **f**, Aggregated heatmaps for three clusters of TADs. **g**, Histone modifications and p300 enrichment patterns across the borders of three clusters of TADs. Data are presented as the mean ± s.e.m.

**Extended Data Fig. 5:**
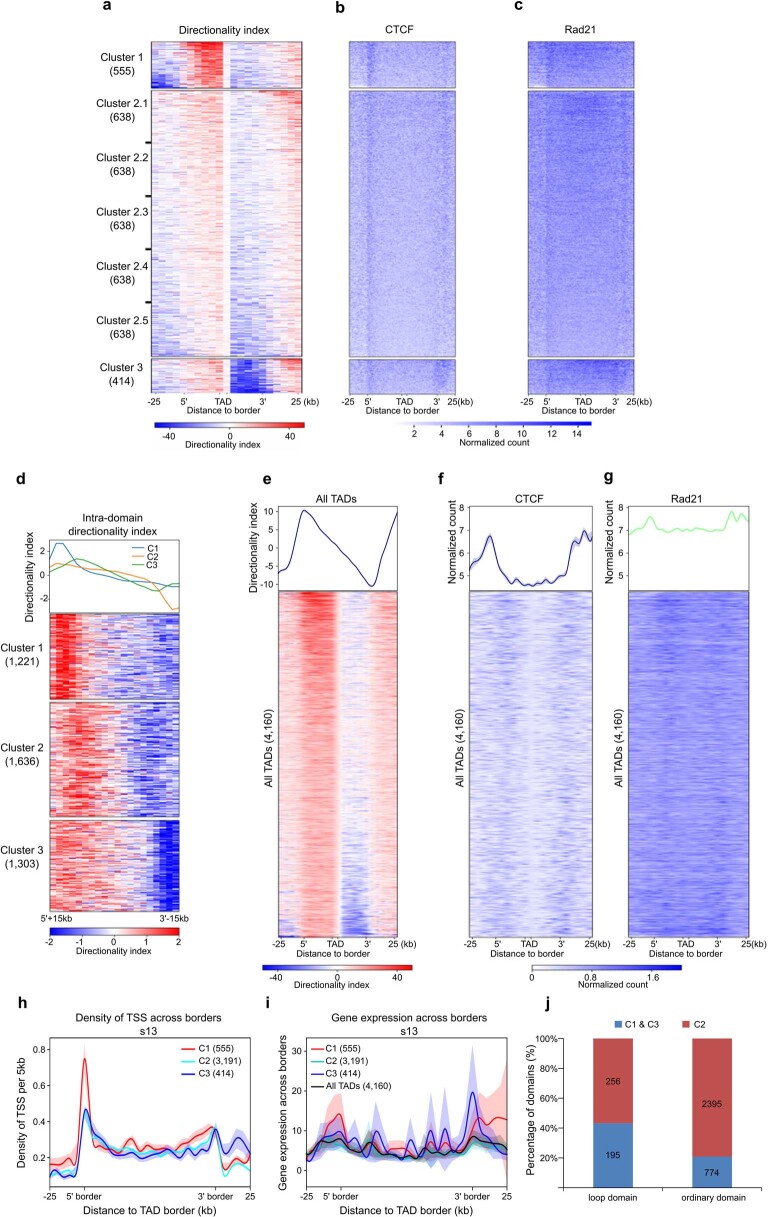
Orientation-biased CTCF and Rad21 enrichment at TAD borders of higher directionality index values. **a**, TADs of stage 13 embryos clustered on directionality index. Cluster 2 is further divided into five sub-clusters with an equal number of TADs. **b**, CTCF ChIP-seq signal across TAD borders in each cluster. **c**, Rad21 ChIP-seq signal across TAD borders in each cluster. **d**, Intra-domain directionality index was calculated and was used to cluster TADs into three groups. There are 1,221, 1,636 and 1,303 TADs in clusters 1, 2 and 3, respectively. Note the low DI values on the y-axis. **e**, Average directionality index across borders of all TADs without clustering. TADs are arranged with DI of decreasing absolute value on the 5′ border and DI of increasing absolute value on the 3′ border. **f**, Relative enrichment of CTCF across borders of all TADs showing indistinguishable differences in signal strength at borders on both sides of TADs. **g**, Relative enrichment of Rad21 across borders of all TAD showing indistinguishable differences in signal strength at borders on both sides of TADs. Data in **e**, **f**, and **g** are represented as mean±SEM. **h**, Density of TSS across three clusters of TAD borders. **i**, RNA level across the borders of three clusters of TADs. Data in **h** and **i** are represented as mean±SEM. **j**, Percentage of loops and ordinary domains in clusters 1&3 and cluster 2.

For a DNA fragment, the preference of upstream or downstream chromatin interaction can be measured as the DI^4,45^. To explore the underlying cause of directionality, we aligned TADs at the 5′ and 3′ borders and extended 5 bins (10 kb per bin) upstream and downstream of the two borders. We then clustered TADs based on the DIs of each domain at the two borders. Surprisingly, we found that TADs from s13 can be grouped into three distinct clusters (Fig. 3a and Extended Data Fig. 5a). The absolute DI values upstream and downstream of the borders in clusters 1 and 3 were strikingly higher at one side of the border (Fig. 3a), whereas the values in cluster 2 were similar at both borders (Fig. 3a and Extended Data Fig. 5a). We also observed similar enrichment patterns for CTCF and Rad21 binding across the three clusters (Fig. 3b,c and Extended Data Fig. 5b,c). When we further divided TADs in cluster 2 into five subclusters of an equal number of TADs (Extended Data Fig. 5a), we found that the absolute DI values were also higher at either the 5′ or the 3′ border (Fig. 3d). DI bias also exists inside TADs but it is much weaker (Extended Data Fig. 5d). Examples of TADs for the different types of clusters are shown in Fig. 3e and Supplementary Fig. 6. Aggregating the three clusters of domains showed a stripe-like structure in the domains of clusters 1 and 3 (Fig. 3f). These results together suggest that the difference in DI patterns across borders could be due to orientation- and enrichment-biased binding of both CTCF and cohesin at one side of the TAD border.

The unexpected pattern of DI bias at TAD borders suggests that simply aggregating all TADs before the analysis concealed rich structural information. Indeed, the aggregation of all TADs showed indistinguishable patterns of DI signals and CTCF and Rad21 binding at borders (Extended Data Fig. 5e–g). Similarly, the enrichment patterns of H3K4me1, p300, H3K4me3, H3K36me3, H3K27me3 and H3K9me2 at TAD borders were different for each cluster (Fig. 3g). Thus, chromatin states may affect the process of cohesin-mediated extrusion similar to previously reported asymmetric TAD architecture formation^46,47^. When we examined RNA expression, we found it was enriched at the borders of the cluster 1 and 3 domains with a bias toward the higher DI side, even though gene density was not obviously different (Extended Data Fig. 5h,i). At s13, >40% of loop domains and <20% of ordinary domains were in clusters 1 and 3 (Extended Data Fig. 5j). Together with the loop domain analysis (Extended Data Fig. 4), these results suggest that loop domains formed later are more involved in active transcription.

We carried out similar DI pattern analyses using previously published data for human K562 (ref. ^11^) and *Drosophila* S2 cells^48^. Strikingly, we found even more obvious patterns in human K562 for all the three parameters analyzed (Supplementary Fig. 7a–c); even the five subclusters of cluster 2 showed bias in DI, CTCF and Rad21 binding (Supplementary Fig. 7d–f). Although DI bias was also found for TADs in S2 cells (Supplementary Fig. 8a,b), there was no CTCF enrichment bias for any of the three clusters (Supplementary Fig. 8c), which is consistent with the lack of convergent CTCF motifs at TAD borders in the *Drosophila* genome^49^. Taken together, these results imply that the two borders of a TAD can be different in multiple aspects and highlight the internal heterogeneity of TADs from one end to the other.

### Effects of transcription inhibition on TAD establishment

**Fig. 4:**
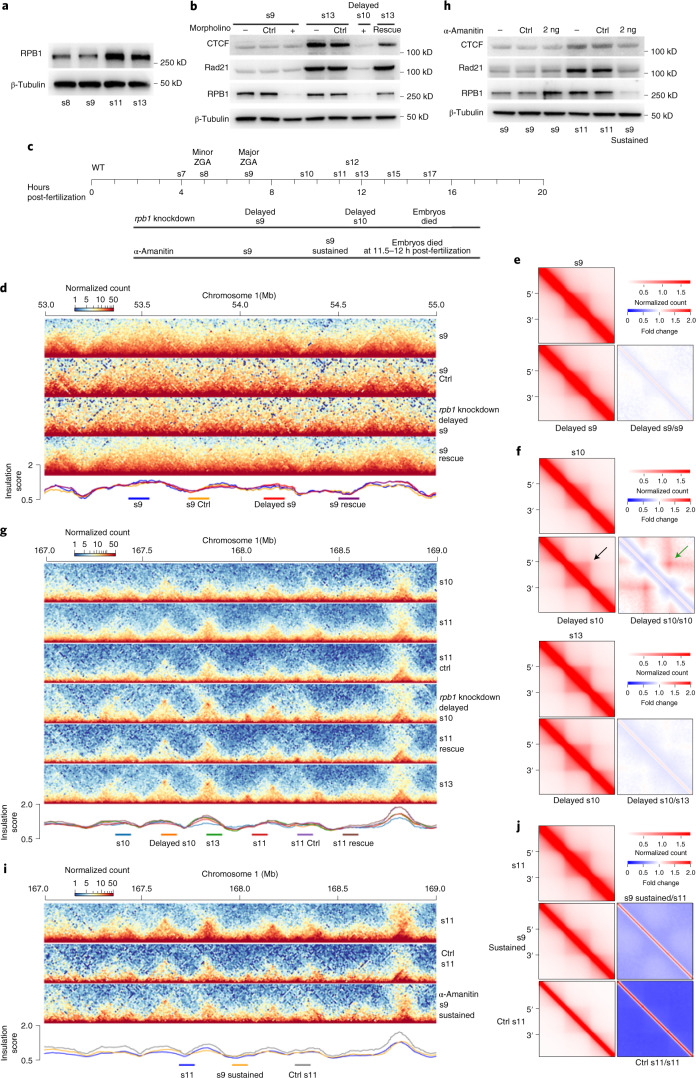
De novo TAD establishment is independent of transcription. **a**, Western blot of RPB1 in WT embryos at the four developmental stages. **b**, Western blot of proteins in embryos with RPB1 knocked down by morpholinos and in embryos that were rescued. Note that the CTCF and Rad21 protein levels at delayed s10 were similar to s9 WT. Morpholino control (Ctrl); no morpholino (−); rpb1 morpholino (+); rpb1 rescue. See also Supplementary Table 4 for the rpb1 coding sequence for the rescue experiment. **c**, Schematic representation of the embryogenesis process arrested by RPB1 knockdown and transcription inhibition by α-amanitin. **d**, Example of a region showing the RPB1 knockdown effects on TAD structure at s9. **e**,**f**, Aggregated and normalized heatmaps for s9 and delayed s9 (**e**), and s10, s13 and delayed s10 (**f**) for the RPB1 knockdown experiment. **g**, Example of a region showing the RPB1 knockdown and rescue effects on TAD structure at s11. **h**, Western blot of proteins in embryos inhibited with α-amanitin. Note that the CTCF and Rad21 protein levels at s9 sustained were similar to s9 WT. Water as control (Ctrl); no α-amanitin (−); the amount of α-amanitin injected was 2 ng per embryo. All western blot experiments in this figure were repeated at least twice unless otherwise stated. **i**, Example of a region showing the effects of α-amanitin inhibition on TAD structure. **j**, Aggregated and normalized TAD analysis for embryos of WT s11 and α-amanitin-inhibited s9 sustained. Source data

**Extended Data Fig. 6:**
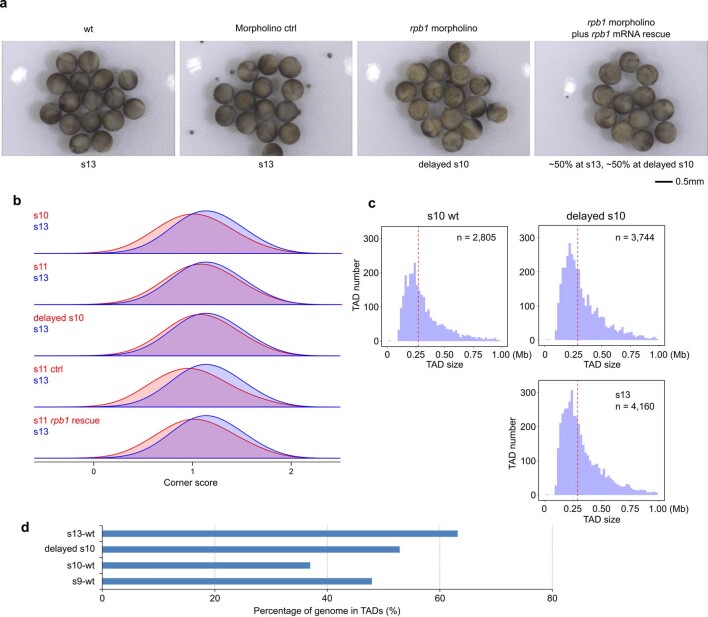
Effects of rpb1 knock-down on TAD establishment. **a**, Effect of *rpb1* knock-down on embryo development. Knock-down of *rpb1* was repeated for at least two times with similar results. **b**, Arrowhead corner score distribution. **c**, TAD size distribution. **d**, Percentage of genome folds in TADs.

**Extended Data Fig. 7:**
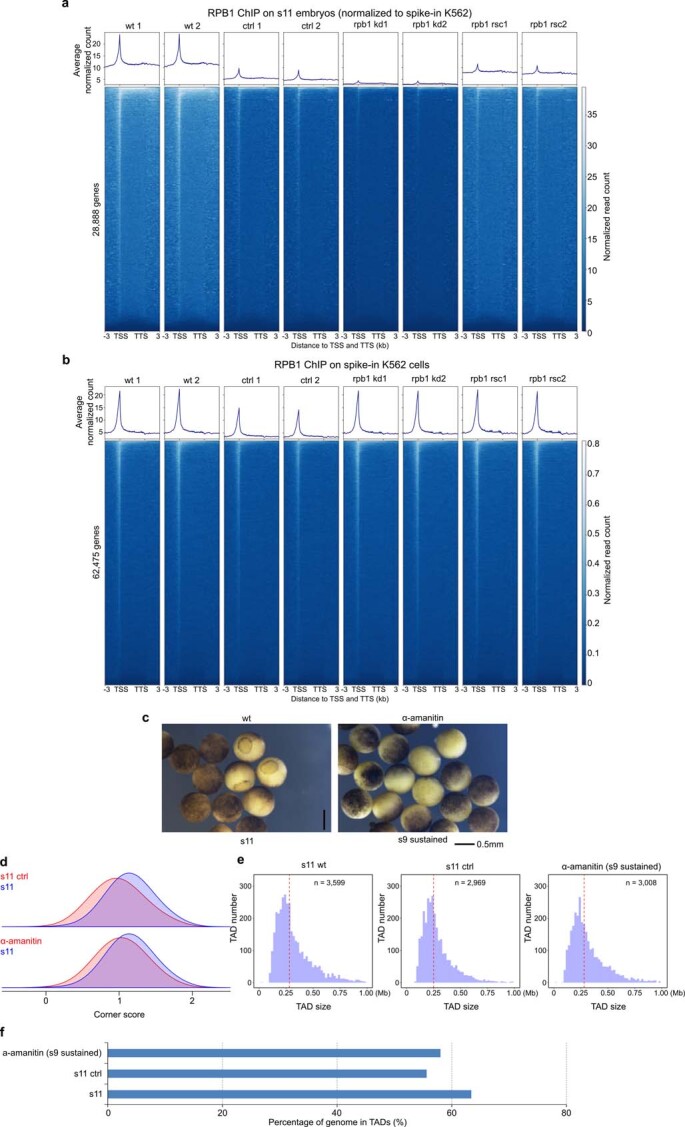
Effects of transcription inhibition by α-amanitin and *rpb1* knock-down. **a**, RPB1 ChIP-seq signal across genes normalized to spike-in K562. **b**, RPB1 ChIP-seq signal across human genes in spike-in K562 cells. For **a** & **b**, Wild type (wt), morpholino control (ctrl), *rpb1* morpholino knock-down (rpb1 kd), *rpb1* rescue (rpb1 rsc), all experiments were conducted in two biological replicates. **c**, Effect of transcription inhibition on embryo development. Transcription inhibition with α-amanitin was repeated for at least two times with similar results. **d**, Arrowhead corner score distribution. **e**, TAD size distribution. **f**, Percentage of genome folds in TADs.

A recent study by Chen and colleagues^32^ showed transcription is important for TAD establishment in human embryogenesis. To determine whether this process is also important for TAD establishment during *X. tropicalis* embryogenesis, we inhibited RNA Pol II activity with morpholinos targeting the DNA-directed RNA polymerase II subunit RPB1 (RPB1) protein, a critical component of RNA Pol II. Morpholinos against RPB1 efficiently reduced protein levels in embryos developed to s10 (Fig. 4a,b). RPB1 knockdown dramatically delayed embryo development and caused embryos to die before reaching s11 (Fig. 4c and Extended Data Fig. 6a). However, TADs still formed in delayed s9 embryos even after RPB1 translation was inhibited (Fig. 4d) and had no observable interactions between TAD borders (Fig. 4e). In contrast to WT embryos, loop interactions between TAD borders began appearing in delayed s10 embryos (Fig. 4f; black and green arrows). Notably, RPB1 depletion alone appeared insufficient in disrupting the establishment of TAD structures in delayed s10 embryos (Fig. 4f,g and Extended Data Fig. 6b–d), even though both RPB1 binding and gene expression levels were reduced (Extended Data Fig. 7a,b and Supplementary Fig. 9a). In fact, further examination showed that despite reduced RPB1 binding (Extended Data Fig. 7a), RPB2, the second-largest RNA Pol II subunit, was still bound across the gene body (Supplementary Fig. 10), possibly by forming a subassembly with other components of the transcription machinery^50,51^. Nevertheless, compared to WT embryos, the above observations may not be that surprising if we consider that the delayed s10 embryos are only blocked developmentally but not in the formation of 3D genome architecture.

We also inhibited transcription by injecting α-amanitin into embryos (Fig. 4h and Supplementary Fig. 9b). Consistent with the effects of morpholinos, α-amanitin treatment also delayed and aborted embryo development, resulting in embryos dying around s11 (Fig. 4c and Extended Data Fig. 7c) but without affecting the formation of TAD structures (Fig. 4i,j and Extended Data Fig. 7d–f). Together, these results show that the de novo establishment of TADs in *X. tropicalis* does not seem to be stringently dependent on transcription, which is similar to fruit flies and mice^18–20^ but distinct from human embryogenesis^32^.

### Requirement of CTCF and Rad21 for TAD establishment

**Fig. 5:**
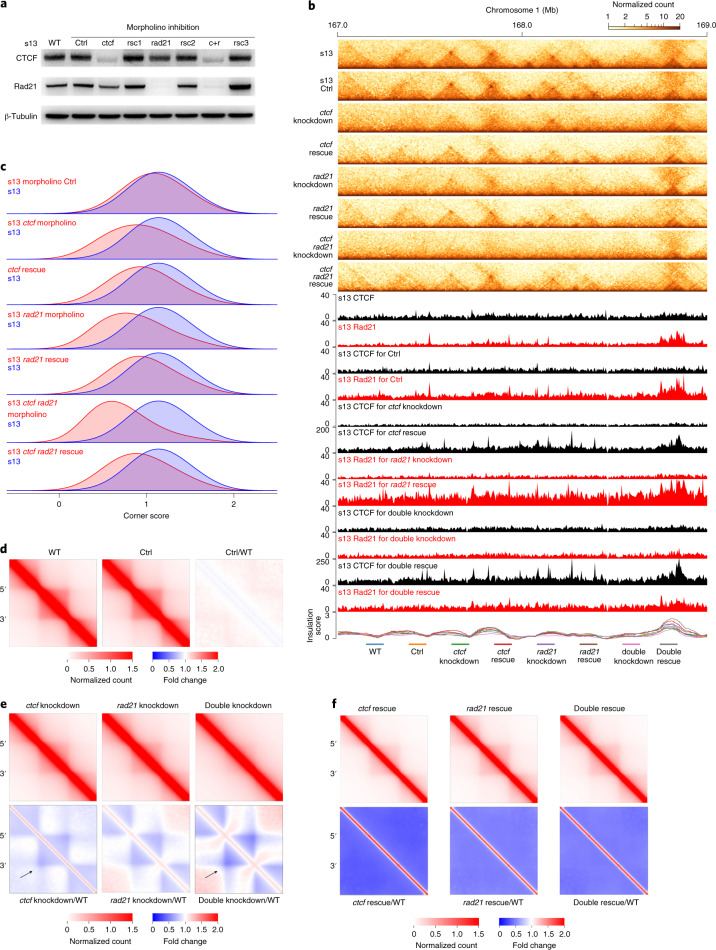
Requirement of CTCF and Rad21 for TAD establishment in *X. tropicalis* embryos. **a**, Western blot of CTCF and Rad21 knockdown by morpholinos in embryos at s13. WT, morpholino control (Ctrl), CTCF morpholino (ctcf), Rad21 morpholino (rad21), ctcf rescue (rsc1), rad21 rescue (rsc2), CTCF and Rad21 morpholinos (c+r) and double rescue of ctcf and rad21 (rsc3) are shown. See Supplementary Table 4 for the gene coding sequences for rescue. The western blot experiments in this figure were repeated at least twice with similar results. **b**, Example region showing the knockdown and rescue effect on TAD structures. *y* axis: normalized ChIP–seq read count. **c**, Arrowhead corner score distribution for WT, morpholino Ctrl and knockdowns with CTCF morpholino, Rad21 morpholino, combined CTCF and Rad21 morpholinos and rescued. All experiments were carried out on s13 embryos. Higher corner score values on the *x* axis indicate a greater likelihood of being at the corner of a domain; the height of the curve indicates the density of corner score values within a specific range. **d**, Heatmaps of aggregated TADs in WT and Ctrl embryos. **e**, Heatmaps of aggregated TADs in CTCF and Rad21 knockdown embryos. f, Heatmaps of aggregated TADs in CTCF and Rad21 expression–rescued embryos. In **d**–**f**, The black arrows point to interacting borders. The interaction frequency of aggregated TADs was normalized against WT s13. Source data

CTCF is critical for TAD formation during human embryogenesis^32^. We speculated that both CTCF and Rad21 might also be required for TAD establishment during *X. tropicalis* embryogenesis. We tested this hypothesis by depleting CTCF and Rad21 with morpholinos individually or in combination (Fig. 5a). The reduction in CTCF or Rad21 expression decreased CTCF and Rad21 binding across the genome (Fig. 5a and Supplementary Figs. 11 and 12; normalized to spike-in K562 cells) and weakened TAD structures (Fig. 5b and Supplementary Fig. 13a). Overexpression of either CTCF, Rad21 or both factors rescued these changes (Fig. 5b and Supplementary Figs. 11–13). The arrowhead corner scores (a score indicating the likelihood that a pixel in the heatmap is at the corner of a contact domain^11^) for TADs were also reduced after the knockdown of the two factors (Fig. 5c). Insulation at most borders was also weakened (Supplementary Fig. 13b). TAD structures were almost completely abolished when both CTCF and Rad21 were depleted (Fig. 5b). Knockdown of CTCF, Rad21 or both factors reduced the number of TADs but not their median size (Supplementary Fig. 13c). The percentage of the genome folded into TADs was still proportional to the number of TADs (Supplementary Fig. 13d). Finally, embryos with either CTCF or Rad21 knockdown survived at least through the neural folding stage and appeared normal at s13 (Supplementary Fig. 14). Together, these results support that both CTCF and Rad21 are required for the de novo establishment of TADs during *X. tropicalis* embryogenesis.

The knockdown of CTCF not only compromised overall TAD formation but also weakened the interactions between TAD borders (Fig. 5d,e). In contrast, knockdown of Rad21 weakened more interactions within the TAD (Fig. 5d,e). The combined knockdown of CTCF and Rad21 abolished both TADs and loops forming between TAD borders (Fig. 5e; black arrows). These structures were rescued with the expression of either CTCF or Rad21 or both proteins (Fig. 5f). Taken together, these results indicate that CTCF appears to contribute more to loop formation^52,53^, while cohesin Rad21 seems to have more influence on intra-domain interaction.

### Chromatin remodeling is required for de novo TAD formation

**Fig. 6:**
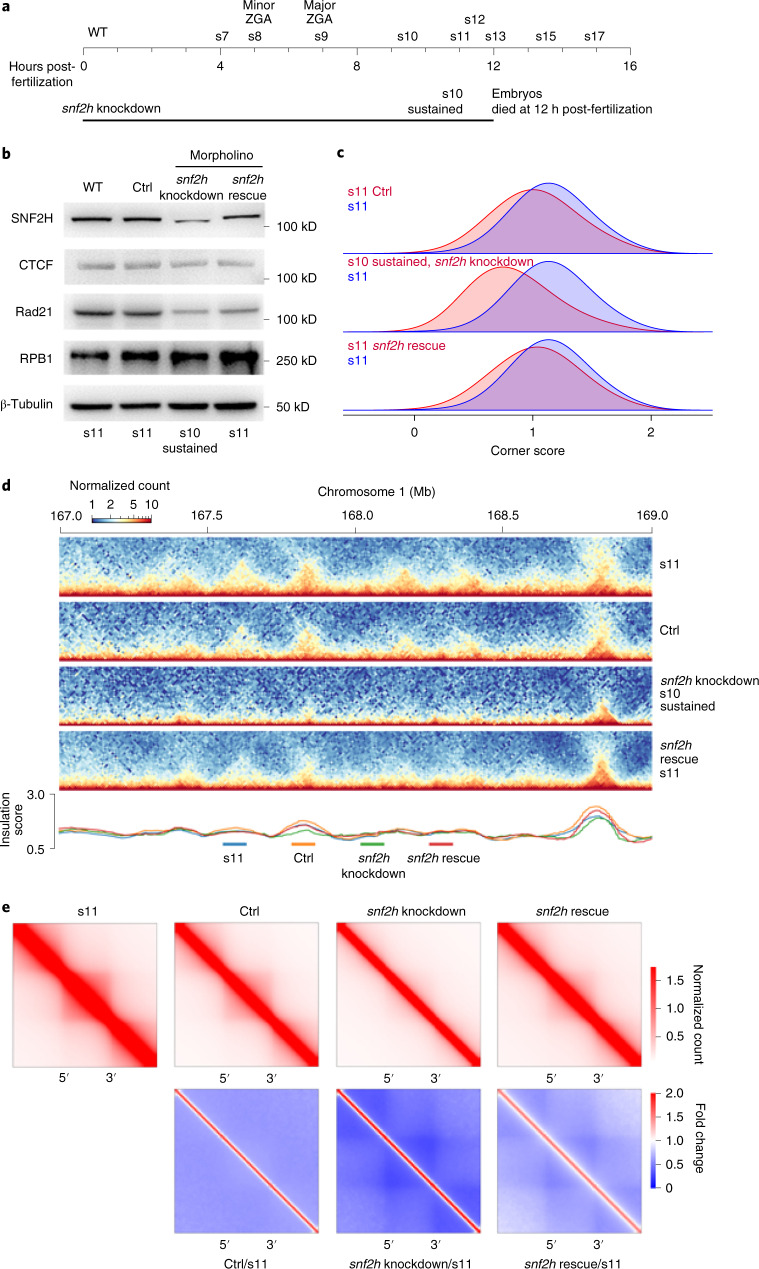
Chromatin remodeling is required for TAD establishment and embryo development. **a**, Schematic representation of the embryogenesis process arrested by the knockdown of SNF2H, the ATPase component of the ISWI complex. **b**, Western blot of proteins in embryos with SNF2H knocked down by morpholinos and in embryos that were rescued. WT, morpholino control (Ctrl), SNF2H morpholino (snf2h knockdown), SNF2H rescue (snf2h rescue). The western blot experiments in this figure were repeated at least twice with similar results. **c**, The domain arrowhead corner score distribution of *snf2h* knockdown embryos. Morpholino control (Ctrl). **d**, Example region to show *snf2h* knockdown and rescue effect on TAD establishment. **e**, Heatmaps of aggregated TADs normalized against s11. Source data

The accessibility of DNA for protein binding is regulated by chromatin remodeling complexes such as ISWI, which was recently shown to mediate CTCF binding in mammalian cells^54^. Therefore, we speculated that ISWI might affect the establishment of TAD structures during early embryogenesis through mediating CTCF binding also. To test this hypothesis, we knocked down sucrose nonfermenting protein 2 homolog (SNF2H) (Fig. 6a,b), the ATPase subunit of the ISWI complex. SNF2H depletion compromised CTCF binding to the genome but this was partially rescued (Supplementary Fig. 15; normalized to spike-in K562). TAD structures were also severely weakened and could also be partially rescued (Fig. 6c–e and Supplementary Fig. 16a,b). Similar to RNA Pol II, the reduction of SNF2H arrested embryo development at s11 before embryos died (Fig. 6a). Approximately 50% of embryos were partially rescued (Supplementary Fig. 16c). Overall, these results suggest that chromatin remodeling plays an essential role in establishing TAD structures, possibly through mediating CTCF binding.

### Progressive genome compartmentalization after ZGA

**Fig. 7:**
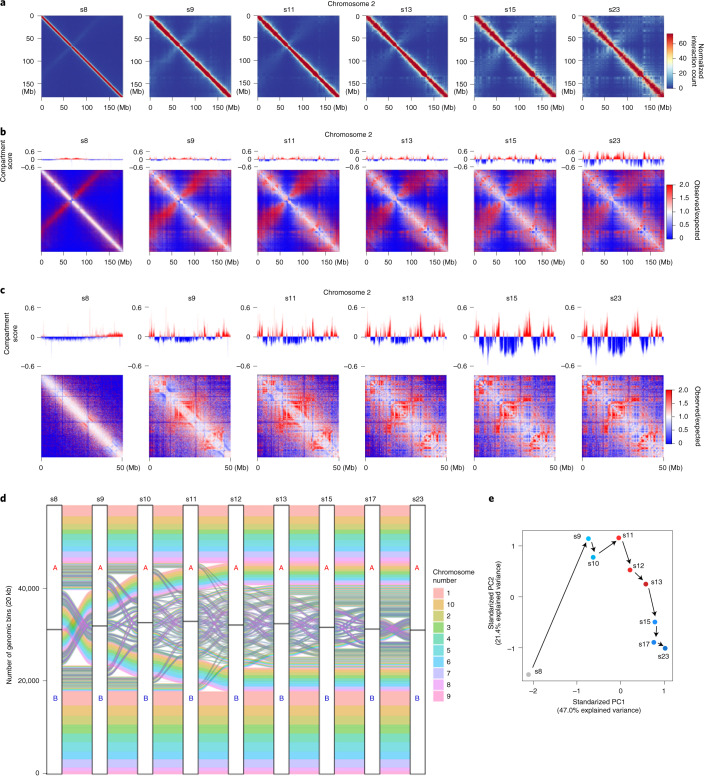
Continuous compartmentalization during embryogenesis. **a**, Heatmaps of chromosome 2 plotted at a 50-kb resolution at multiple developmental stages. **b**, Hi-C matrices (observed/expected) for chromosome 2 at a 50-kb resolution at multiple developmental stages. **c**, Hi-C matrices (observed/expected) of an example region between 0 and 50 Mb in chromosome 2. **d**, Chromatin switched between A and B compartments during embryo development. **e**, PCA of compartment scores derived from adjusted Cscore values of Hi-C matrices at multiple developmental stages.

**Extended Data Fig. 8:**
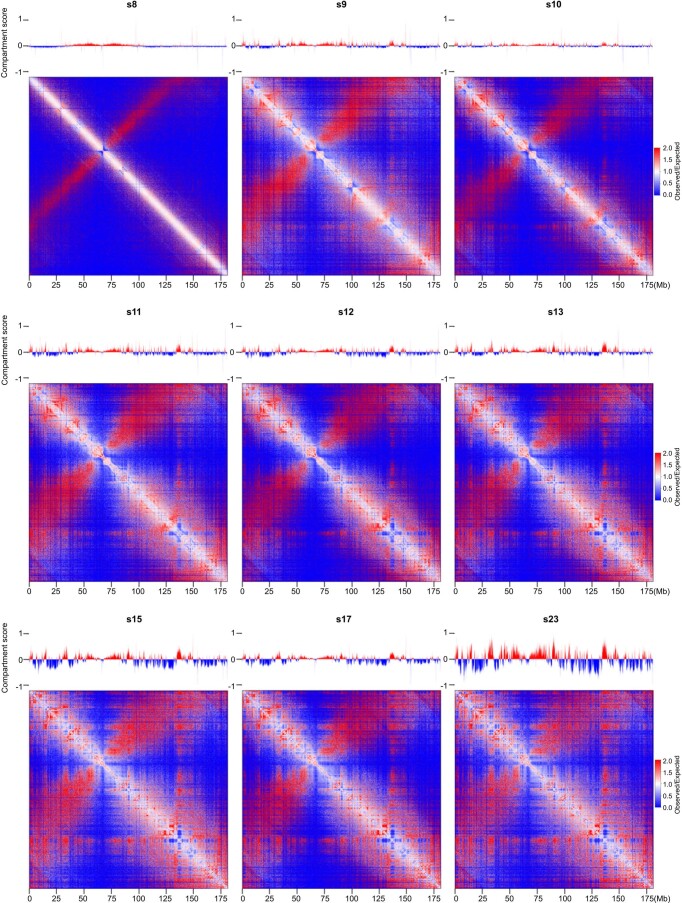
Compartment score for the assignment of compartments A and B. Chromosome 2 is shown as example.

**Extended Data Fig. 9:**
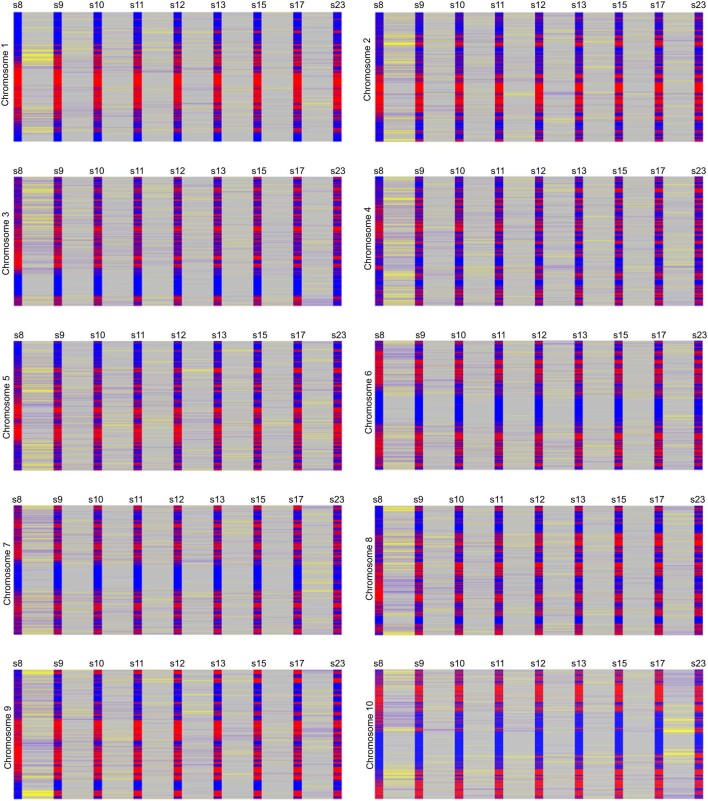
Chromatin switches between compartments A and B. Compartment switches are shown for each chromosome through multiple embryo developmental stages. Red and blue colors show chromatin in compartment A and B, respectively. Grey, yellow and purple lines show no switch, B to A, and A to B switch, respectively.

Separation of chromatin into the active and repressive compartments A and B is another prominent structural feature of animal genomes^7^. We examined compartmentalization by plotting chromatin contact heatmaps at 100-kb resolution. We further computed the compartment score by adjusting the original Cscore with histone modification (Extended Data Fig. 8). Visual inspection of heatmaps revealed continuous expansion of long-range chromatin interactions from s8 to s23 with the appearance of compartment-like patterns starting as early as s13 (Fig. 7a and Supplementary Fig. 17). A zoomed-in view of two genomic regions on chromosome 2 revealed a more obvious initiation of compartmentalization beginning at s9 (Fig. 7b,c). Newly segregated compartments continuously emerged and switched before stabilizing through the later developmental stages (Fig. 7d and Extended Data Fig. 9). PCA analysis of the compartment score derived from adjusted Cscore^55^ also showed that compartmentalization of the genome continually changes through development (Fig. 7e). Together, these results reveal that compartments are continually refined after ZGA initiation and change progressively into new states that are increasingly stable.

### Strength of TADs and compartments varies in adult tissues

**Fig. 8:**
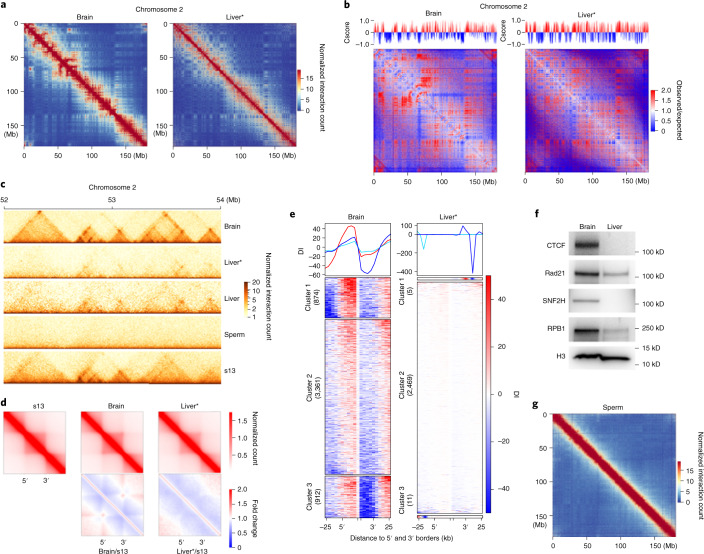
Tissue-specific genome architecture in mature brain, liver and sperm cells. **a**, Heatmaps of chromosome 2 plotted at a 50-kb resolution for brain and liver cells. **b**, Hi-C matrices (oberved/expected) and Cscore for chromosome 2 at 50-kb resolution for brain and liver cells. **c**, Heatmaps of an example region in chromosome 2 showing the gain and loss of TAD structures in the brain, liver and sperm cells compared to s13. The single asterisk next to Liver indicates repeated Hi-C on liver cells with K562 as the spike-in control. **d**, Aggregated TADs of brain, liver and s13 embryo cells and normalization of aggregated TADs against s13 embryos. **e**, DI cluster for TADs of brain and liver cells. In brain cells, we identified 874, 3,361 and 912 TADs in clusters 1, 2 and 3, respectively. In liver cells, we identified 11, 2,469 and 5 TADs in clusters 1, 2 and 3, respectively. **f**, Western blot of CTCF, Rad21, SNF2H, RPB1 and histone H3 as a control in brain and liver cells. The western blot experiments in this figure were repeated at least twice with similar results. **g**, Heatmap of chromosome 2 plotted at a 50-kb resolution for mature sperm cells. Source data

**Extended Data Fig. 10:**
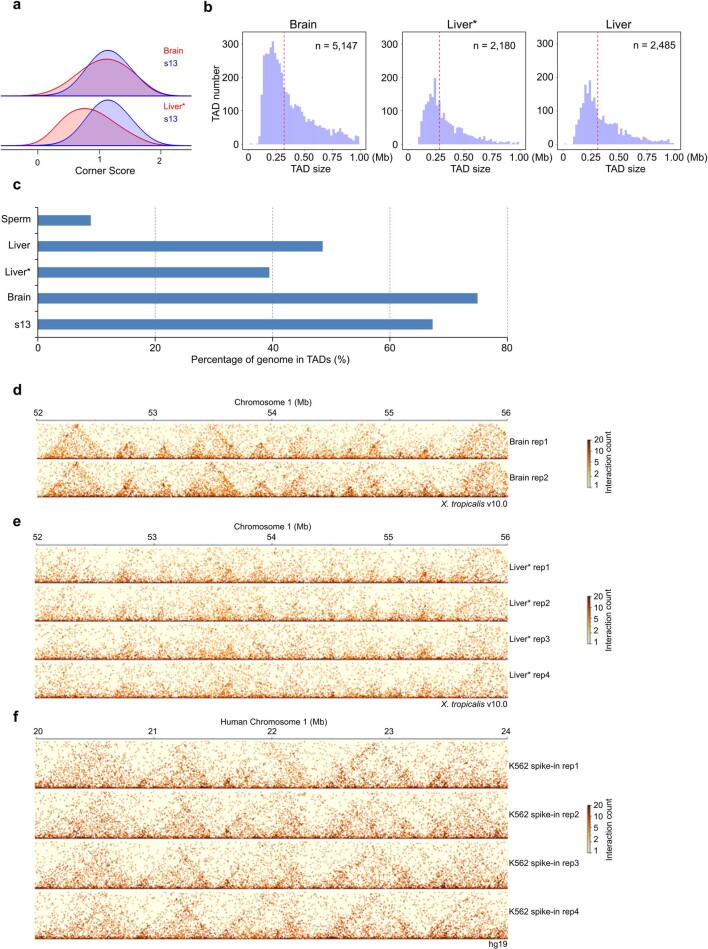
TAD structure in terminally differentiated brain and liver tissues. **a**, Comparison of arrowhead corner score distribution for s13 embryos, brain, and liver tissues. **b**, Number of TADs and size distribution. **c**, The percentage of genome folded into TADs in brain and liver tissues. **d-f**, Example regions to show TADs structure in biological replicate Hi-Cs for brain, liver, and spike-in K562 cells. For each Hi-C replicate, 8 million paired reads from the genome-wide interactions were randomly selected and used for the heatmap plotting. * indicates Hi-C on liver tissue with human K562 as spike-in control.

Whether chromosome architecture is conserved in different tissues of *X. tropicalis* is unknown. To address this issue, we carried out Hi-C on adult brain and liver tissues of *X. tropicalis*. A comparison of chromatin interaction heatmaps for chromosome 2 shows apparent differences in interaction patterns between brain and liver (Fig. 8a). Analysis of Cscore and Hi-C matrices further supports that compartmentalization of chromosomes is distinct between these two tissues (Fig. 8b). Overall, we identified 5,147 and 2,180 TADs in the brain and liver, respectively (Extended Data Fig. 10a–c). Compared to s13 embryos, TAD structures are more evident in brain cells and much weaker in liver cells (Fig. 8c and Extended Data Fig. 10d,f). Also, the distribution of arrowhead corner scores for brain cells is consistently higher than in liver cells (Extended Data Fig. 10a). Aggregation of TADs shows that loops between TAD borders are more frequently formed in brain cells, which was also confirmed by normalizing brain and liver aggregated TADs against those from s13 embryos (Fig. 8d). DI clustering showed similar biases in the strength of chromatin interaction directionality at the borders of TADs in brain cells (Fig. 8e). In western blot analysis, CTCF and SNF2H are highly expressed in brain cells but barely detectable in liver cells, whereas Rad21 and RPB1 are expressed at a lower level in the liver (Fig. 8f). Given that CTCF, Rad21 and SNF2H proteins are required for TAD formation during *X. tropicalis* embryogenesis, the low expression of these factors in adult liver cells may explain the weak TAD structures.

We also examined the genome architecture of mature sperm cells from *X. tropicalis*. Compared to mouse sperm cells^56,57^, we could detect neither TADs (Fig. 8c) nor compartments (Fig. 8g) in *X. tropicalis* sperm cells. Together, these results show that the genome architecture is highly variable in different terminally differentiated tissues in *X. tropicalis*. How these different structures are established and whether they are essential for cell-type-specific gene expression is to be explored.

## Discussion

In this study, we showed that in *X. tropicalis*, TADs are established at ZGA. As the embryos develop, TADs continuously change their internal structure, with loops appearing at s11, which is followed by the emergence of stripes at later stages. Transcription inhibition by α-amanitin did not affect the formation of TAD structures at ZGA in either mouse or *Drosophila* embryos^18–20^. However, a more recent study showed that TAD establishment in human embryos requires transcription^32^. To determine whether this process is important in *Xenopus*, we took two approaches. First, we used morpholinos to deplete the expression of RPB1. Second, we inhibited transcription by α-amanitin. In either case, we found that TAD structures still formed in *X. tropicalis* embryos, suggesting that the requirement for transcription is more similar to fruit flies^19^ and mice^18,20^ but different from humans^32^. Recent high-resolution analysis using Micro-C also showed that acute inhibition of transcription had little effect on TAD structure in mouse stem cells^58^. However, the finding that RPB2 still binds to DNA after RPB1 knockdown suggests that the presence of the transcriptional machinery on chromatin might contribute to the formation of TADs, which highlights the importance of chromatin context in chromatin structure formation^59^.

Both CTCF and Rad21 are important for TAD establishment^24–32^. We showed that knockdown of CTCF and Rad21 disrupted TAD formation but not embryo development within the time frame we studied (no later than s23). Vietri and colleagues^60^ showed that chromosome domains are prominent in mammalian liver cells and evolutionarily conserved. We found that frog liver cells, which express low to barely detectable levels of CTCF, Rad21 and SNF2H, have very weak TAD structures. The observation of weak domain structures in frog liver cells suggests that chromatin organization might be associated with different metabolic states in amphibians and mammals. Whether transcription factor density affected higher-order chromatin structure formation^61,62^ or the low levels of examined proteins caused the lack of TAD structures in liver cells is to be investigated.

Cohesin-mediated extrusion occurs at loading sites before being stopped at a pair of convergent CTCF binding sites^24^. According to this model, CTCF and cohesin are not expected to be preferentially enriched at either side of TAD borders. However, our analysis unexpectedly revealed that for most TADs, CTCF and Rad21 are more enriched at one border than on the other. Accompanying this strikingly biased enrichment, the strength of the directionality of chromatin interaction at borders showed a similar pattern, which appears not to be caused by the simultaneous localization of TAD borders with compartment switching region or with hierarchical TAD borders (Supplementary Fig. 18).

Orientation-biased CTCF binding has been proposed to play a role in initiating cohesin-mediated extrusion, as inspired by the study of *Pcdh* loci^26,33^. Recent findings from the structural analysis of the cohesin–CTCF complex^63^ also explain orientation-biased CTCF and cohesin binding at TAD borders. Based on our findings, we speculate that the cohesin–CTCF complex, in some circumstances, may form a unique structure that allows extrusion to happen only in one direction until a barrier stops it. Our results also show that the chromatin remodeling factor ISWI is required for TAD formation, possibly through mediating CTCF binding^54^. Thus, a 3D chromosome conformation established from a structurally desolate genome may be initiated by pioneer factors binding and recruiting chromatin remodeling complexes, in this case ISWI, to DNA sequences remodeling chromatin into an accessible state for CTCF binding. Whether these events occur sequentially is to be explored.

The first version of the *X. tropicalis* reference genome was released ten years ago^64^ and has recently been updated to v.10.0. We fixed errors found in v.9.1 and generated a new high-quality reference genome that, together with v.10.0, now serves as a valuable resource for the wide research community using *X. tropicalis* to conduct genetic, genomic, molecular, developmental and evolutionary studies. Notably, both ours and the v.10.0 reference genome still contain errors that are visually identifiable and will require further improvement.

In summary, this work provides a systematic analysis of chromatin folding dynamics during embryogenesis through multiple distinct developmental phases and a high-quality reference genome for *X. tropicalis*. Together, these comprehensive datasets provide a rich resource for studying genome folding principles and the role of the 3D chromatin architecture in gene expression regulation, which governs cell differentiation and decides cell fate.

## Methods

### Contact for reagent and resource sharing

Further information and requests for reagents and resources should be directed to the Lead Contact, C. Hou (houch@sustech.edu.cn).

### Dataset description

We used PacBio (Pacific Biosciences of California) single-molecule sequencing (Supplementary Table 2) and Hi-C for de novo genome assembly. We generated 33 high-quality Hi-C datasets. At least two biological replicate libraries, unless otherwise stated, were generated and sequenced (Supplementary Table 3). We generated ChIP–seq datasets using CTCF, Rad21, RPB1 and RPB2 antibodies on WT embryos at s9, s11 and s13 and morpholino-injected embryos at s11 or s13.

### Frog strain

*X. tropicalis* frogs were purchased from Nasco and bred in an in-house facility. All experiments involving frogs were approved by the Institutional Animal Care and Use Committee at the Southern University of Science and Technology. All animal experiments were conducted in compliance with ethical guidelines. Ten pairs of one-year-old male and female adult frogs were used for in vitro fertilization; embryo developmental stages were determined according to Nieuwkoop and Faber^65^. Cerebral neurons and hepatocytes were isolated from two one-year-old male adult frogs and fixed for the Hi-C and ChIP experiments. Morpholinos were injected into embryos at the single-cell zygote stage.

#### Embryo collection for Hi-C

*X. tropicalis* embryos were obtained at different developmental stages by artificial fertilization. They were cultured in 0.1× MBS medium (1× MBS: 88 mM of NaCl, 2.4 mM of NaHCO_3_, 1 mM of KCl, 0.82 mM of MgSO_4_, 0.33 mM of Ca(NO_3_)_2_, 0.41 mM of CaCl_2_ and 10 mM of HEPES, pH 7.4) at 25 °C.

At the desired stages, embryos were fixed for 40 min in 1.5% formaldehyde. Fixation was stopped by a 10-min incubation in 0.125 M of glycine dissolved in 0.1× MBS, followed by three washes with 0.1× MBS. Fixed embryos were frozen at −80 °C in 1.5-ml microcentrifuge tubes (200 embryos per tube).

### Morpholino design and injection

The open reading frames of *X. tropicalis ctcf*, *rad21*, *rpb1* and *snf2h* were obtained by PCR with reverse transcription and cloned into the pCS2+ vector (Supplementary Table 4). Capped messenger RNA was generated with the mMESSAGE mMACHINE SP6 Transcription Kit (Thermo Fisher Scientific) and purified with the RNeasy Mini Kit (QIAGEN).

Morpholino antisense oligonucleotides (Gene Tools) to *ctcf*, *rad21*, *rpb1* and control morpholinos were injected separately into 1-cell stage embryos from the animal pole with a dose of 10–40 ng per embryo. The specificity of morpholino antisense oligonucleotide effects was confirmed by rescue experiments, where morpholino antisense oligonucleotides were coinjected with the corresponding mRNA. Embryo images were acquired with a microscope (Nikon). Morpholino antisense oligonucleotides for *ctcf*, *rad21*, *rpb1*, *snf2h* and Ctrl are listed in Supplementary Table 5.

### Hi-C library preparation

Generation of Hi-C libraries with low cell numbers was optimized according to a previous protocol^11^. Briefly, 100–600 embryos were cross-linked with 1% formaldehyde for 40 min using vacuum infiltration. Isolated embryo nuclei were digested with 80 U of DpnII (catalog no. R0543L; New England Biolabs) at 37 °C for 5 h. Restriction fragment overhangs were marked with biotin-labeled nucleotides. After labeling, chromatin fragments in proximity were ligated with 4,000 U of T4 DNA ligase for 6 h at 16 °C. Chromatin was reverse-cross-linked, purified and precipitated using ethanol. Biotinylated ligation DNA was sheared to 250–500-base pair (bp) fragments, followed by pull-down with MyOne Streptavidin T1 Dynabeads (catalog no. 65602; Thermo Fisher Scientific). Immobilized DNA fragments were end-repaired, A-tailed and ligated with adapters. Fragments were then amplified with the Q5 High-Fidelity 2X Master Mix (catalog no. M0492L; New England Biolabs). Hi-C libraries were sequenced on the Illumina HiSeq X10 platform (paired-end 2 × 150-bp reads).

### Western blot analysis

*X. tropicalis* embryos and tissues at the indicated stages/ages were collected and homogenized in radioimmunoprecipitation assay buffer (Thermo Fisher Scientific) with a proteinase inhibitor cocktail (Merck). Lysates were mixed with 2 volumes of 1,1,2-Trichlorotrifluoroethane (Macklin) and centrifuged at 4 °C, 13,000*g* for 15 min. Supernatants were mixed with an equal volume of 2× loading buffer and boiled for 5 min. A total of 10 μg of protein was loaded onto a 10% SDS–polyacrylamide gel electrophoresis gel, electrophoresed and transferred to a polyvinylidene fluoride membrane (Bio-Rad Laboratories). The membrane was blocked with 5% nonfat milk in 1× TBST (a mixture of Tris-buffered saline and 0.1% Tween 20) buffer for 1 h at room temperature and incubated overnight with primary antibody at 4 °C. Anti-RPB1 (catalog no. 664906; BioLegend), anti-CTCF (catalog no. 61311; Active Motif), anti-Rad21 (catalog no. ab992; Abcam), anti-SNF2H (catalog no. orb154213; Biorbyt), anti-β-tubulin (catalog no. ab6046; Abcam) and anti-histone H3 (catalog no. B1005; Biodragon) were all used at concentrations of 1/3,000 in 10 ml of 1× TBST/100 mg BSA. β-Tubulin and histone H3 were used as loading controls. After five times of washing with 1× TBST buffer for 10 min, the membrane was incubated with either goat anti-rabbit or goat anti-mouse horseradish peroxidase-conjugated secondary antibody diluted 10,000 times (catalog nos. HS101-01 and HS201-01; Transgen Biotech) for 2 h at room temperature. The signal was detected using a chemiluminescence western blot detection kit (Millipore).

### ChIP library preparation

The ChIP assay was performed as described by Akkers et al.^66^. Briefly, 200–600 embryos were cross-linked with 1% formaldehyde for 40 min using vacuum infiltration. Human K562 cells were added as the spike-in control. Chromatin was sheared to an average size of 150 bp using a sonicator (Bioruptor Pico; Diagenode). Sonicated chromatin fragments were immunoprecipitated with 3 μg of anti-Rad21, anti-RPB1, anti-CTCF and anti-RPB2 (catalog no. A5928; ABclonal). Chromatin-bound antibodies were recovered with 30 μl of Protein A/G Magnetic Beads (catalog no. 16-663; Millipore). After reverse cross-linking, ChIP-ed DNA was recovered using the MinElute Reaction Cleanup Kit (catalog no. 28206; QIAGEN) and amplified with the VAHTS Universal DNA Library Prep Kit for Illumina V3 (catalog no. ND607-01; Vazyme). Amplified ChIP libraries were sequenced on the Illumina HiSeq X10 platform.

### Quantification and analyses

#### Hi-C sequence alignment and quality control

All Hi-C datasets were processed using the Juicer pipeline^67^ or distiller v.0.3.3 (https://github.com/open2c/distiller-nf); reads with a mapping quality score <1 were filtered out and discarded. Reads were aligned against the *X. tropicalis* v.9.1, our assembled and v.10.0 reference genomes and the PacBio contigs, respectively. Replicates were merged by Juicer’s mega.sh script. All contact matrices used for further analysis were KR-normalized with Juicer v.1.5. VC_SQRT-normalized matrices were used when the KR-normalized matrix was not available.

#### Genome assembly

We first assembled PacBio reads into raw contigs. De novo assembly of the long reads from single-molecule, real-time (SMRT) sequencing was performed using the SMRT Link HGAP4 application with default parameters. We then scaffolded these raw contigs into chromosome-scale scaffolds. Hi-C data derived from s9 cells were selected as assembly evidence considering their low rate of long-range contact and adequate valid interactions. Mapping, filtering, deduplication, merging replicates and scaffolding of contigs based on Hi-C contact were processed by Juicer^67^ and 3D-DNA^43^. We skipped the misjoin detection step because of its high false positive rate in the contigs derived from the PacBio long reads. Instead, we manually refined the genome assembly after scaffolding using the Juicebox Assembly Tools v.1.11.08 (ref. ^42^) to correct several obvious errors. Note that our new assembly still has some small-scale errors to be corrected.

We then assigned the chromosome number to each chromosome-scale scaffold after the assembly of raw contigs. Genetic markers^68^ were mapped to chromosome-scale scaffolds to determine their chromosome ID and reorient their directions. MAKER v.2.31.10 (ref. ^44^) was used to map the previous annotation of *X. tropicalis* to our new genome assembly.

#### Genome assembly statistical analysis

To make a comparison between the previous assembly and our new assembly, we used MUMmer4 v.4.0 (ref. ^69^) (command: nucmer -t 20 -g 50000 -c 1000 -l 1000 --mum) to align them. Alignments between the two assemblies were then visualized using the R basic graphic package v.3.5. The locations of chromosome centromeres were determined visually based on the Hi-C heatmap. For the profile plot of genome assembly, we generated an AGP file based on the 3D-DNA output after completing the genome assembly. The profile plot of the genome assembly is based on the AGP file.

#### Insulation, TAD and TAD border calling

To check the contact domain properties of each sample, we also calculated the DI and insulation score as defined previously^4,45^ using a parallel script based on a 10-kb resolution (using a triplet format matrix). DI and insulation scores were both calculated with a block size of 400 kb (40 bins).

For the domain analysis, we first annotated the contact domain by using three methods (arrowhead^11,67^, rGMAP^70^ and TopDom^71^) at 10-kb resolutions using default parameters and merged the results. For the arrowhead method, we used 0.5 as a variance threshold instead of the default value 0.2. For rGMAP, nested domains were detected with the parameter dom_order = 2. For TopDom, the window size was set to 200 kb.

The domains called by arrowhead, rGMAP and TopDom were then filtered and merged. We defined a metric ‘diamond score’ to measure the strength of the domain and used it to filter out domains with a low diamond score. The diamond score was calculated as a blow for domain *D* in a Hi-C matrix *M*.

$${\mathrm{DS}}_D = \frac{{\mathop {\sum}\nolimits_{i,jD_{\mathrm{M}}} {M_{i,j}} }}{{\mathop {\sum}\nolimits_{i,jD_{\mathrm{U}}\cup D_{\mathrm{M}}} {M_{i,j}} }}$$

where *D*_M_, *D*_U_ and *D*_D_ denote the middle, upstream and downstream diamond areas of the domain (black triangle) (Supplementary Fig. 19).

Domains with a diamond score lower than 0.6 and domain size lower than 100 kb were filtered. Then, the domains detected by the three methods were merged and the boundary was aligned. Domain boundaries within a 4-bin window were merged and set to the bin with the lowest insulation score. Finally, we excluded domains located in an area with low contact density as in-loop filtering.

The same domain between two domain sets was judged using the BEDTools v.2.29.0 (ref. ^72^) intersect command with -f 0.9 --r. To compare domain sets from different experiments, we counted overlapped domains using the BEDTools intersect command with -f 0.7 --r.

#### TAD clustering

We used the *k*-means clustering method to classify domains from each sample by using deepTools v.3.1.3 (ref. ^73^). Domains were clustered based on the DI values within 10 bins around the 5′ and 3′ TAD borders (±5 bins, 5 kb per bin), respectively. We also calculated the adjusted DI for each domain and did *k*-means clustering to classify domains based on the adjusted DI vectors.

#### Loop domain identification, TAD aggregation and comparison

The HiCCUPS algorithm^67^ was used to call chromatin loops for each matrix at 5- and 10-kb resolutions. To avoid the false positives of the HiCCUPS algorithm, we filtered loops in two steps. We first filtered loops whose contact distance was larger than 2 megabases (Mb). Then, we filtered loops whose surrounding (±5 kb) Vanilla-Coverage normalization values were <1. Loop domains were annotated as described previously^11^ by searching for loop–domain pairs where the peak pixel was within the smaller of 50 kb or 0.2 of the domain’s length at the corner of the domain.

To further check the domain’s validation, we aggregated each domain or loop domain set as described previously^29^. After aggregation, we divided each aggregated matrix by its mean value for normalization. Note that for the analysis of the knockdown effect, the aggregated matrices were calculated based on the control domain set.

#### DI bias

The DI bias at the 5′ and 3′ borders for each TAD was calculated using the formula shown below:

$${\rm{DI}}\ {\rm{bias}}= {\rm{log}}_2\left( {\mathrm{abs}\left( {\frac{{\mathrm{DI}_{\rm{mean}}^{5\prime }}}{{\mathrm{DI}_{\rm{mean}}^{3\prime }}}} \right)} \right)$$

where $\mathrm{DI}_{\rm{mean}}^{5\prime }$is the mean DI value of the 3 bins (15 kb) on the right-hand side of the 5′ TAD border and $\mathrm{DI}_{\rm{mean}}^{3\prime }$ is the mean DI value of the 3 bins (15 kb) on the left-hand side of the 3′ TAD border.

#### CTCF motif enrichment around TAD borders

To profile the CTCF motif enrichment around domain borders, we first obtained the CTCF motif location and direction in our new genome assembly using the HOMER v.4.1.0 scanMotifGenomeWide.pl script^74^ The enrichment of CTCF in the sense and antisense strands was computed and plotted using deepTools.

#### Compartment analysis

For the compartment analysis, CscoreTool^55^ was used to call the compartment at a 25-kb resolution. Original Cscore directions were adjusted for s23 using the ChIP–seq signals of active histone modifications^75^. We used the adjusted Cscores as matrix projection vectors and multiplied them by the observed/expected Hi-C matrices of other developmental stages to calculate the compartment scores. Compartment scores were further normalized by subtracting their means and then scaled between −1 and 1. We used the newly acquired compartment scores to assign compartments A and B.

To track the dynamics of the domain and compartment structure during embryo development, we performed a PCA analysis of both DI- and Cscore-derived compartment scores. The R package ggbiplot v.0.55 was used to plot the PCA analysis results of the DI- and Cscore-derived compartment scores.

#### ChIP–seq analysis

ChIP–seq reads were mapped to the *X. tropicalis* v.10.0 reference genome with the Burrows–Wheeler Aligner^76^ and analyzed with MACS v.2.0 (ref. ^77^). All data were normalized against the corresponding input control using the ‘-c’ option of MACS v.2.0. Alignments of replicates were merged for downstream analysis.

Signal tracks were calculated using the bdgcmp option of MACS v.2.0 with the fold enrichment method. All data for downstream analyses were averaged and extracted from these tracks.

#### Spike-in ChIP–seq analysis

Paired-end reads were mapped to the genome index generated by both the hg19 and Xenbase v.10 (X.tr_v10) genomes. Reads with mapQ values <1 or without mate correctly mapped were filtered. We estimated the library size of each sample based on the ratio between the number of reads mapped to X.tr_v10 and hg19:

$$S_i \approx R_i = \frac{{N_i^X}}{{N_i^H}}$$

*S*_*i*_ and *R*_*i*_ represent the library scale factor and ratio between the number of reads mapped to X.tr_v10 and hg19. Signal tracks were calculated using the deepTools bamCoverage command and normalized by library size.

#### Spike-in RNA sequencing analysis

We first mapped the spike-in RNA sequencing reads to the hg19 and X.tr_v10 genomes separately using STAR v.2.7.1a^78^. Then, the library size of each experiment was estimated as with the spike-in ChIP–seq. The read count mapped for each gene was calculated by HTSeq-count v.0.11.1 (ref. ^79^) and then normalized by library size.

### Reporting Summary

Further information on research design is available in the Nature Research Reporting Summary linked to this article.

## Online content

Any methods, additional references, Nature Research reporting summaries, source data, extended data, supplementary information, acknowledgements, peer review information; details of author contributions and competing interests; and statements of data and code availability are available at 10.1038/s41588-021-00878-z.

## Supplementary information

Supplementary Information Supplementary Figs. 1–19 and Source Data of western blots for Supplementary Figs. 3 and 10

Reporting Summary

Supplementary Tables 1–5 Supplementary Table 1: Comparison of the three versions of the *Xenopus tropicalis* reference genome. Supplementary Table 2: PacBio sequencing statistics. Supplementary Table 3: Hi-C sequencing statistics. Supplementary Table 4: Gene sequences for rescue experiments. Supplementary Table 5: Morpholino antisense oligonucleotides for knockdown experiments.

## Data Availability

All raw sequencing data generated in this study have been deposited with the BioProject database (http://www.ncbi.nlm.nih.gov/bioproject) under accession no. PRJNA606649. Processed ChIP–seq data and identified domains are available at 10.6084/m9.figshare.14377283.v2. H3K4me1, H3K4me3, H3K9me2, H3K27me3, H3K36me3 and p300 ChIP–seq data in *X. tropicalis* embryos were obtained from the Gene Expression Omnibus (GEO) (accession no. GSE67974). The RNA-seq analysis data during *X. tropicalis* embryonic development were obtained from the GEO (accession no. GSE65785). CTCF ChIP–seq data in human K562 were obtained from the Encyclopedia of DNA Elements (ENCODE) (accession no. ENCFF675GVW). Cohesin Rad21 ChIP–seq data in human K562 were obtained from ENCODE (accession no. ENCFF000YXZ). CTCF ChIP–seq data in *Drosophila* S2 were obtained from ENCODE (accession no. ENCFF512CQC). Hi-C data in human K562 were obtained from the GEO (accession no. GSE63525). SAFE Hi-C data in *Drosophila* S2 were obtained from BioProject (accession no. PRJNA470784). Source data are provided with this paper.

## Source data

Source Data Fig. 4 Unprocessed western blots for Fig. 4. Source Data Fig. 5 Unprocessed western blots for Fig. 5. Source Data Fig. 6 Unprocessed western blots for Fig. 6. Source Data Fig. 8 Unprocessed western blots for Fig. 8.

## Code availability

The custom codes used in this study are available at https://github.com/shenscore/Xenopus_Hi-C.
