# Supplementary material for: Three-dimensional folding dynamics of the Xenopus tropicalis genome
Source: Nat Genet. 2021 Jun 7;53(7):1075–87. doi: 10.1038/s41588-021-00878-z (PMC8270788; doi:10.1038/s41588-021-00878-z)

nature portfolio

https://doi.org/10.1038/s41588-021-00878-z

Supplementary information

Three-dimensional folding dynamics of the *Xenopus tropicalis* genome

In the format provided by the
authors and unedited

(Supplementary information contains 19 figures, 5 tables, and 3 sourcedata files.)

Supplementary Fig. 1│Heatmap of each chromosome. Assembly errors are mostly corrected in both Niu et al. and v10.0 versions of the reference genome. Arrows point to obvious errors in both Niu et al. and v10.0 versions.

Supplementary Fig. 2│Genome-wide contact heatmap. Centromere interactions are weak using the v9.1 *X. tropicalis* reference genome. Genome-wide contact heatmaps show intra-chromosome arm interactions and inter-chromosome interactions between centromeres using Niu et al. and v10.0 versions of the reference genome.

Supplementary Fig. 3│ Convergent CTCF site distribution and western blot.

a, Accumulated convergent CTCF sites observed at all borders of TADs (in ten thousands) identified at all developmental stages.

b, Western blot of CTCF and Rad21 at different developmental stages. Western blot experiments in this figure were repeated for at least two times with similar results.

Supplementary Fig. 4|CTCF ChIP-seq on wild type embryos.

a, CTCF ChIP-seq on embryos from stages 8, 9, 11, and 13 normalized to

CTCF ChIP-seq signal from spike-in K562 cells.

b, CTCF ChIP-seq on spike-in K562 cells.

Supplementary Fig. 5│Rad21 ChIP-seq on wild type embryos

a, Rad21 ChIP-seq on embryos from stages 9, 11 and 13 normalized to Rad21 ChIP-seq signal from spike-in K562 cells.

b, Rad21 ChIP-seq on spike-in K562 cells.

Supplementary Fig. 6│ Examples of TADs for the three different clusters**.**

a, b, and **c,** Example TADs in clusters 1, 2, and 3. Paired TADs in hierarchical TAD can also be seen in **c**. Red, black, and blue triangles indicate TADs in clusters 1, 2, and 3, respectively.

Supplementary Fig. 7│**Directionality index analysis in human K562 cells.**

a, Directionality index across borders of the three clusters of TADs in K562 cells. Cluster 2 shown in the DI heatmap is further divided into five sub-clusters with the same number of TADs.

b, Enrichment of CTCF is biased to borders with higher directionality index values for the three clusters of TADs. CTCF enrichment is shown in the heatmap.

c, Enrichment of Rad21 is biased to borders with higher directionality index values for the three clusters of TADs. Rad21 enrichment is shown in the

heatmap. Data in upper panels of **a**, **b,** and **c** are represented as mean±SEM.

d, Directionality bias index for the five sub-clusters of cluster 2. According to the rank of directionality index strength, TADs in cluster 2 are divided into five sub-clusters. Data are presented as boxplots with violinplots. The minima, maxima, center, and bounds of each boxplot refers to Q1-1.5IQR, Q3+1.5IGR, median, first quartile (Q1) and third quartile (Q3) of data. IQR, interquartile range.

e, Enrichment of CTCF is slightly biased to borders with a higher directionality index for the five sub-clusters of cluster 2. According to the rank of directionality index strength, TADs in cluster 2 are divided into five sub-clusters.

f, Enrichment of Rad21 is slightly biased at borders with a higher directionality index for the five clusters 2 sub-clusters. According to the rank of directionality index strength, TADs in cluster 2 are divided into five sub-clusters. Data in **e** and **f** are represented as mean±SEM.

Supplementary Fig. 8|Directionality index analysis in *Drosophila* S2 cells.

a, Directionality index across borders of clustered TADs of *Drosophila* S2 cells. 150, 466, and 182 TADs in Clusters 1, 2, and 3, respectively. C, cluster.

b, Violin plot showing the distribution of directionality bias index of clusters 1, 3, and five sub-clusters of cluster 2 in *Drosophila* S2 cells. Data are presented as boxplots with violinplots. The minima, maxima, center, and bounds of each

boxplot refers to Q1-1.5IQR, Q3+1.5IGR, median, first quartile (Q1) and third quartile (Q3) of data. IQR, interquartile range.

c, Relative enrichment of CTCF at the borders of three clusters of TADs showing the lack of correlation with the inequality in directionality for three clusters of TADs in *Drosophila* S2 cells. Data in **a** and **c** are represented as mean±SEM.

Supplementary Fig. 9|RNA expression was reduced after *rpb1* knock-down and transcription inhibition.

a, RNA expression compared to s9 and s13 wild-type embryos after *rpb1* was knocked-down.

b, RNA expression compared to s9 and s11 wild type embryos after transcription inhibition.

RPKM, reads per kilobase of transcript, per million mapped reads.

Supplementary Fig. 10│Effects of *rpb1* knock-down on RPB2 binding.

a, RPB2 expression in wild-type embryos at stages 8, 9, 11 and 13.

b, Western blot of proteins in embryos with RPB1 knocked-down by morpholinos and in embryos that were rescued. Morpholino control (ctrl), no morpholino (-), rpb1 morpholino (+), rpb1 rescue (rsc). Western blot experiments in this figure were repeated for at least two times with similar results.

c, RPB2 ChIP-seq signal across genes normalized to spike-in K562.

d, RPB2 ChIP-seq signal across human genes in spike-in K562 cells. For **c** & **d**, Wild type (wt), morpholino control (ctrl), *rpb1* morpholino knock-down (rpb1 kd), *rpb1* rescue (rpb1 rsc), all experiments were conducted in two biological replicates.

Supplementary Fig. 11|CTCF ChIP-seq for knock-down analysis.

a, CTCF ChIP-seq for CTCF and Rad21 knock-down experiments normalized to spike-in K562.

b, CTCF ChIP-seq for K562 cells spiked-in CTCF and Rad21 knock-down experiments. Wild type (wt), morpholino control (ctrl), *ctcf* morpholino (ctct kd), *ctcf* rescue (ctcf rsc), *rad21* morpholino (rad kd), rad21 rescue (rad rsc), *ctcf* and *rad21* morpholinos (dbl kd), *ctcf* and *rad21* rescue (dbl rsc), all experiments were carried out in two biological replicates.

Supplementary Fig. 12|Rad21 ChIP-seq for knock-down analysis.

a, Rad21-ChIP seq for CTCF and Rad21 knock-down experiments normalized to spike-in K562.

b, Rad21-ChIP seq for K562 cells spiked-in CTCF and Rad21 knock-down experiments. Wild type (wt), morpholino control (ctrl), *ctcf* morpholino (ctct kd), *ctcf* rescue (ctcf rsc), *rad21* morpholino (rad kd), rad21 rescue (rad rsc), *ctcf* and *rad21* morpholinos (dbl kd), *ctcf* and *rad21* rescue (dbl rsc), all

experiments were carried out in two biological replicates.

Supplementary Fig. 13|Effects of *ctcf* and *rad21* knock-down on chromatin structure.

a, Example region to show the knock-down effect on TAD structures.

b, Correlation of insulation scores for the borders of identified TADs in wild type embryos at s13 vs control, knock-down, and rescued embryos at s13.

c, Number of TADs and size distribution.

d, Percentage of genome folds into TADs.

Supplementary Fig. 14│ Effects of *ctcf* and *rad21* knock-down on embryo development. Knock-down of *ctcf* and *rad21* were repeated for at least two times with similar results.

Supplementary Fig. 15|CTCF ChIP-seq on *snf2h* knock-down cells.

a, CTCF ChIP-seq on *snf2h* knock-down embryos normalized to spike-in K562 signal.

b, CTCF ChIP-seq on spike-in K562 cells. Wild type (wt), morpholino control (ctrl), *snf2h* morpholino (*snf2h* kd), *snf2h* rescue (*snf2h* rsc), all experiments were conducted in two biological replicates.

Supplementary Fig. 16│ Effects of *snf2h* knock-down.

a, Number of TADs and TAD size distribution.

b, Percentage of genome folds into TADs.

c, Effect of *snf2h* knock-down on embryo development. Knock-down of *snf2h* were repeated for at least two times with similar results.

Supplementary Fig. 17|Chromatin contact heatmaps.

Heatmaps of 50kb resolution for a 30Mb region in chromosome 2 from multiple developmental stages show continuous compartmentalization.

Supplementary Fig. 18|Coincident localization of TAD border with hierarchical TAD border and compartment switch regions in stage 13 embryos of *X. tropicalis*.

a, Percentage of the three clusters TAD that overlap with hierarchical TAD or being singleton TAD.

b, Number of TADs from cluster 1 and cluster 3 that are paired in hierarchical TAD.

c, Percentage of the three clusters TAD that overlap with compartment switch region with random genomic regions as background control.

Supplementary Fig. 19|. Diamond areas for domain "diamond score" calculation. M, U, and D denote the middle, upstream, and downstream diamond areas of the domain (black triangle).

Supplementary Table 1 | Comparison of the three versions of the *Xenopus tropicalis* reference genome.

Supplementary Table 2 | PacBio sequencing statistics.

Supplementary Table 3 | Hi-C sequencing statistics.

Supplementary Table 4 | Gene sequences for rescue experiments.

Supplementary Table 5 | Morpholino antisense oligonucleotides for knock-down experiments.

Niu_SourceData_SupplementaryFig3b

Niu_SourceData_SupplementaryFig10a

Niu_SourceData_SupplementaryFig10b

Supplementary Fig. 1

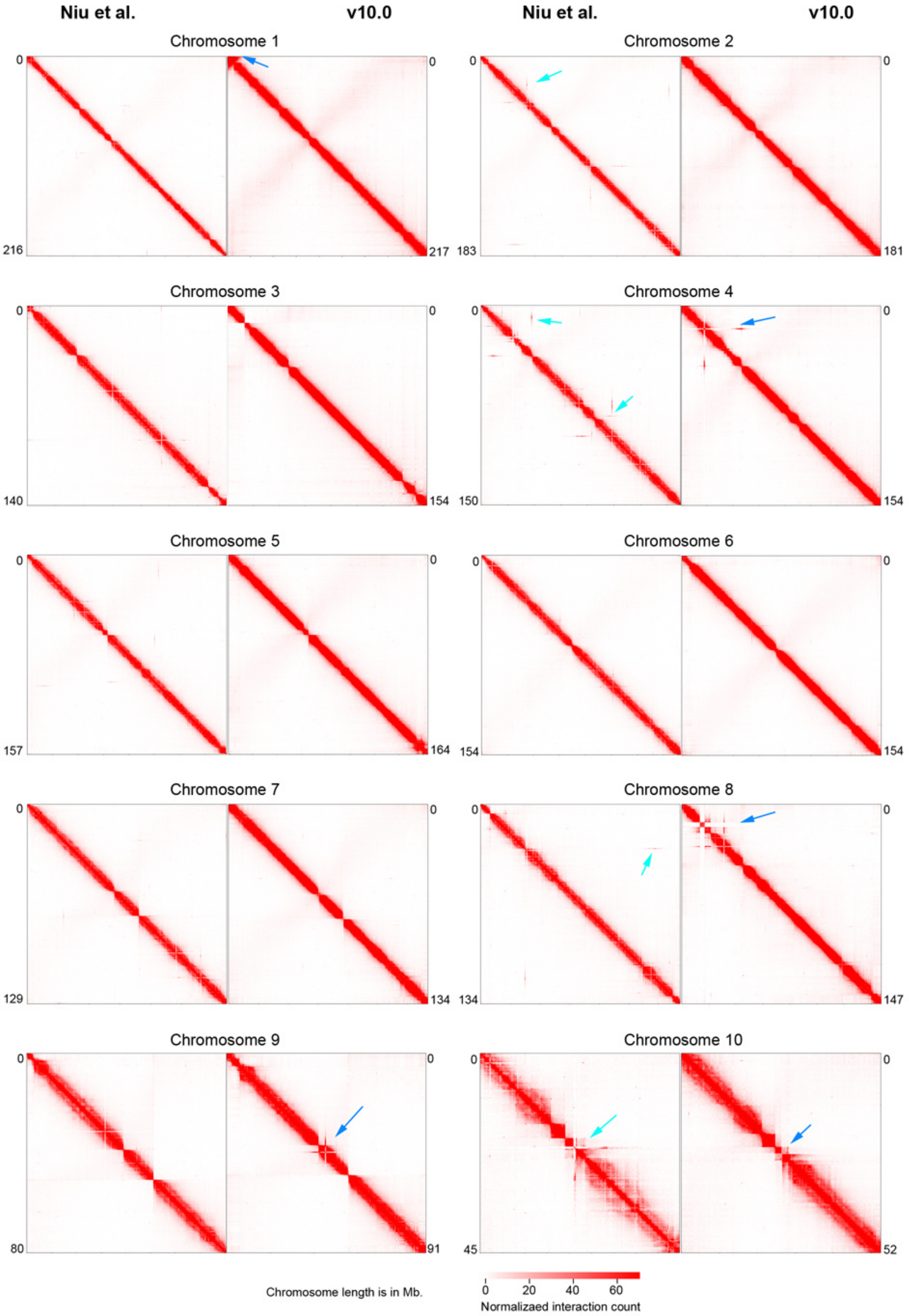

Supplementary Fig. 2

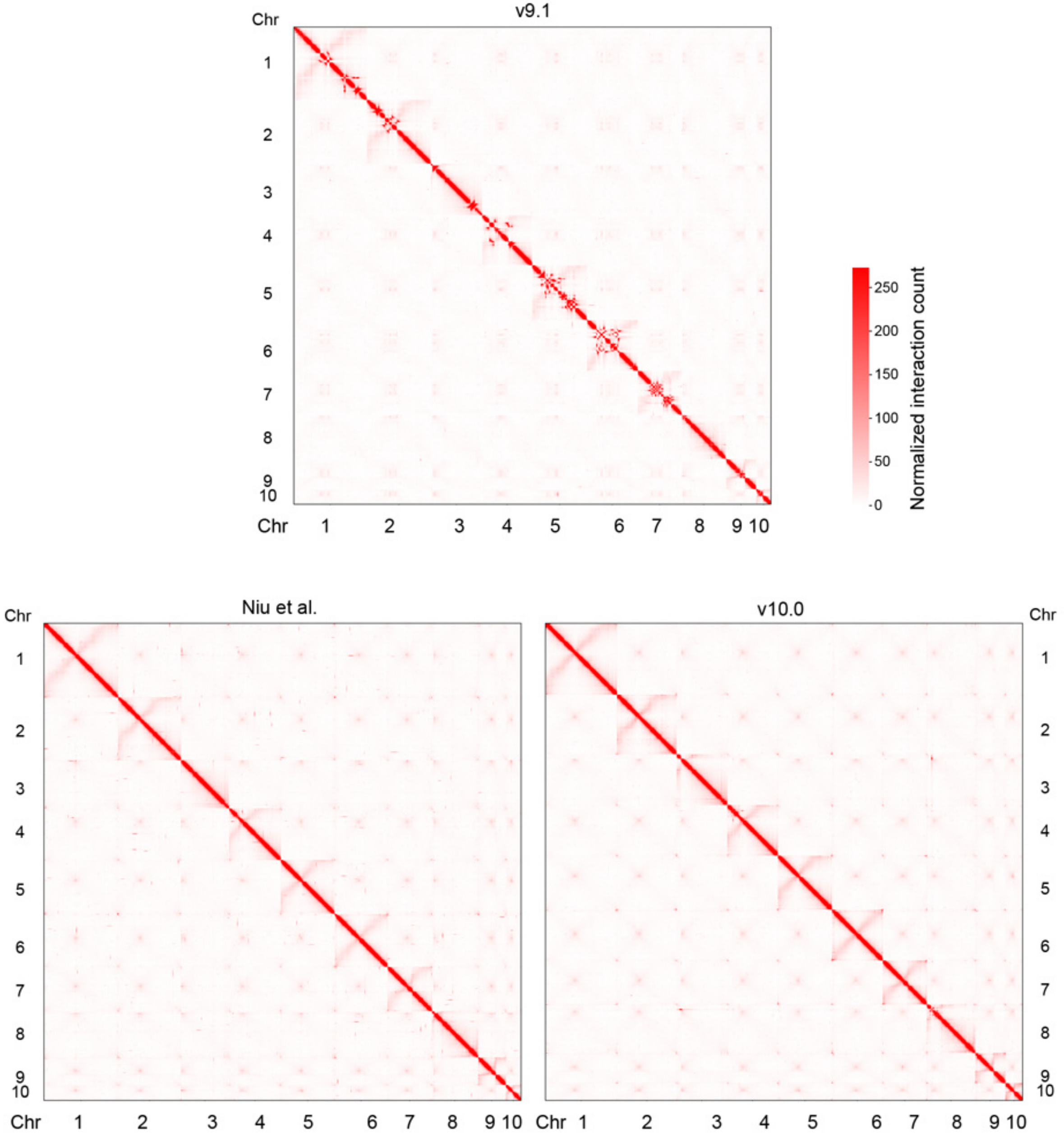

Supplementary Fig. 3

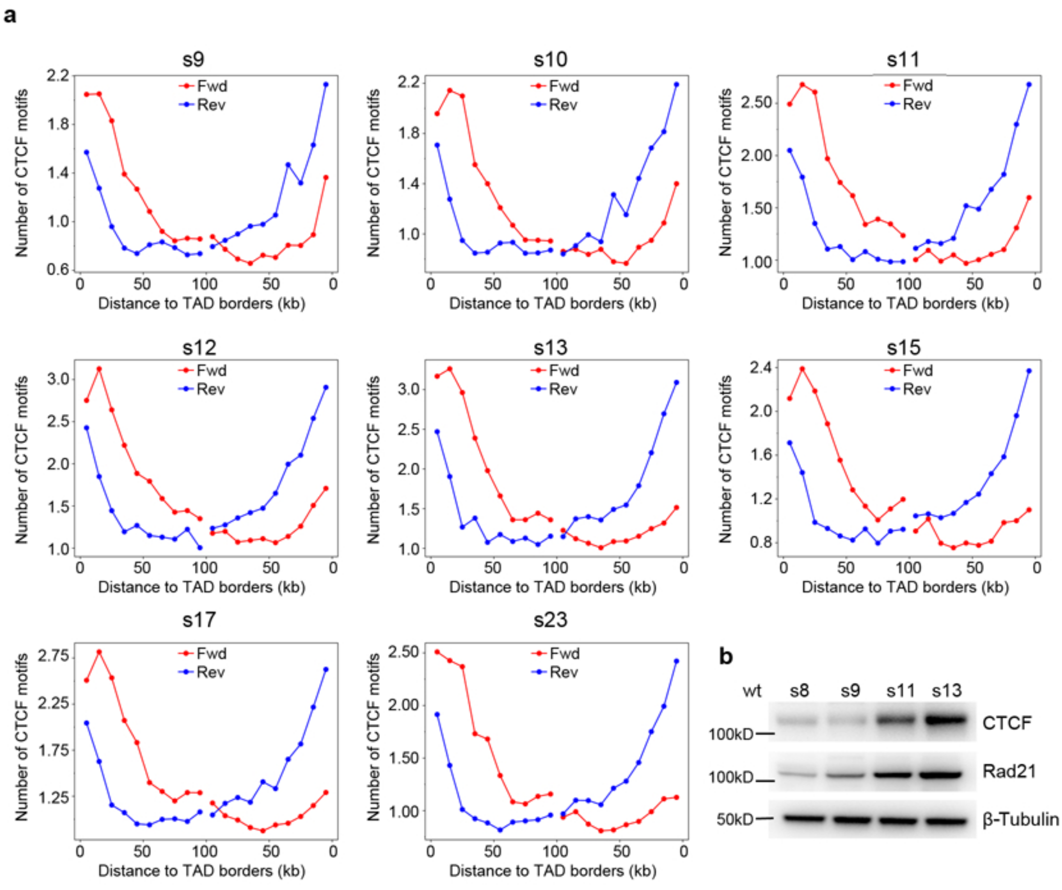

Supplementary Fig. 4

a

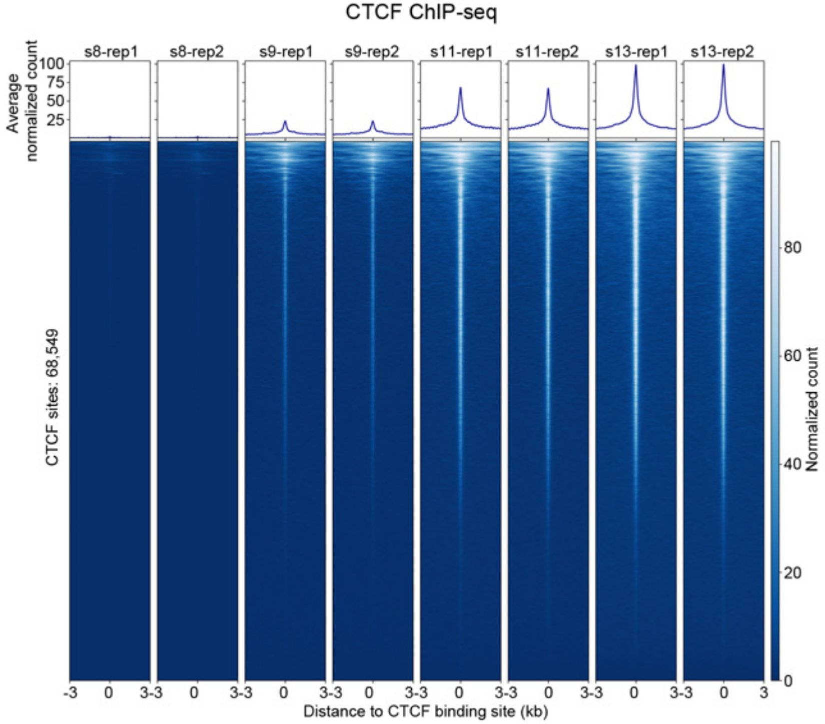

b

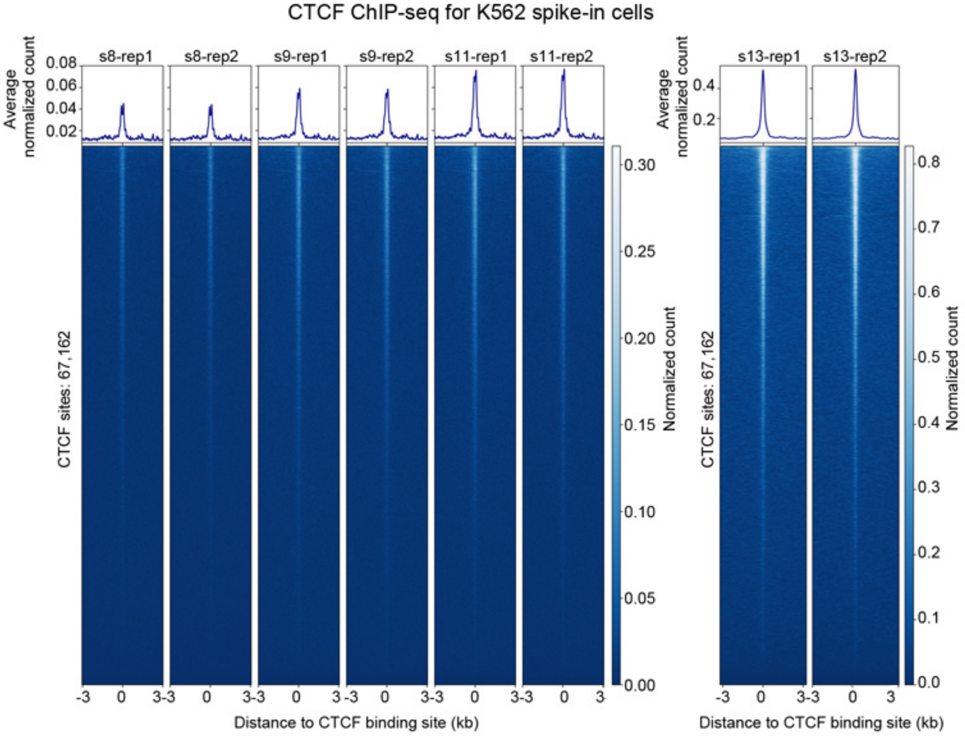

Supplementary Fig. 5

a

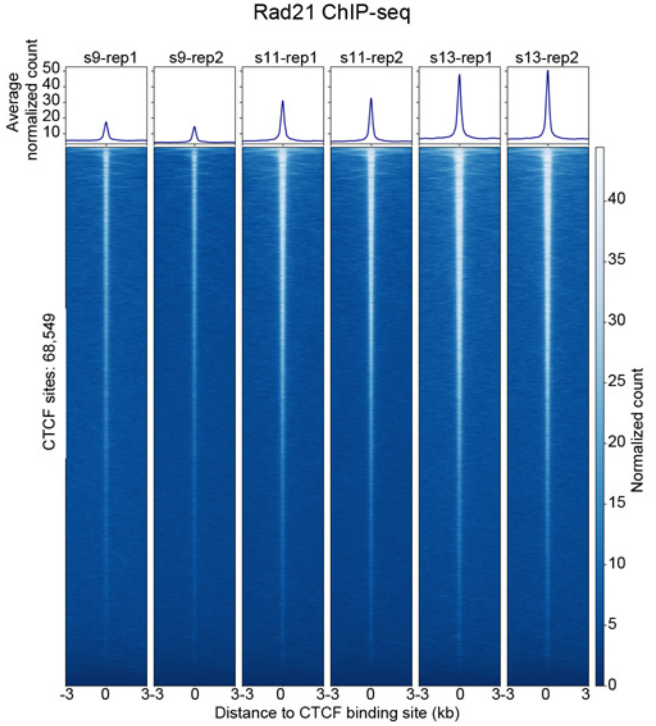

b

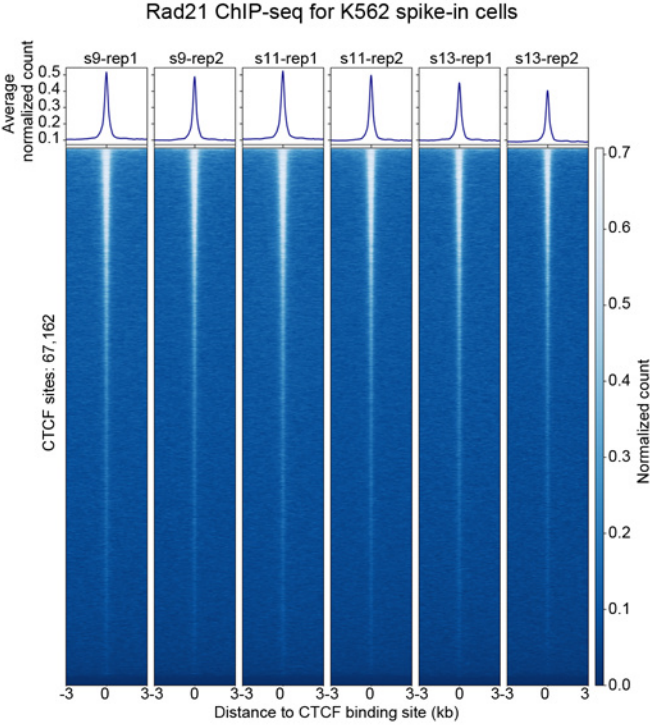

Supplementary Fig. 6

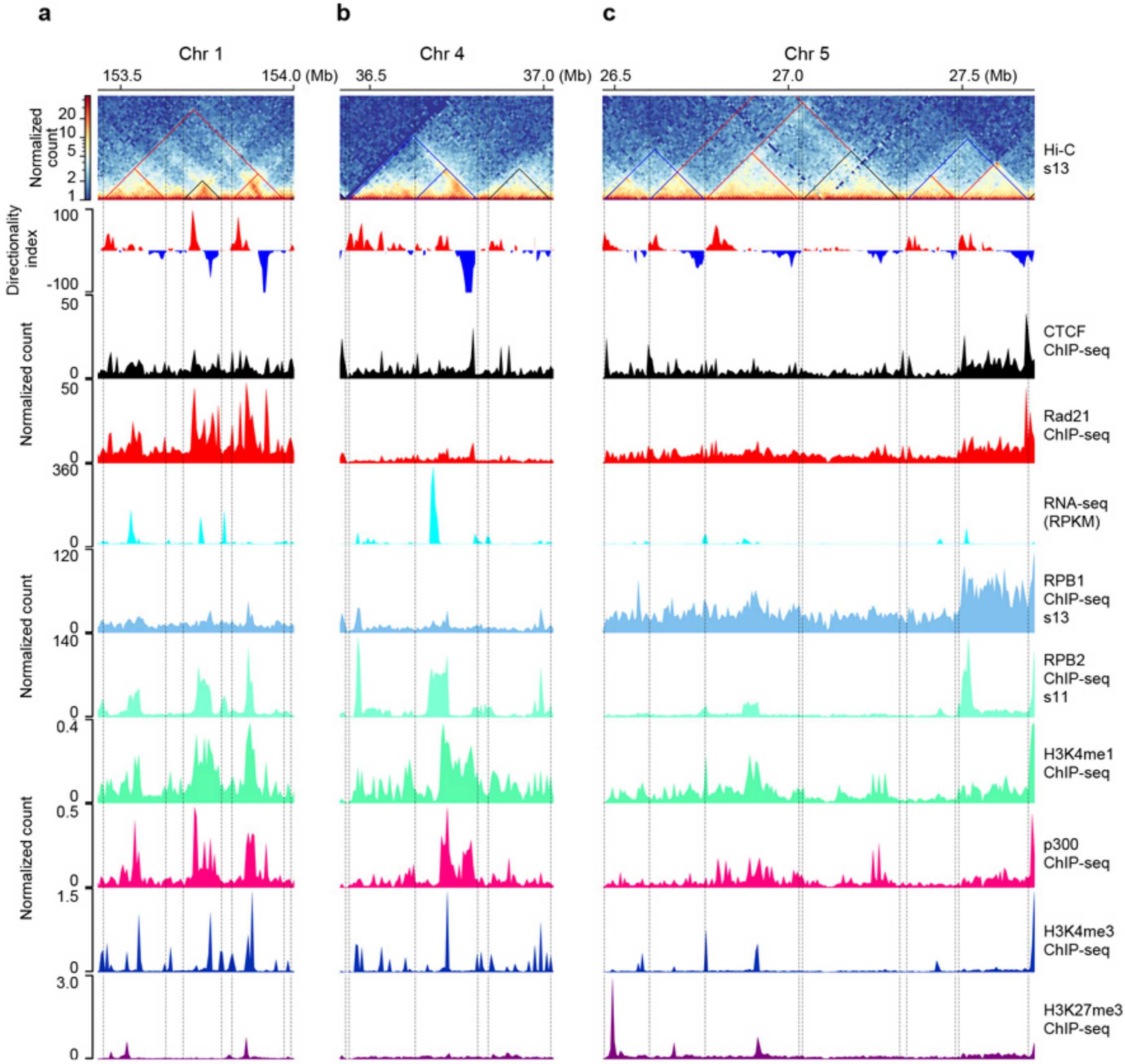

Supplementary Fig. 7

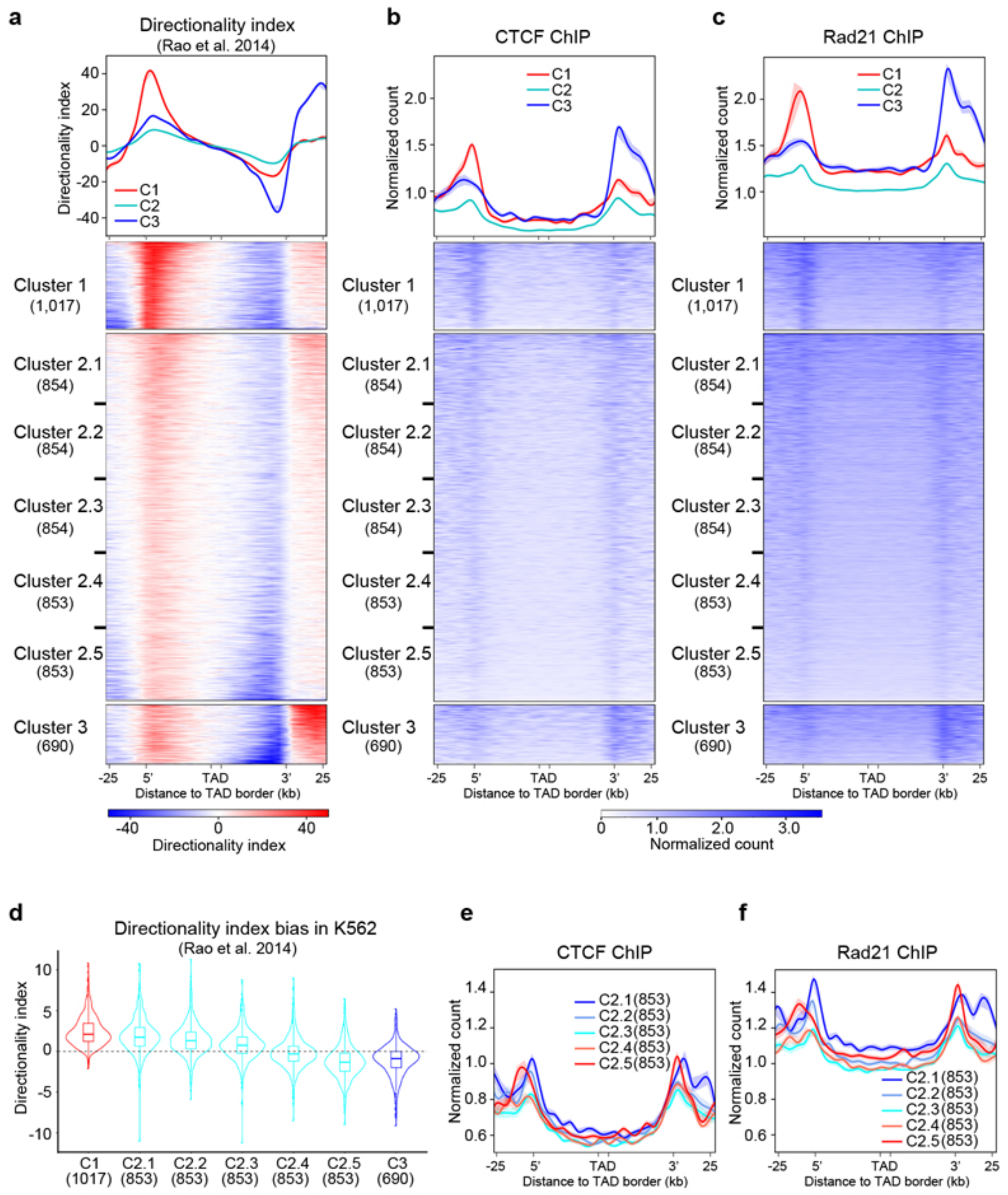

Supplementary Fig. 8

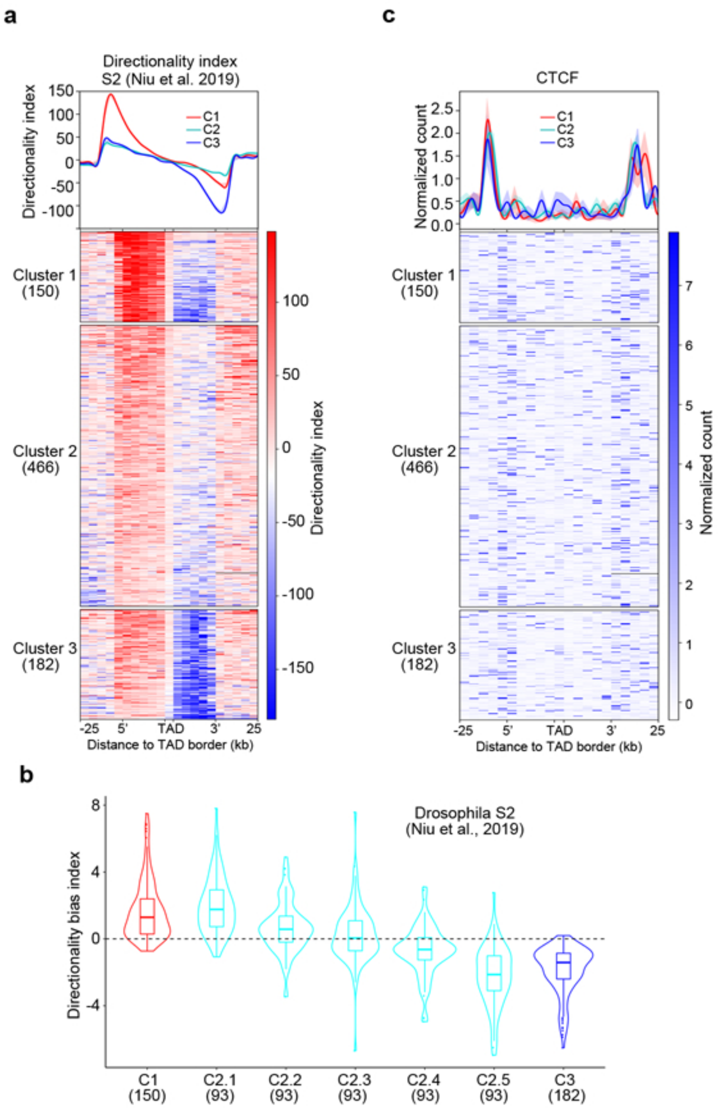

Supplementary Fig. 9

a

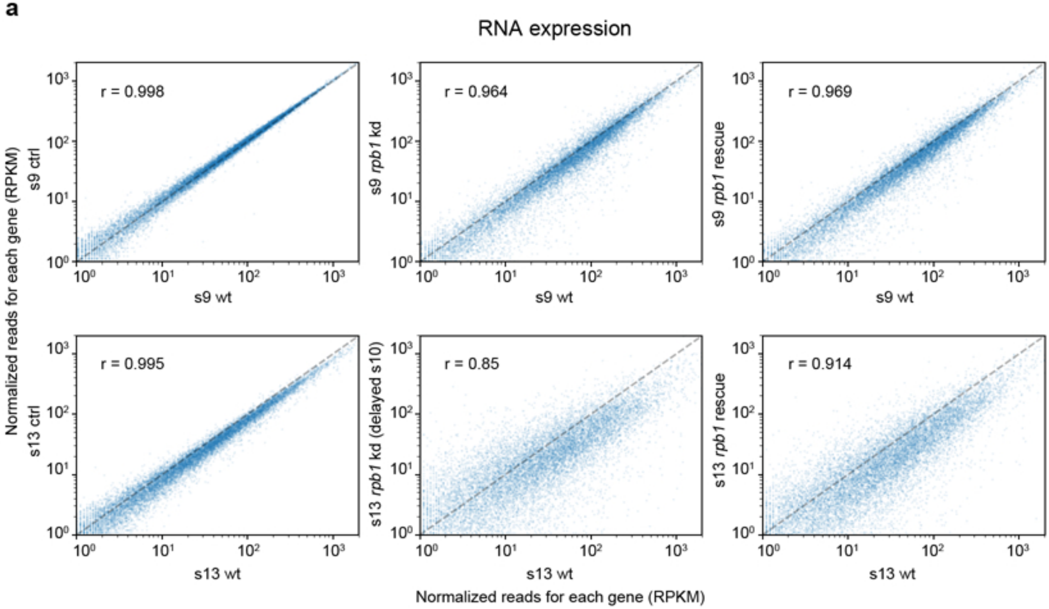

b

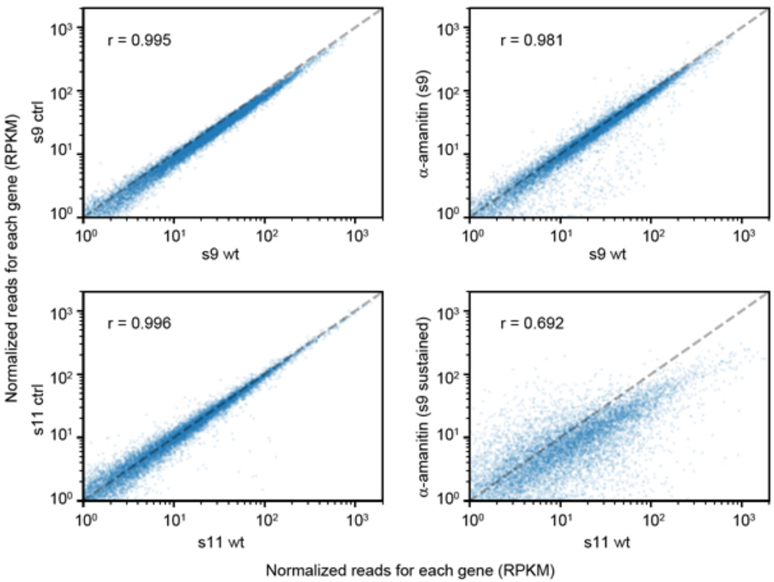

Supplementary Fig. 10

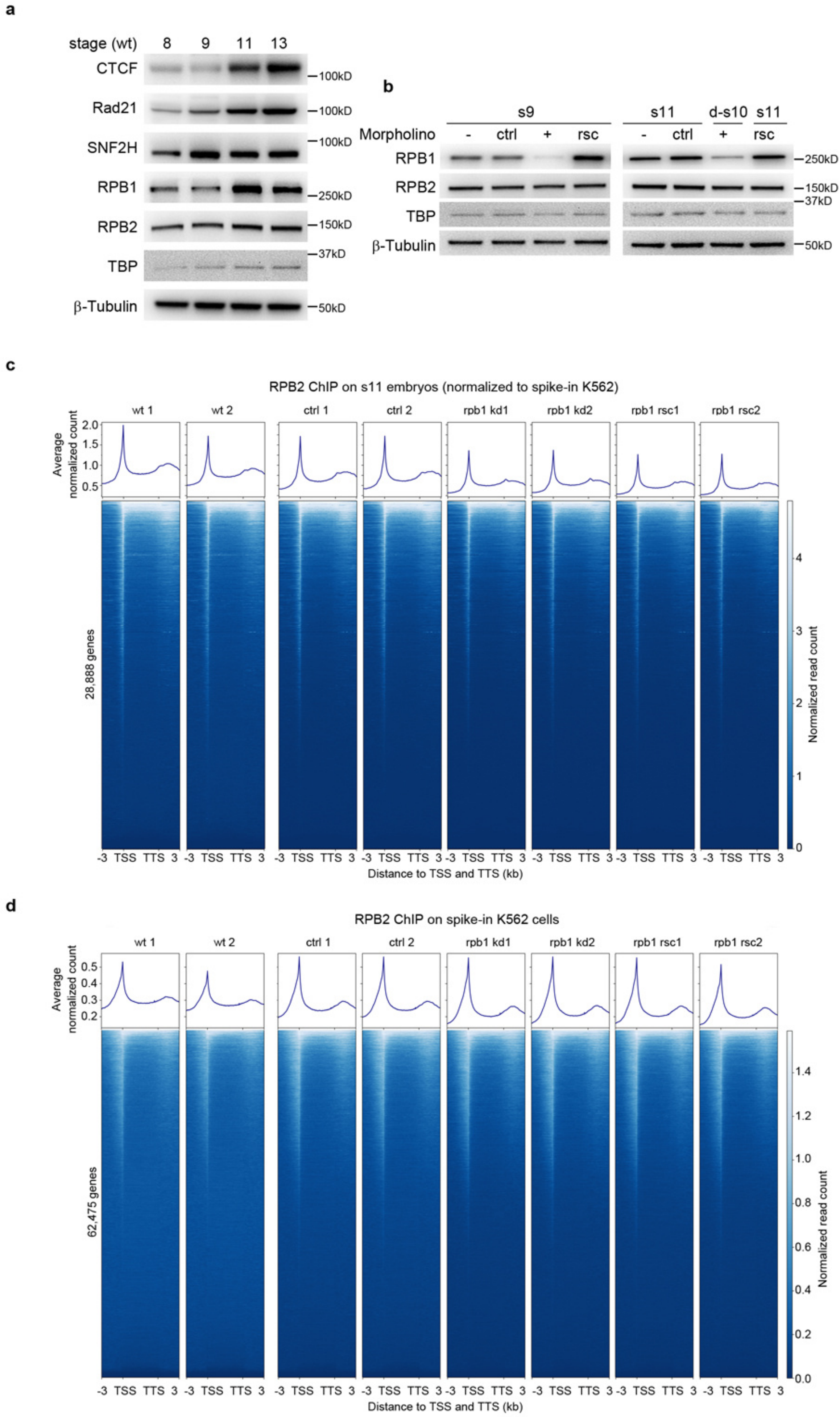

Supplementary Fig. 11

a

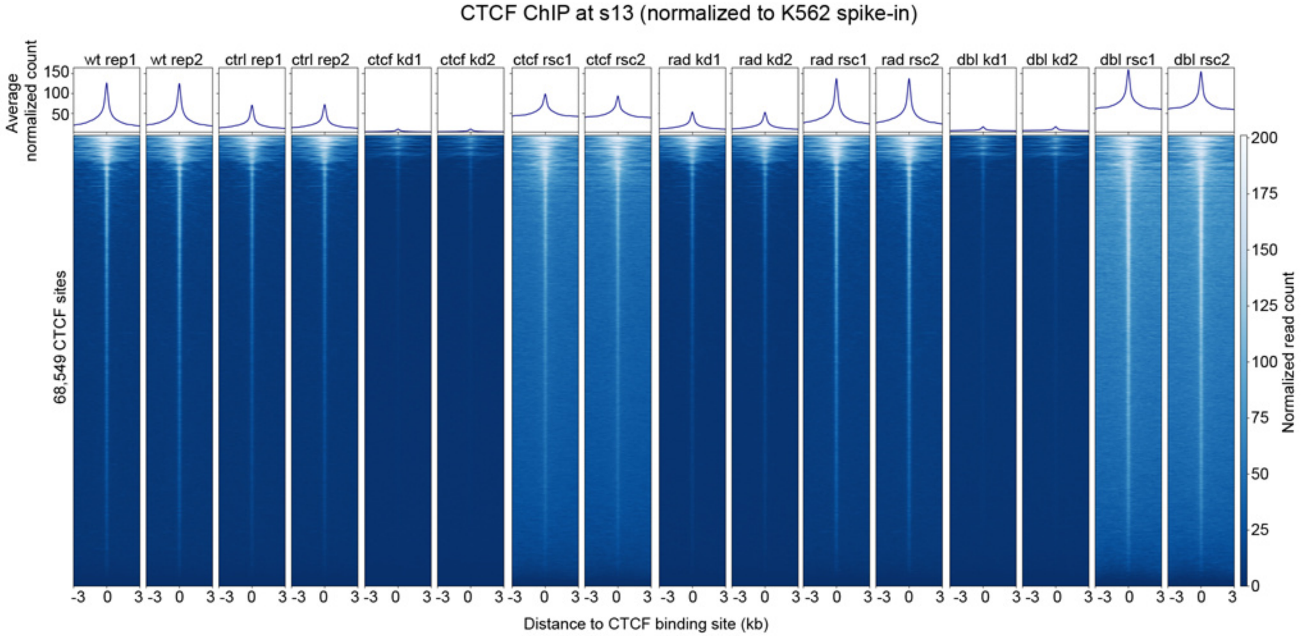

b

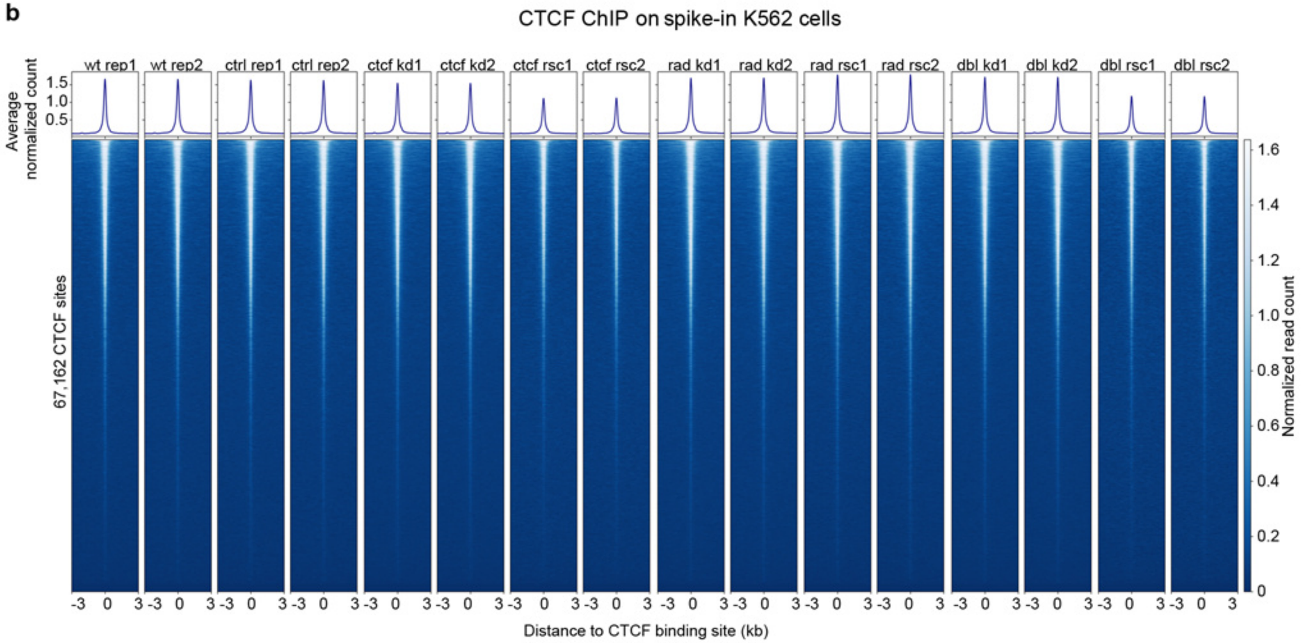

Supplementary Fig. 12

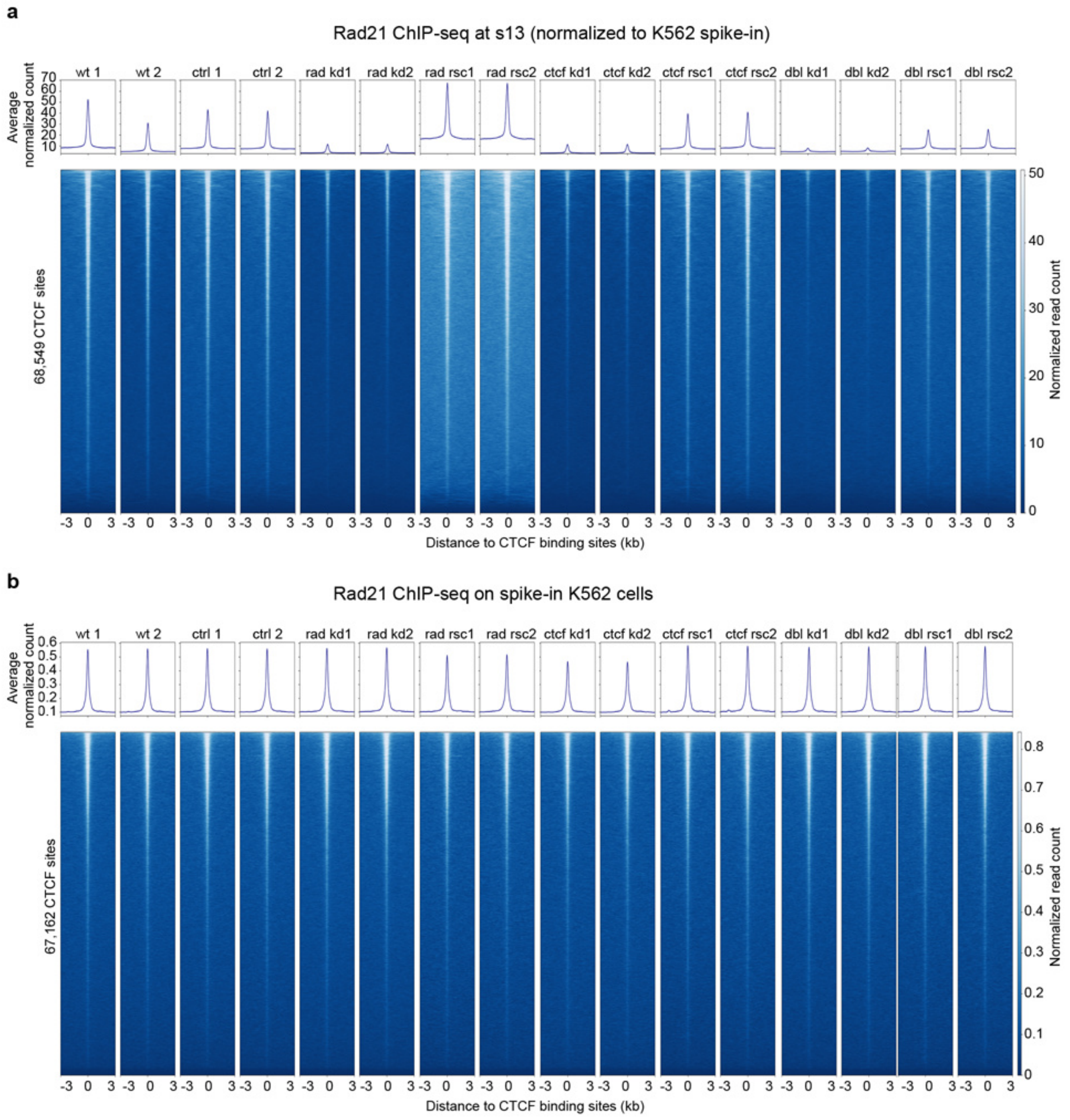

Supplementary Fig. 13

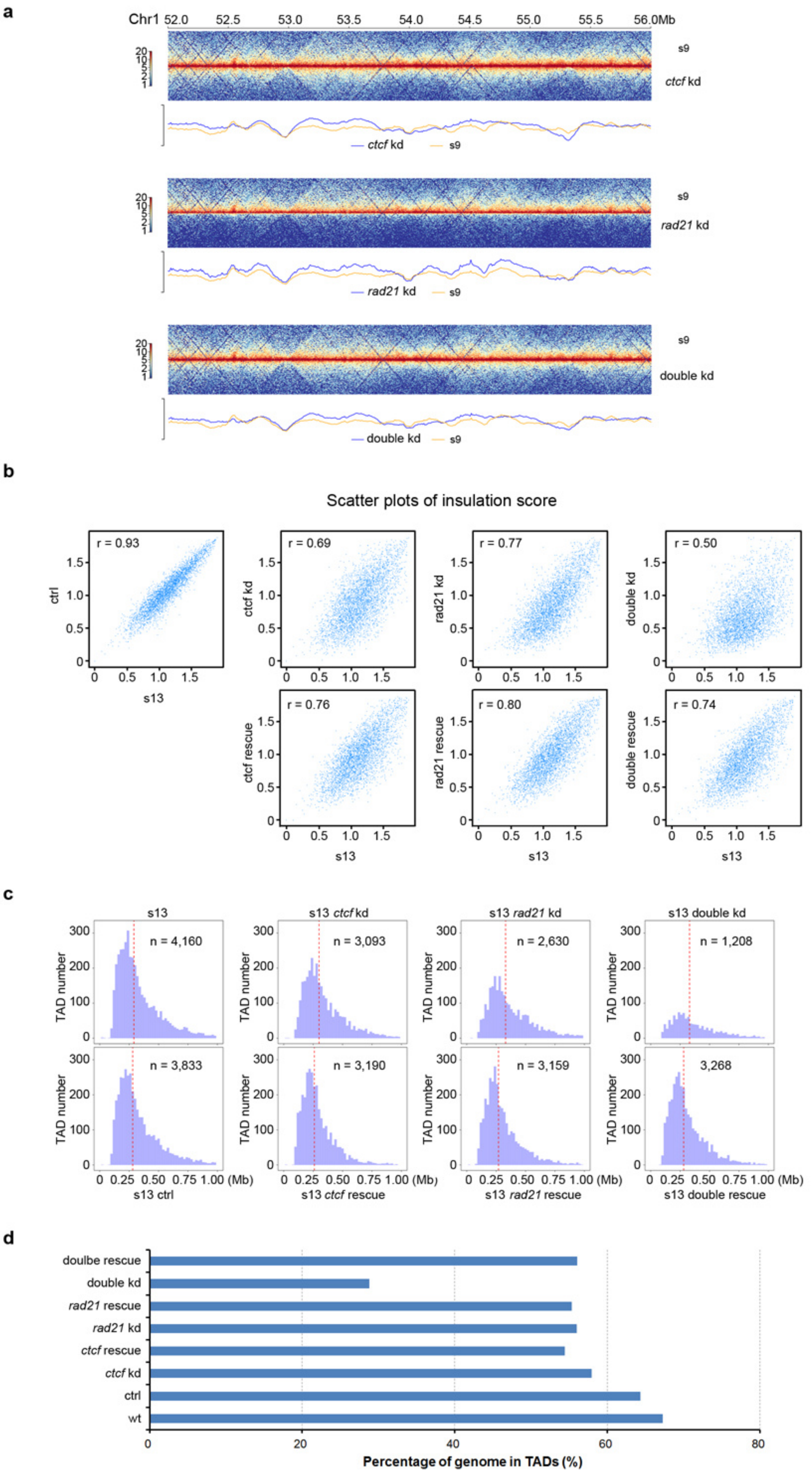

Supplementary Fig. 14

All embryos were at s13.

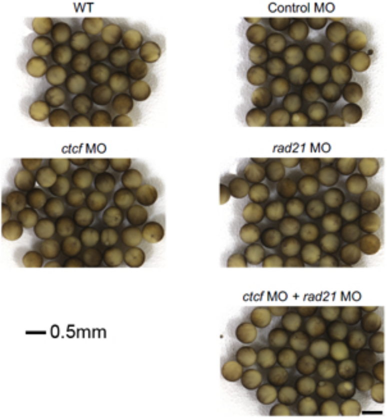

Supplementary Fig. 15

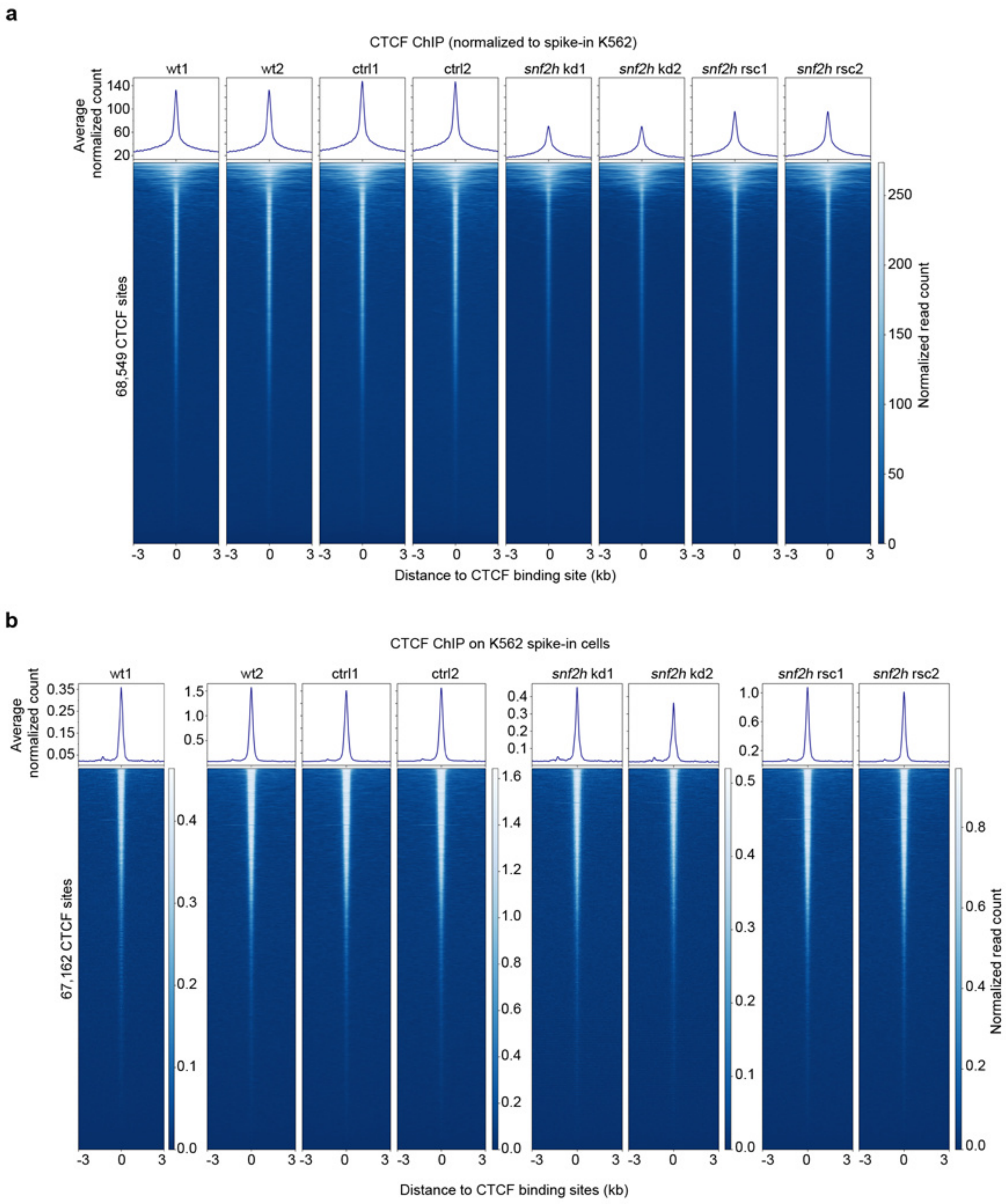

Supplementary Fig. 16

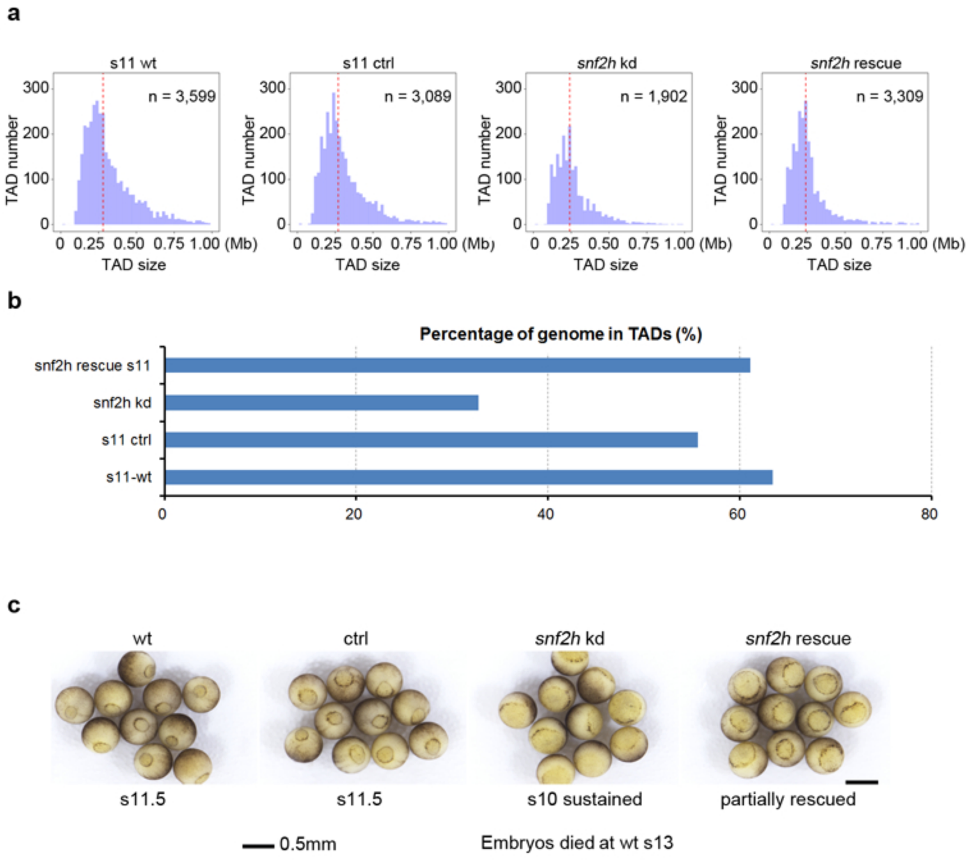

Supplementary Fig. 17

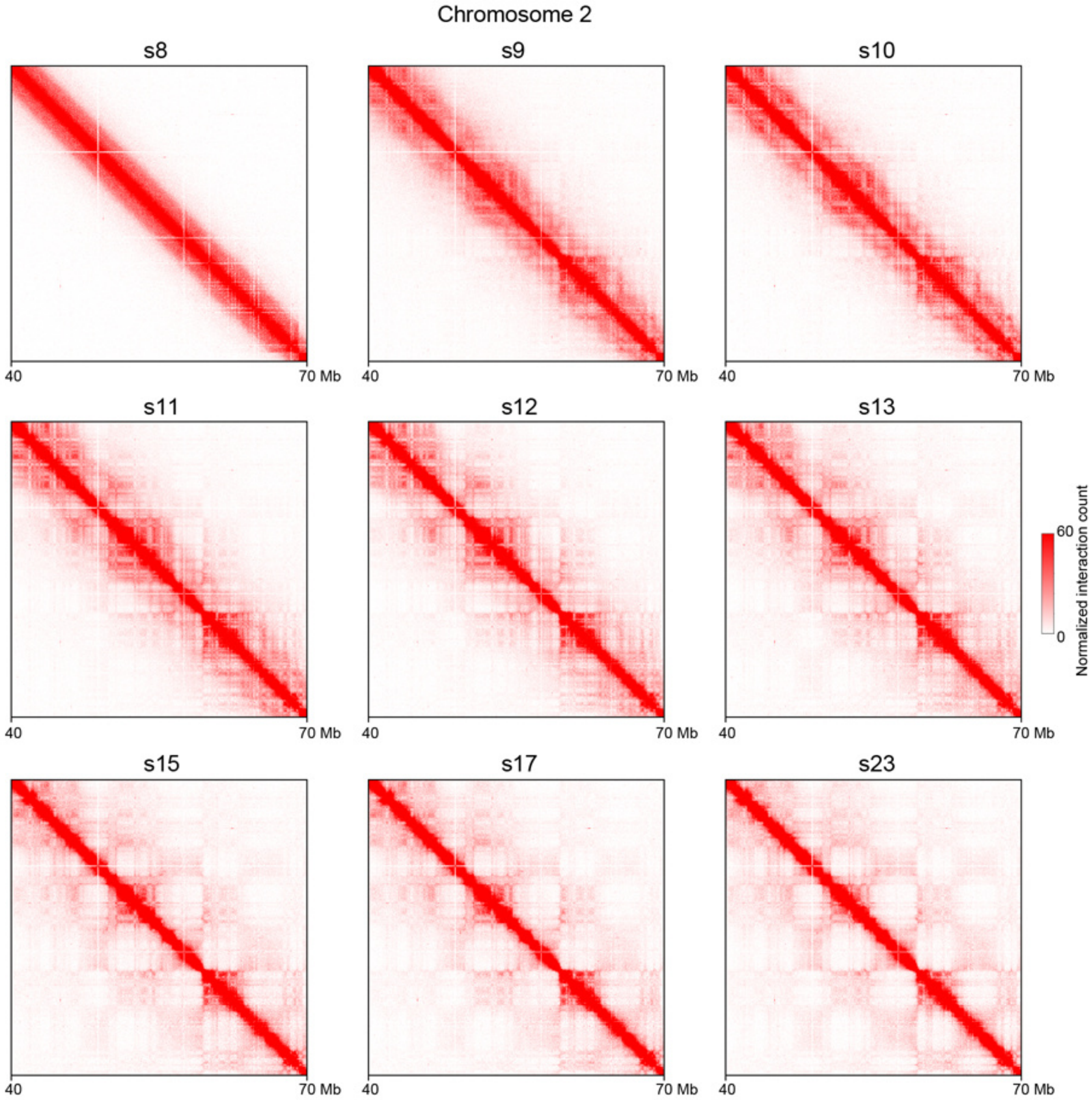

Supplementary Fig. 18

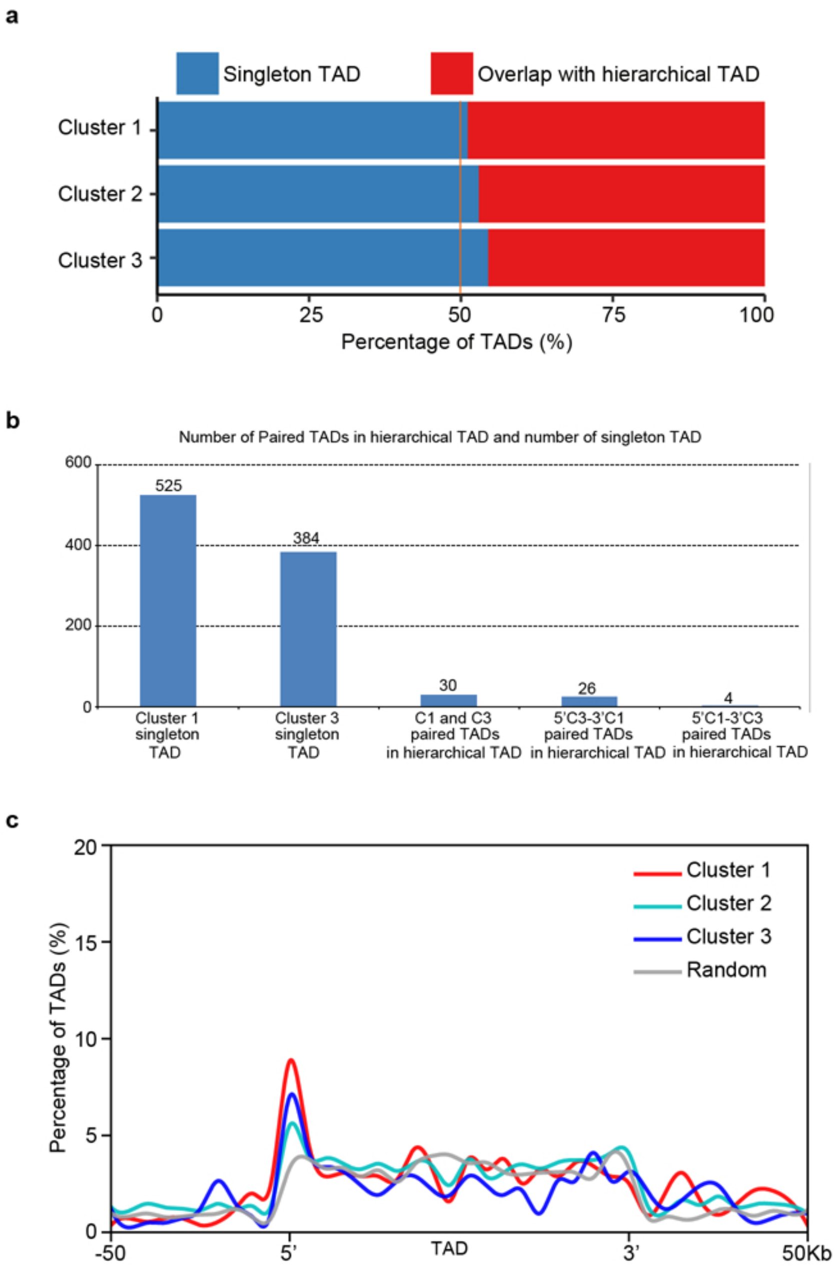

Supplementary Fig. 19

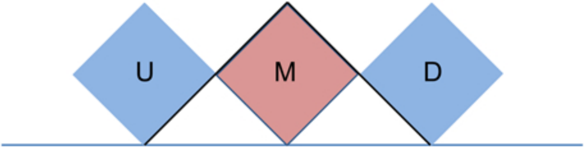

SourceData for Supplementary Fig. 3b

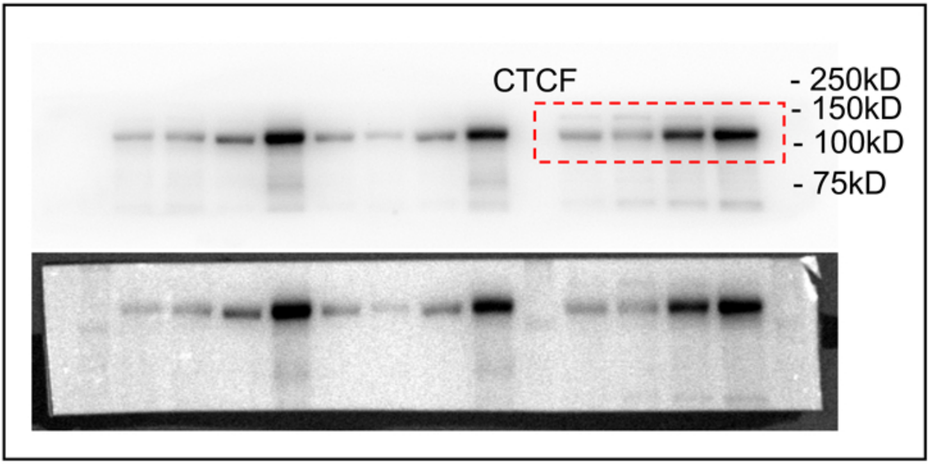

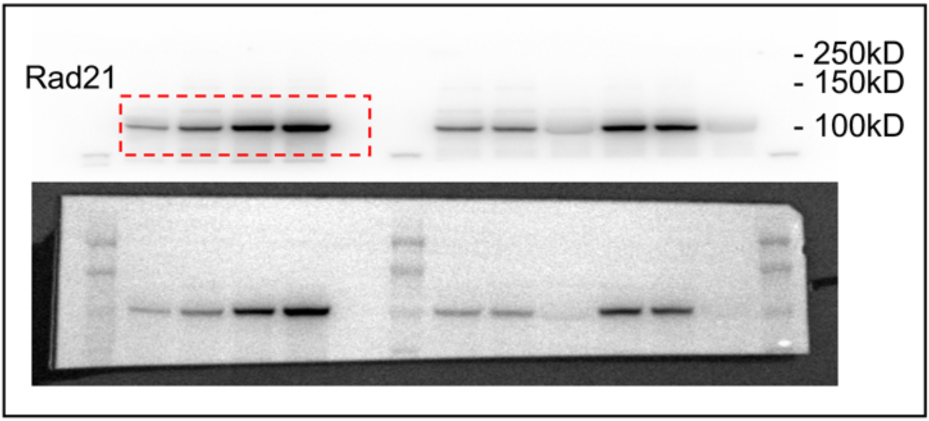

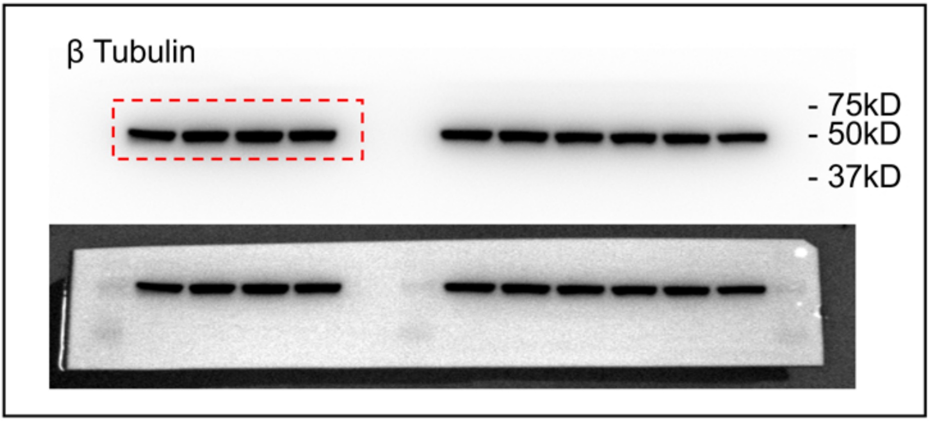

Supplementary Fig. 10a

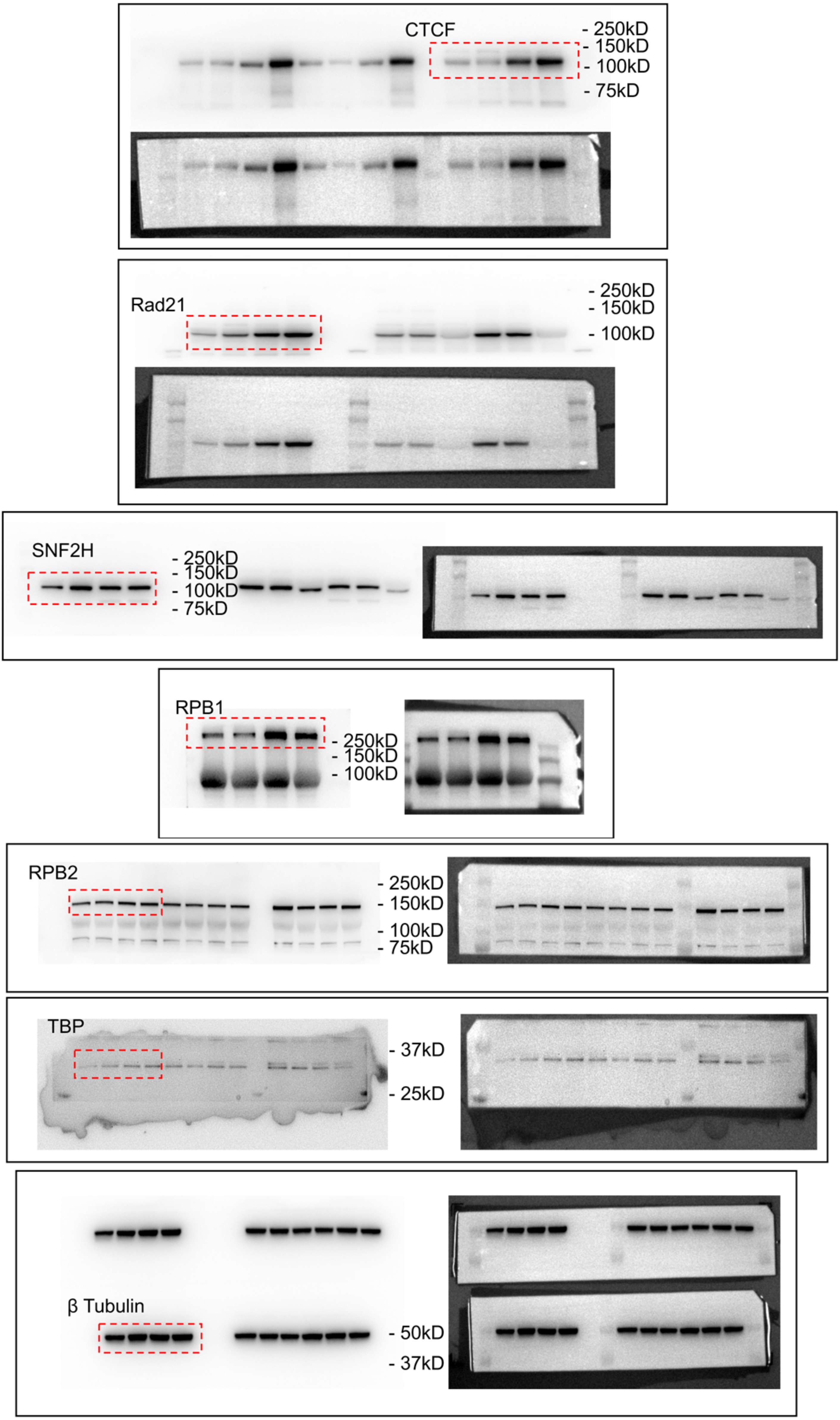

Supplementary Fig. 10b

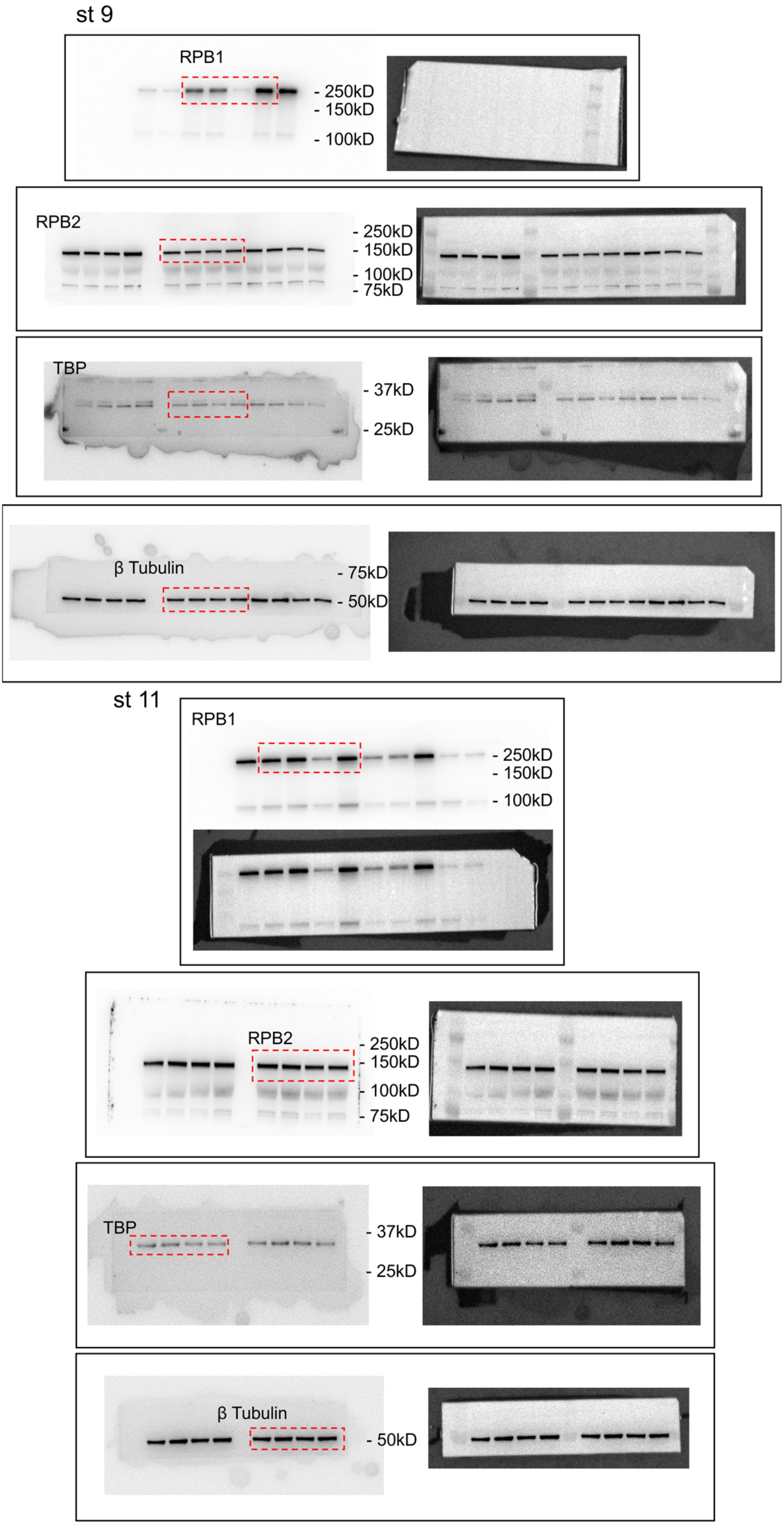

Supplement: Supplementary file 1 — Supplementary Figs. 1–19 and Source Data of western blots for Supplementary Figs. 3 and 10 [file 41588_2021_878_MOESM1_ESM.pdf]
