# Supplementary material for: Three-dimensional folding dynamics of the Xenopus tropicalis genome
Source: Nat Genet. 2021 Jun 7;53(7):1075–87. doi: 10.1038/s41588-021-00878-z (PMC8270788; doi:10.1038/s41588-021-00878-z)
Supplement: Supplementary file 2 — Reporting Summary [file 41588_2021_878_MOESM2_ESM.pdf]

nature research

Corresponding author(s): Chunhui Hou

Last updated by author(s): Apr 10, 2021

Reporting Summary

Nature Research wishes to improve the reproducibility of the work that we publish. This form provides structure for consistency and transparency in reporting. For further information on Nature Research policies, see Authors & Referees and the Editorial Policy Checklist.

Statistics

For all statistical analyses, confirm that the following items are present in the figure legend, table legend, main text, or Methods section.

n/a	Confirmed	
☐	☒	The exact sample size (*n*) for each experimental group/condition, given as a discrete number and unit of measurement
☒	☐	A statement on whether measurements were taken from distinct samples or whether the same sample was measured repeatedly
☒	☐	The statistical test(s) used AND whether they are one- or two-sided<br>*Only common tests should be described solely by name; describe more complex techniques in the Methods section.*
☒	☐	A description of all covariates tested
☒	☐	A description of any assumptions or corrections, such as tests of normality and adjustment for multiple comparisons
☐	☒	A full description of the statistical parameters including central tendency (e.g. means) or other basic estimates (e.g. regression coefficient) AND variation (e.g. standard deviation) or associated estimates of uncertainty (e.g. confidence intervals)
☒	☐	For null hypothesis testing, the test statistic (e.g. *F*, *t*, *r*) with confidence intervals, effect sizes, degrees of freedom and *P* value noted<br>*Give P values as exact values whenever suitable.*
☒	☐	For Bayesian analysis, information on the choice of priors and Markov chain Monte Carlo settings
☒	☐	For hierarchical and complex designs, identification of the appropriate level for tests and full reporting of outcomes
☐	☒	Estimates of effect sizes (e.g. Cohen's *d*, Pearson's *r*), indicating how they were calculated

Our web collection on statistics for biologists contains articles on many of the points above.

Software and code

Policy information about availability of computer code

Data collection	No commercial, open source or custom software was used to collect data in this study.
Data analysis	Computational analysis is described in the Methods. Custom code is also available at github.com/shenscore/Xenopus_Hi-C<br>R3.5, BWA version 0.7.15-r1140, Juicer (containing HICCUPS) 1.5, Juicebox (JBAT) 1.11.08, HGAP4 in SMRT Link (version 4.0.0), 3d-dna 170123, MAKER 2.31.10, MUMmer4.0, Bedtools version 2.29.0, Deeptools 3.1.3, MACS2 2.1.0, Samtools 1.9, Falcon, Arrow, ggplot2 3.3.0, ggbiplot 0.55, GPU Parallel 20150322, pyGenomeTracks 3.5, STARR version 2.7.1a

For manuscripts utilizing custom algorithms or software that are central to the research but not yet described in published literature, software must be made available to editors/reviewers. We strongly encourage code deposition in a community repository (e.g. GitHub). See the Nature Research guidelines for submitting code & software for further information.

Data

Policy information about availability of data

All manuscripts must include a data availability statement. This statement should provide the following information, where applicable:

- Accession codes, unique identifiers, or web links for publicly available datasets
- A list of figures that have associated raw data
- A description of any restrictions on data availability

All raw sequencing data generated in this study have been deposited in the BioProject database (http://www.ncbi.nlm.nih.gov/bioproject) under accession number PRJNA606649.
Processed ChIP-seq data and identified domains are available at https://doi.org/10.6084/m9.figshare.14377283.v2
H3K4me1, H3K4me3, H3K9me2, H3K27me3, H3K36me3, and p300 ChIP-seq data in X. tropicalis embryos are obtained from GEO “GSE67974”.
RNA-seq analysis during X. tropicalis embryonic development is obtained from GEO “GSE65785”.
CTCF ChIP-seq data in human K562 are obtained from ENCODE “ENCFF675GVW”.
Cohesin Rad21 ChIP-seq data in human K562 are obtained from ENCODE “ENCFF000YXZ”.

CTCF ChIP-seq data in Drosophila S2 are obtained from ENCODE "ENCFF512CQC".
Hi-C data in human K562 are obtained from GEO "GSE63525".
SAFE Hi-C data in Drosophila S2 are obtained from BioProject "PRJNA470784".
Xenbase version 10 genome assembly is also used for data analysis in this work.

Field-specific reporting

Please select the one below that is the best fit for your research. If you are not sure, read the appropriate sections before making your selection.

☒ Life sciences ☐ Behavioural & social sciences ☐ Ecological, evolutionary & environmental sciences

For a reference copy of the document with all sections, see nature.com/documents/nr-reporting-summary-flat.pdf

Life sciences study design

All studies must disclose on these points even when the disclosure is negative.

Sample size	No statistical methods were used to predetermine the sample size. We used hundreds of embryos at different developmental stages for Hi-Cs and ChIP-seq. At least two separate batches of embryos were used as biological replicates. These were determined based on the general variability of these data types in the literature and from our own experiences. The sample sizes are sufficient to reach conclusions.
Data exclusions	No data was excluded.
Replication	Hi-C and ChIP-seq were repeated for at least two times. Pearson's correlation analysis showed high reproducibility of these experiments. At least two replicates were carried out for western blot and with similar results.
Randomization	Samples were collected for experiments based on developmental stage. Each sample included hundreds of embryos of mixed-sex at the same developmental stage.
Blinding	Blinding was not carried out, because this study requires the knowledge of the genotype of the animal.

Reporting for specific materials, systems and methods

We require information from authors about some types of materials, experimental systems and methods used in many studies. Here, indicate whether each material, system or method listed is relevant to your study. If you are not sure if a list item applies to your research, read the appropriate section before selecting a response.

Materials & experimental systems

n/a	Involved in the study	
☐	☒	Antibodies
☒	☐	Eukaryotic cell lines
☒	☐	Palaeontology
☐	☒	Animals and other organisms
☒	☐	Human research participants
☒	☐	Clinical data

Methods

n/a	Involved in the study	
☐	☒	ChIP-seq
☒	☐	Flow cytometry
☒	☐	MRI-based neuroimaging

Antibodies

Antibodies used	monoclonal mouse anti-RPB1 from Biolegend, Cat-No. 664906 (1:3000 for western blot)<br>polyclonal rabbit anti-RPB2 from ABclonal, Cat-No. A5928 (1:3000 for western blot)<br>polyclonal rabbit anti-beta-tubulin from Abcam, Cat-No. ab6046 (1:3000 for western blot)<br>monoclonal mouse anti-histone H3 from Biodragon, Cat-No. B1055 (1:3000 for western blot)<br>polyclonal rabbit anti-Rad21 from Abcam, Cat-No. ab992 (1:3000 for western blot)<br>polyclonal rabbit anti-CTCF from Active motif, Cat-No. 61311 (1:3000 for western blot)<br>polyclonal rabbit anti-SNF2H from Biorbyt, Cat-No. orb154213 (1:3000 for western blot)<br>HRP conjugated anti-rabbit IgG from Transgen, Cat-No. HS101-01 (1:10000 for western blot)<br>HRP conjugated goat anti-mouse IgG from Transgen, Cat-No. HS201-01 (1:10000 for western blot)
Validation	Monoclonal mouse anti-RPB1 from Biolegend, Cat-No. 664906 was validated by manufacture for western blot, ChIP using human samples, and is reactive with yeast, wheat, mouse, C. elegans, X. laevis, and most other eukaryotic RNAPII.<br>Polyclonal rabbit anti-RPB2 from ABclonal, Cat-No. A5928 was validated by manufacture for western blot, ChIP using human, mouse, and rat samples. Sequence conservation analysis predicts its reactivity with other eukaryotic samples.<br>Polyclonal rabbit anti-Rad21 from Abcam, Cat-No. ab992 was validated by manufacture for western blot, IP using human, and mouse samples, and is predicted to react with rat, rabbit, horse, chichen, guinea pig, cow, dog, turkey, chimpanzee, gorilla, Chinese hamster, orangutan, elephant.<br>Polyclonal rabbit anti-CTCF from Active motif, Cat-No. 61311 was validated by manufacture for western blot, ChIP using human

	samples. Sequence conservation analysis predicts its reactivity with other eukaryotic samples.<br>Polyclonal rabbit anti-SNF2H from Biorbyt, Cat-No. orb154213 was validated by manufacture for western blot, ChIP using human, mouse, and rat samples.<br>Our western blot, knock-down experiment, ChIP-seq analysis further confirmed the specific reactivity of these antibodies (RPB1, RPB2, Rad21, CTCF, and SNF2H) to X. tropicalis samples. These antibodies give highly reproducible results for ChIP-seq and high specificity for western blot.<br>Polyclonal rabbit anti-beta-tubulin from Abcam, Cat-No. ab6046. This antibody was validated by manufacture for western blot using human samples, and was predicted to react with mouse, rat, chicken, pig, Xenopus, zebrafish, and Chinese hamster.<br>Monoclonal mouse anti-histone H3 from Biodragon, Cat-No. B1055. This antibody was validated by manufacture for western blot using human sample, and was predicted reactive with other eukaryotic samples including mouse, rat, and yeast. Conservation analysis also predicts reactivity with Xenopus samples.

Animals and other organisms

Policy information about studies involving animals; ARRIVE guidelines recommended for reporting animal research

Laboratory animals	Xenopus tropicalis frogs were purchased from Nasco (Fort Atkinson, WI, USA) and bred in an in-house facility. In vitro fertilization was carried out and developmental stages were determined according to Nieuwkoop and Faber. Male or female was not distinguished in this work. Ten pairs of male and female frogs of one year old were used for embryos production. Two male frog of one year old was used for tissue isolation.<br>Embryos were collected at developmental stages 8, 9, 10, 11, 12, 13, 15, 17, and 23. These samples were mixed-sex embryos.
Wild animals	No wild animals were used in the study.
Field-collected samples	No filed collected samples were used in the study.
Ethics oversight	All animal experiments were conducted in compliance with ethical guidelines. The study plan was approved by the Institutional Animal Care and Use Committee at the Southern University of Science and Technology, Shenzhen, Guangdong Province, China.

Note that full information on the approval of the study protocol must also be provided in the manuscript.

ChIP-seq

Data deposition

☒ Confirm that both raw and final processed data have been deposited in a public database such as GEO.

☒ Confirm that you have deposited or provided access to graph files (e.g. BED files) for the called peaks.

Data access links<br>*May remain private before publication.*	All raw ChIP-seq data generated in this study have been deposited in the BioProject database (http://www.ncbi.nlm.nih.gov/bioproject) under accession number PRJNA606649. Processed ChIP-seq data are available at https://doi.org/10.6084/m9.figshare.14377283.
Files in database submission	ChIP-seq input library<br>CTCF ChIP-seq on stage 8 embryos (with human K562 cells as spike-in control)<br>CTCF ChIP-seq on stage 9 embryos (with human K562 cells as spike-in control)<br>CTCF ChIP-seq on stage 11 embryos (with human K562 cells as spike-in control)<br>CTCF ChIP-seq on stage 13 embryos (with human K562 cells as spike-in control)<br>CTCF ChIP-seq on stage 13 embryos injected with control morpholinos (with human K562 cells as spike-in control)<br>CTCF ChIP-seq on stage 13 embryos injected with CTCF morpholinos (with human K562 cells as spike-in control)<br>CTCF ChIP-seq on stage 13 embryos injected with Rad21 morpholinos (with human K562 cells as spike-in control)<br>CTCF ChIP-seq on stage 13 embryos injected with CTCF and Rad21 morpholinos (with human K562 cells as spike-in control)<br>CTCF ChIP-seq on stage 13 embryos injected with CTCF morpholinos and also ctcf rescue expression RNA (with human K562 cells as spike-in control)<br>CTCF ChIP-seq on stage 13 embryos injected with Rad21 morpholinos and also rad21 rescue expression RNA (with human K562 cells as spike-in control)<br>CTCF ChIP-seq on stage 13 embryos injected with CTCF and Rad21 morpholinos and also ctct and rad21 rescue expression RNAs (with human K562 cells as spike-in control)<br>Rad21 ChIP-seq on stage 9 embryos (with human K562 cells as spike-in control)<br>Rad21 ChIP-seq on stage 11 embryos (with human K562 cells as spike-in control)<br>Rad21 ChIP-seq on stage 13 embryos (with human K562 cells as spike-in control)<br>Rad21 ChIP-seq on stage 13 embryos injected with control morpholinos (with human K562 cells as spike-in control)<br>Rad21 ChIP-seq on stage 13 embryos injected with Rad21 morpholinos (with human K562 cells as spike-in control)<br>Rad21 ChIP-seq on stage 13 embryos injected with CTCF morpholinos (with human K562 cells as spike-in control)<br>Rad21 ChIP-seq on stage 13 embryos injected with CTCF and Rad21 morpholinos (with human K562 cells as spike-in control)<br>Rad21 ChIP-seq on stage 13 embryos injected with CTCF morpholinos and also ctcf rescue expression RNA (with human K562 cells as spike-in control)<br>Rad21 ChIP-seq on stage 13 embryos injected with Rad21 morpholinos and also rad21 rescue expression RNA (with human K562 cells as spike-in control)<br>Rad21 ChIP-seq on stage 13 embryos injected with CTCF and Rad21 morpholinos and also ctct and rad21 rescue expression RNAs (with human K562 cells as spike-in control)<br>RPB1 ChIP-seq on wild type stage 11 embryos (with human K562 cells as spike-in control)<br>RPB1 ChIP-seq on stage 11 embryos injected with control morpholinos (with human K562 cells as spike-in control)<br>RPB1 ChIP-seq on delayed stage 10 embryos injected with rpb1 morpholinos (with human K562 cells as spike-in control)

	RPB1 ChIP-seq on rescued stage 11 embryos injected with rpb1 morpholinos and also rpb1 rescue expression RNA (with human K562 cells as spike-in control)<br>CTCF ChIP-seq on sustained stage 9 embryos injected with snf2h morpholinos (with human K562 cells as spike-in control)<br>CTCF ChIP-seq on rescued stage 11 embryos injected with snf2h morpholinos and also snf2h rescue expression RNA (with human K562 cells as spike-in control)<br>RPB2 ChIP-seq on wild type stage 11 embryos (with human K562 cells as spike-in control)<br>RPB2 ChIP-seq on stage 11 embryos injected with control morpholinos (with human K562 cells as spike-in control)<br>RPB2 ChIP-seq on delayed stage 10 embryos injected with rpb1 morpholinos (with human K562 cells as spike-in control)<br>RPB2 ChIP-seq on rescued stage 11 embryos injected with rpb1 morpholinos and also rpb1 rescue expression RNA (with human K562 cells as spike-in control)
Genome browser session (e.g. UCSC)	Not applicable.

Methodology

Replicates	All Hi-C and ChIP-seq experiments were performed for at least two biological replicates.
Sequencing depth	Hi-Cs were sequenced 150bp paired-ends with at least 200 million reads for each sample. ChIP-seq were sequenced 150bp paired-ends with at least 20 million reads each sample.
Antibodies	polyclonal rabbit anti-Rad21 from Abcam, Cat-No. ab992<br>polyclonal rabbit anti-CTCF from Active motif, Cat-No. 61311<br>monoclonal mouse anti-RPB1 from Biolegend, Cat-No. 664906<br>polyclonal rabbit anti-RPB2 from ABclonal, Cat-No. A5928
Peak calling parameters	Peaks were not specifically called. Enrichment was calculated.
Data quality	Signal tracks were calculated by using the 'bdgcmp' option of MACS 2.0 2.1.0 with the 'FE'(fold-enrichment) method.
Software	Mapping was performed using BWA version 0.7.15-r1140, reads were normalized (per million reads) and enrichment were calculated by MACS2 2.1.0.
