# Supplementary figures and images for: Three-dimensional folding dynamics of the Xenopus tropicalis genome
Source: Nat Genet. 2021 Jun 7;53(7):1075–87. doi: 10.1038/s41588-021-00878-z (PMC8270788; doi:10.1038/s41588-021-00878-z)

# Fig. 4a

Fig. 4b on next page

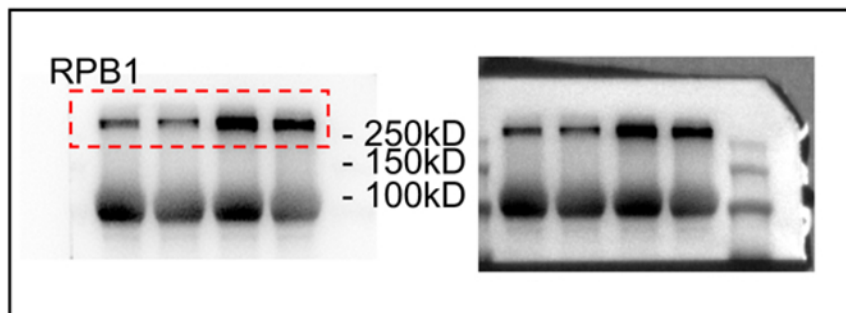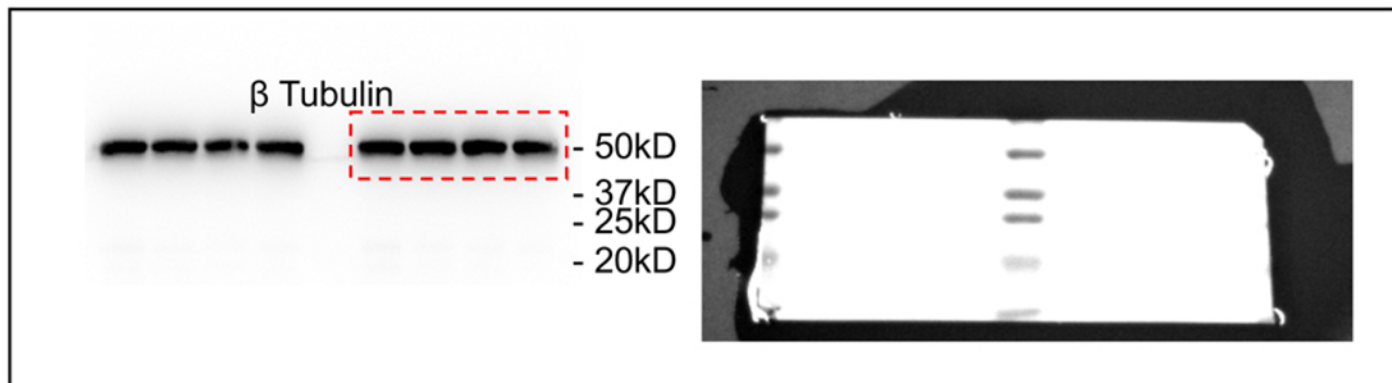

Fig. 4b

Fig. 4c on next page

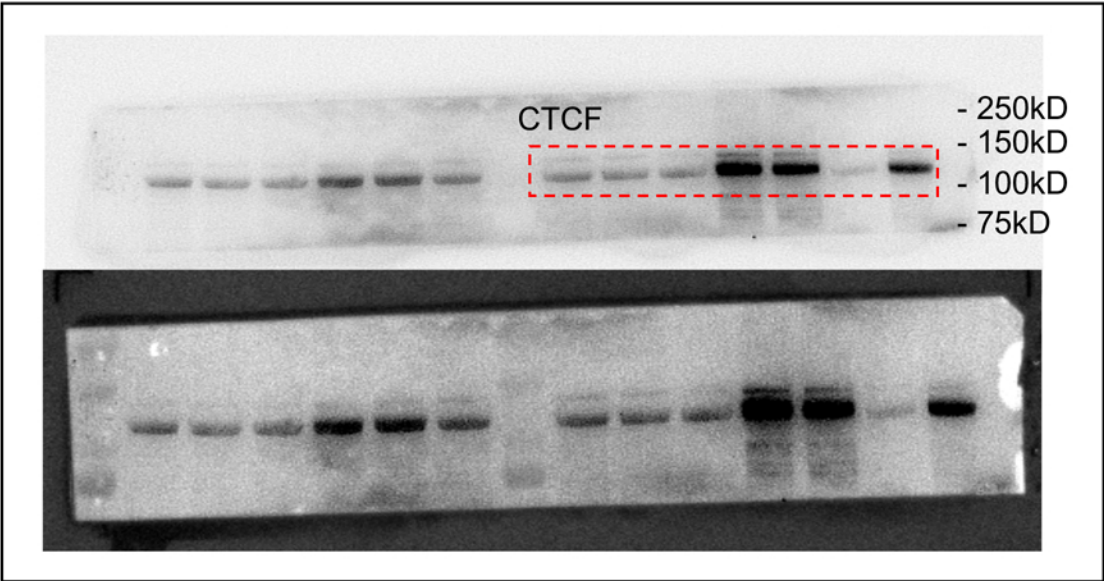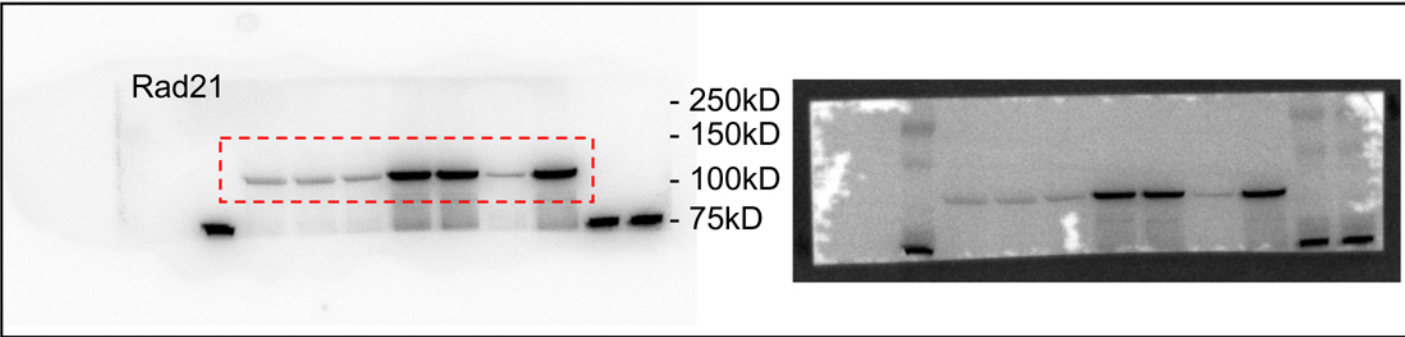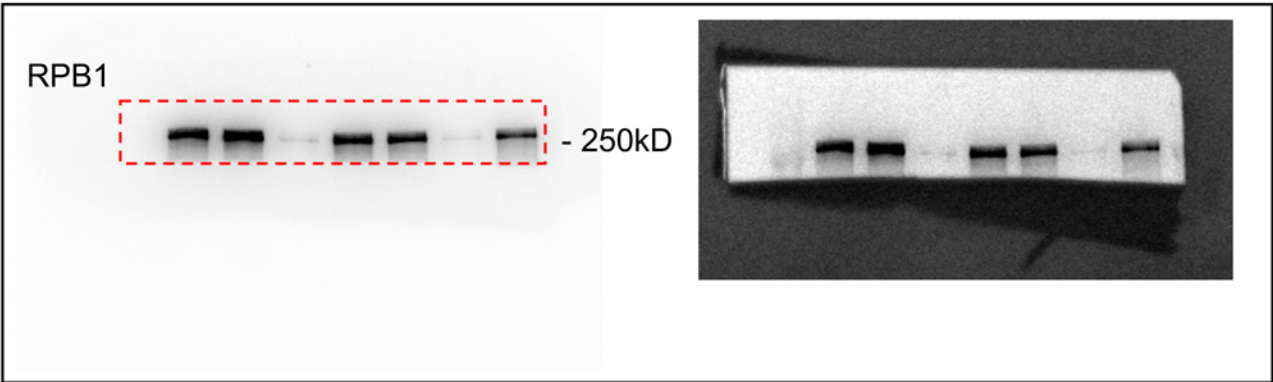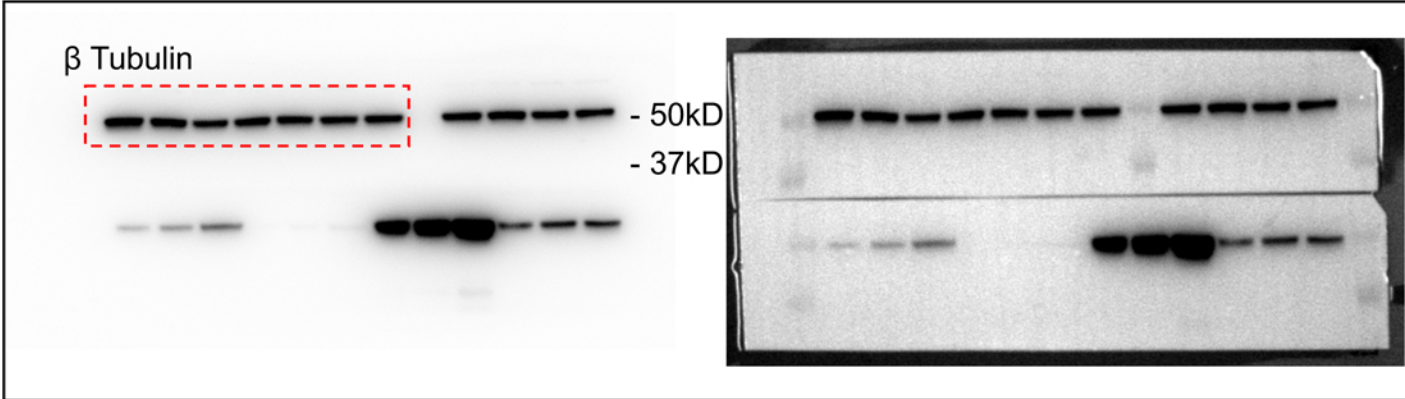

Fig. 4c

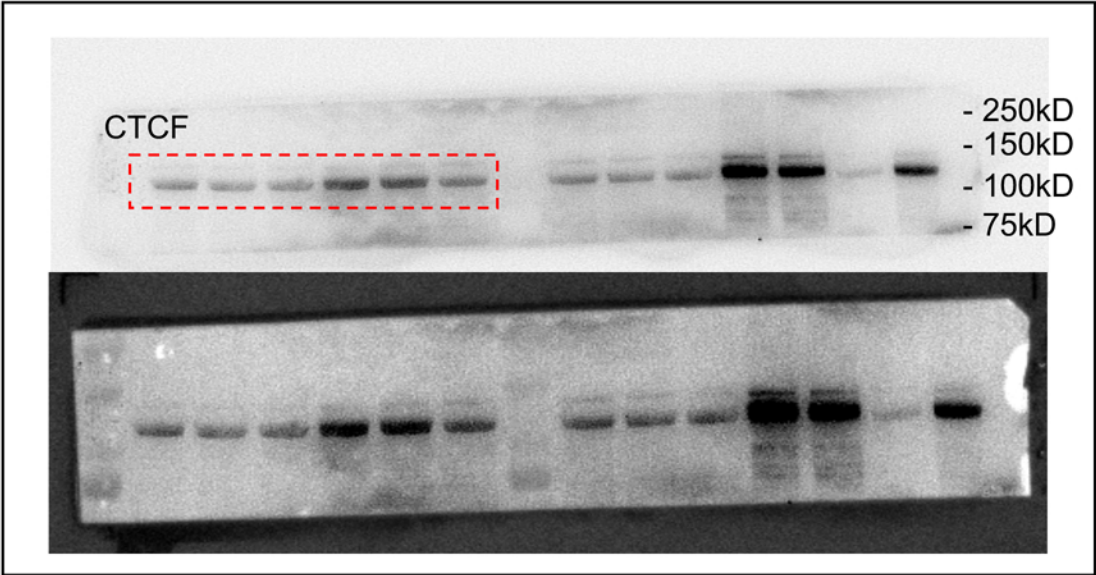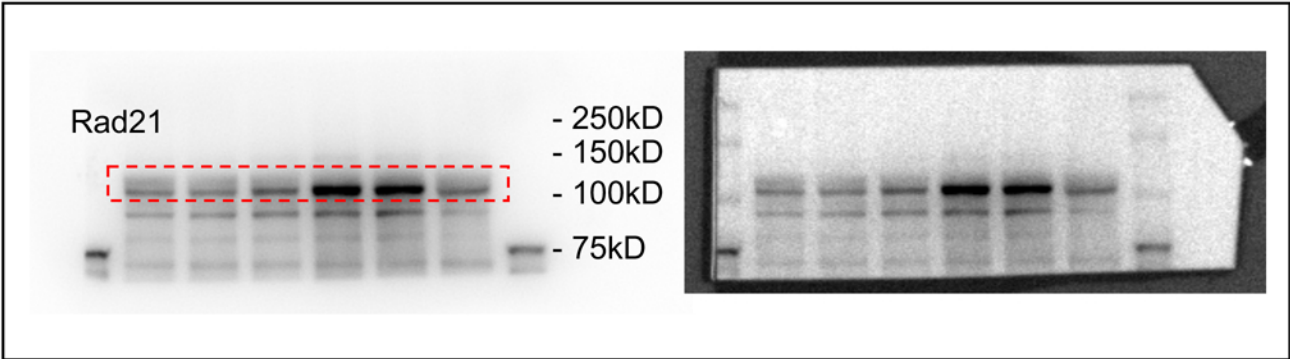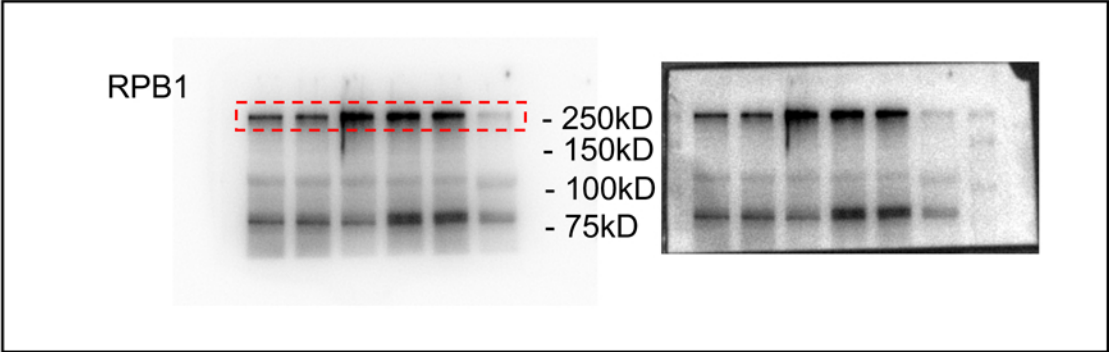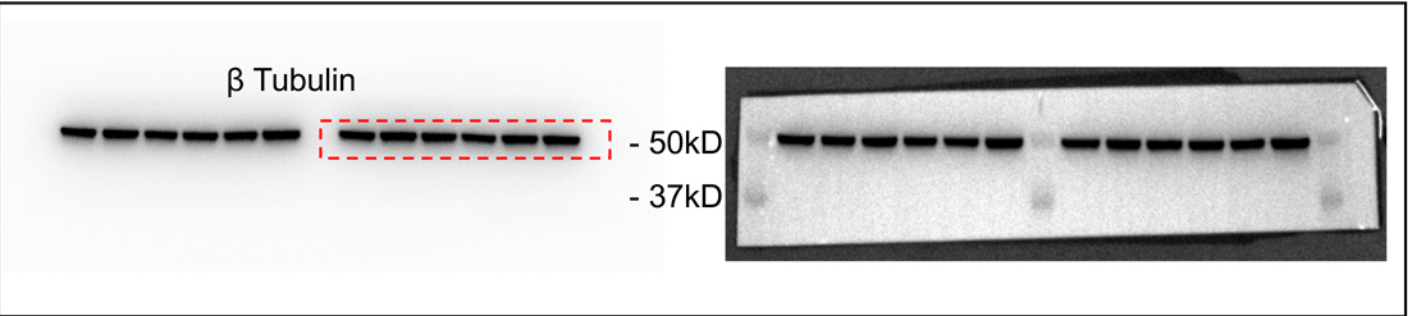

Supplement: Source Data Fig. 4 — Unprocessed western blots for Fig. 4. [file 41588_2021_878_MOESM4_ESM.pdf]

Fig. 5a

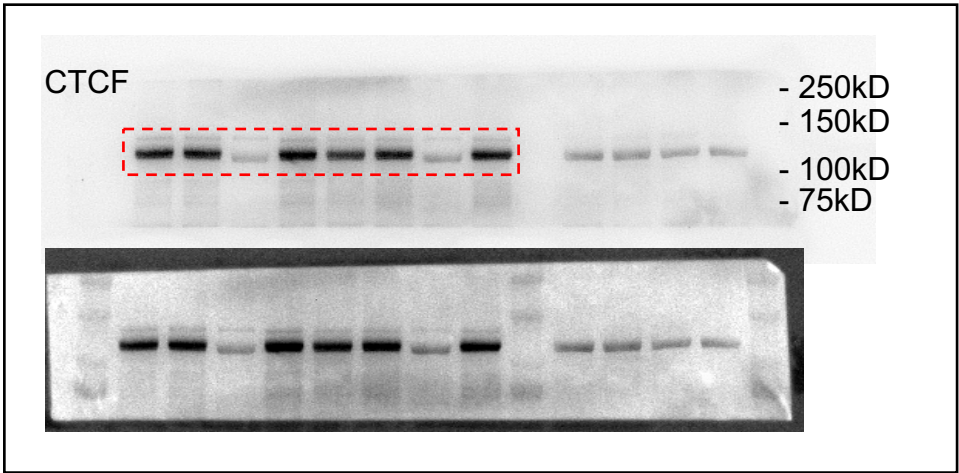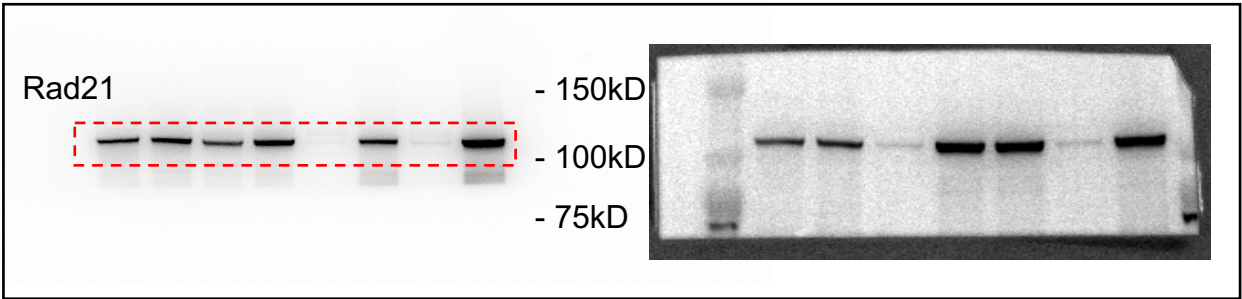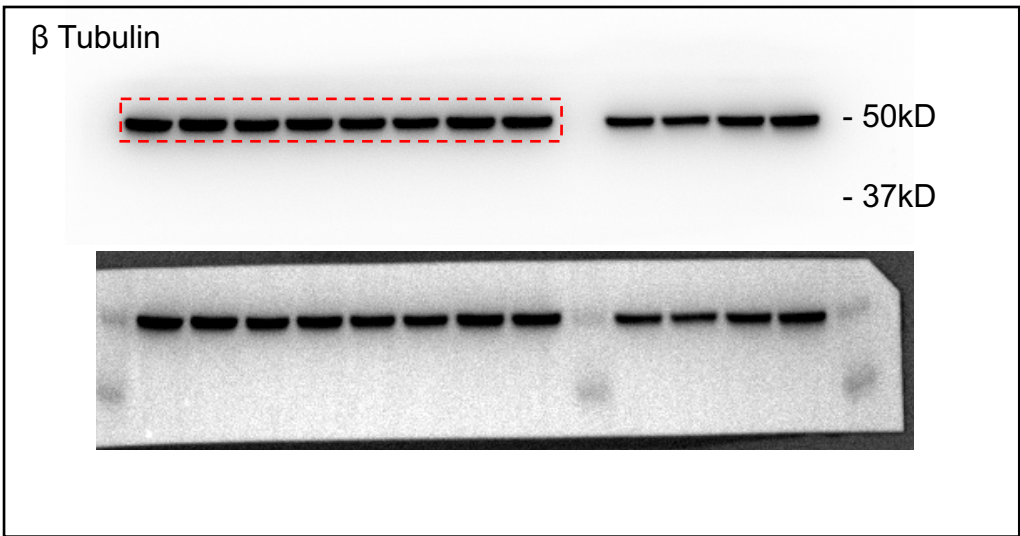

Supplement: Source Data Fig. 5 — Unprocessed western blots for Fig. 5. [file 41588_2021_878_MOESM5_ESM.pdf]

Fig. 6b

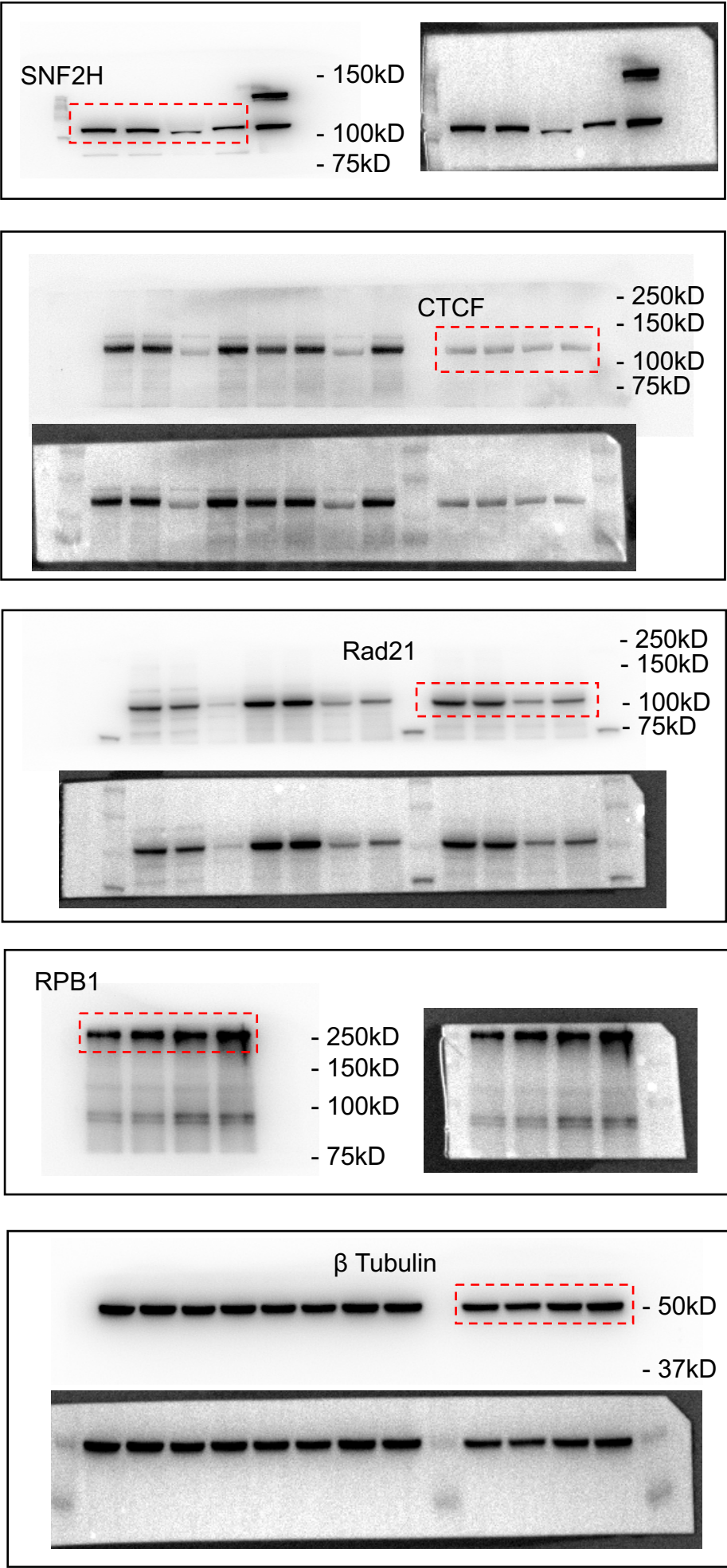

Supplement: Source Data Fig. 6 — Unprocessed western blots for Fig. 6. [file 41588_2021_878_MOESM6_ESM.pdf]

Fig. 8f

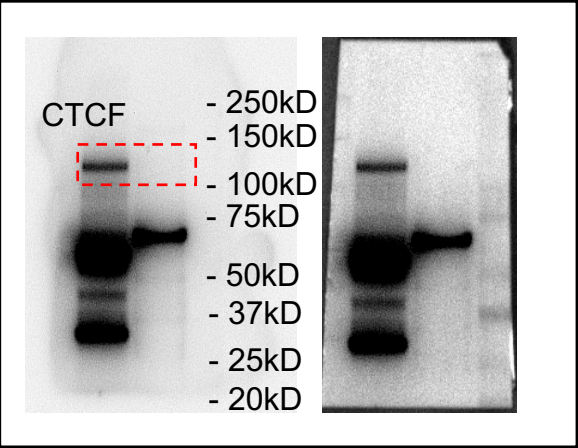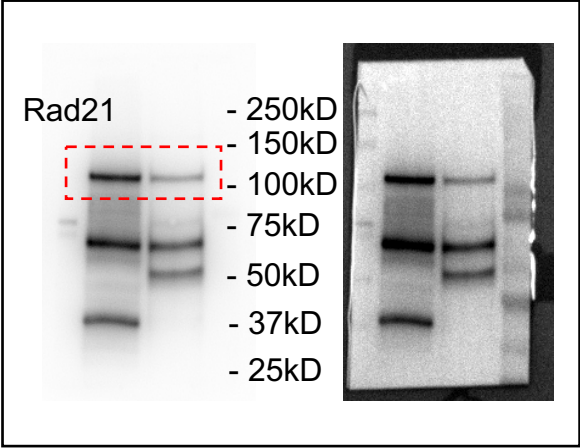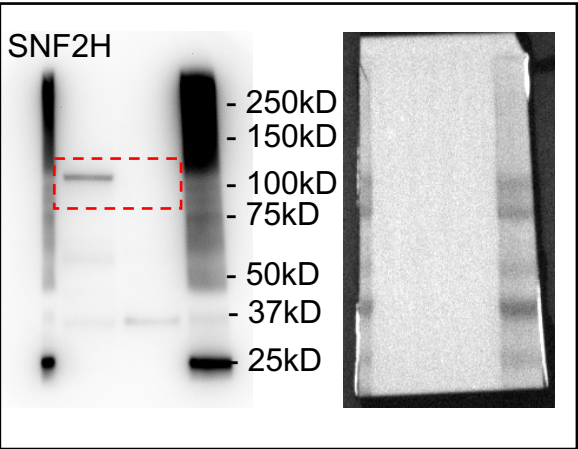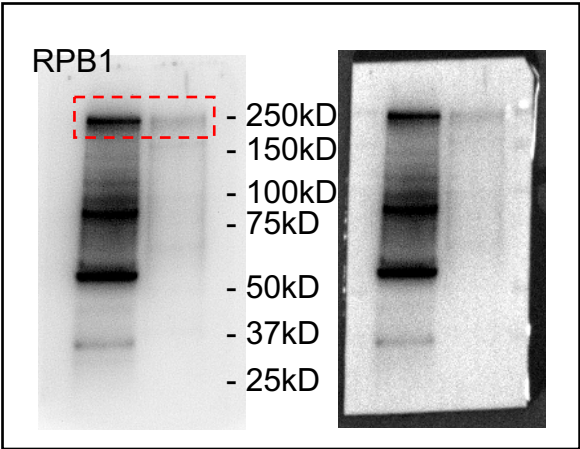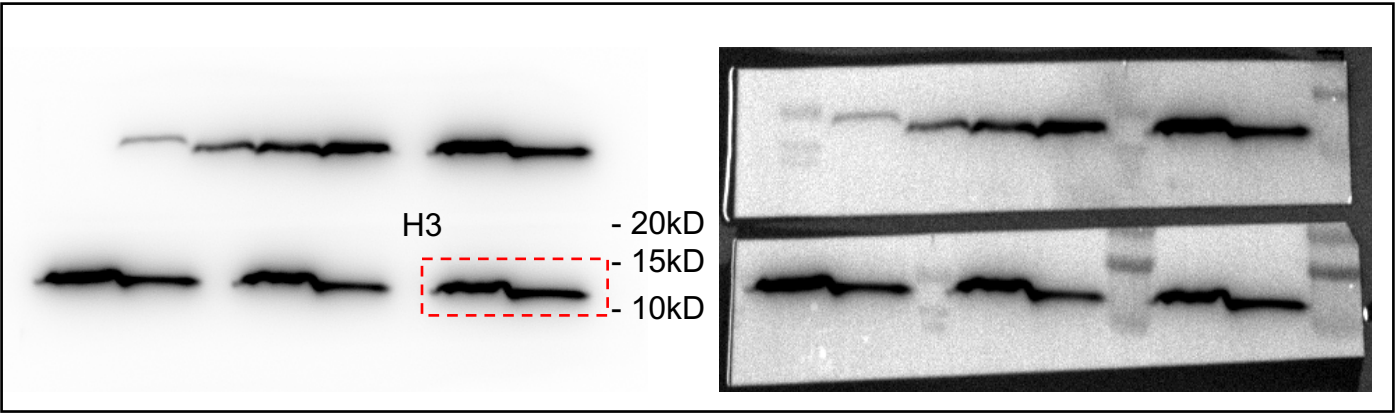

Supplement: Source Data Fig. 8 — Unprocessed western blots for Fig. 8. [file 41588_2021_878_MOESM7_ESM.pdf]
